# Attention-enhanced YOLOv8n-CLS for lightweight and efficient tomato leaf disease classification

**DOI:** 10.3389/fpls.2026.1917550

**Published:** 2026-10-14

**Authors:** Umesh Kumar Lilhore, J. Rexy, Kayal Padmanandam, Balakrishnan D., Rasmi A., Athinarayanan Subramanian, Hanaa A. Abdallah, Abdelaziz A. Abdelhamid, Ehab Seif Ghith, Aseel Smerat, Kaamran Raahemifar, Sarita Simaiya

**Affiliations:** 1 Department of Computer Science and Engineering, Galgotias University, Greater Noida, India; 2 Department of Computer Science, St. Xavier’s College, Tirunelveli, Tamil Nadu, India; 3 Department of IT, BVRIT Hyderabad College of Engineering for Women, Hyderabad, India; 4 Department of Computer Science and Engineering, Kalasalingam Academy of Research and Education, Krishnankoil, Tamilnadu, India; 5 Department of Computer Science and Engineering (IoT & Cyber Security including Blockchain Technology), RR Institute of Technology, Bangalore, Karnataka, India; 6 Department of Computer Science and Engineering, School of Computing, Vel Tech Rangarajan Dr. Sagunthala R&D Institute of Science and Technology, Avadi, India; 7 Department of Information Technology, College of Computer and Information Sciences, Princess Nourah bint Abdulrahman University, Riyadh, Saudi Arabia; 8 Department of Computer Science, College of Computing and Information Technology, Shaqra University, Shaqra, Saudi Arabia; 9 Department of Computer Science, Faculty of Computer and Information Sciences, Ain Shams University, Cairo, Egypt; 10 Department of Mechatronics, Faculty of Engineering, Ain shams University, Cairo, Egypt; 11 Hourani Center for Applied Scientific Research, Al-Ahliyya Amman University, Amman, Jordan; 12 College of Information Sciences and Technology, Penn State University, University Park, State College, PA, United States

**Keywords:** cross-dataset evaluation, deep learning, image-based disease screening, lightweight spatial attention, near-real-time classification, precision agriculture, tomato leaf disease classification, YOLOv8n-CLS

## Abstract

**Introduction:**

Tomato leaf diseases substantially reduce crop yield and fruit quality, creating a need for accurate, computationally efficient, and potentially deployable image-based disease-screening systems for precision agriculture. This study presents an Attention-Enhanced YOLOv8n-CLS framework for tomato leaf disease classification by integrating a lightweight spatial attention mechanism to enhance the representation of spatially informative image features while maintaining a compact computational structure.

**Methods:**

Unlike conventional object detection approaches that require bounding-box annotations, the proposed framework formulates disease recognition as an image-level classification task, requiring only class labels and thereby simplifying dataset preparation and model training. Experiments were conducted on two publicly available benchmark datasets, namely the PlantVillage Tomato Dataset and the Kaggle Tomato Leaf Disease Dataset. A systematic evaluation comprising binary and multiclass classification, five-fold cross-validation, ablation analysis, cross-dataset evaluation, comparative benchmarking, computational assessment, and Grad-CAM-based visualization was performed to assess classification performance, stability, transferability, and computational efficiency.

**Results:**

The Attention-Enhanced YOLOv8n-CLS achieved a maximum classification Accuracy of 98.90%, an AUC of 0.991, a computational complexity of 7.5 GFLOPs, and an average inference speed of approximately 45 FPS on the evaluated computing platform. In five-fold cross-validation, the proposed model achieved a mean Accuracy of 98.50% ± 0.14% on the PlantVillage Tomato Dataset and 97.82% ± 0.29% on the Kaggle Tomato Leaf Disease Dataset. The model also demonstrated competitive performance relative to representative deep learning architectures, including ResNet50, DenseNet201, ResNet152V2, MobileNetV2, and YOLOv5. The cross-dataset experiments further demonstrated transferability between the evaluated PlantVillage and Kaggle image distributions using the 10 classes shared by the two datasets.

**Discussion:**

The results indicate a favourable balance between classification performance and computational requirements under the evaluated experimental conditions, with potential for further investigation in resource-constrained applications. Grad-CAM visualization provided qualitative information about image regions associated with the model's classification decisions but did not independently establish biological causality or pathogen-specific recognition. Importantly, the proposed framework performs image-based symptom classification and preliminary disease screening using dataset-provided labels; it does not independently confirm causal pathogens. Therefore, the reported classification performance should not be interpreted as definitive pathogen diagnosis or field-level validation. Further evaluation using independently collected field images, laboratory-confirmed disease or pathogen status, diverse symptom presentations, and representative agricultural edge devices is required before practical agricultural deployment can be established.

## 1 Introduction

Tomato (*Solanum lycopersicum*) is one of the world’s most widely cultivated vegetable crops and plays an important role in global food production because of its nutritional value and economic importance (Chen et al., 2025). Tomatoes are rich in vitamins, minerals, antioxidants, and other bioactive compounds and therefore contribute substantially to human nutrition and agricultural economies. However, tomato production is affected by numerous diseases, including bacterial spot, early blight, late blight, leaf mold, septoria leaf spot, tomato mosaic virus, and tomato yellow leaf curl virus (TYLCV) (Long and Zhou, 2025; Sun et al., 2025). These diseases can reduce crop yield and fruit quality and increase production costs, thereby affecting agricultural productivity and food security (Agarwal et al., 2020; Thangaraj et al., 2022; Chen et al., 2025; Sun et al., 2025).

Early and accurate disease screening is important for supporting timely crop-management decisions, although definitive identification of a causal pathogen may require laboratory-based diagnostic methods (Zhu et al., 2025). Visual symptoms can provide useful indicators for preliminary screening; however, definitive pathogen identification may require molecular, serological, microbiological, or other diagnostic approaches. Accordingly, image-based classification systems should be regarded as complementary screening and decision-support tools rather than substitutes for laboratory confirmation. In conventional agricultural practice, disease classification often relies on visual assessment by farmers or plant pathologists. Although manual assessment remains widely used, it can be subjective, time-consuming, and dependent on specialized expertise (Bellout et al., 2025; Yan and Li, 2025). Furthermore, different diseases may exhibit visually similar symptoms, particularly during certain stages of disease development, which can complicate consistent classification (Paymode et al., 2021; Thangaraj et al., 2022; Sanida et al., 2023). The increasing scale of agricultural production further limits the feasibility of continuous manual monitoring and has motivated the development of automated and scalable plant disease classification systems (Thangaraj et al., 2022; Sanida et al., 2023).

Recent advances in artificial intelligence, computer vision, and deep learning have substantially improved automated plant disease recognition. Convolutional neural networks (CNNs) have demonstrated the ability to learn discriminative visual representations directly from leaf images without requiring handcrafted feature extraction (Agarwal et al., 2020; Abdullah et al., 2024; Bakır, 2024; Khan et al., 2024; Shanthi et al., 2024). Numerous deep learning architectures, including ResNet, DenseNet, MobileNet, transfer-learning frameworks, ensemble models, recurrent neural networks, and hybrid CNN-transformer architectures, have been applied to tomato leaf disease classification with promising results (Bakır, 2024; Javidan et al., 2024; Khan et al., 2024; Shanthi et al., 2024; Sreedevi and Manike, 2024). Comprehensive review studies have further highlighted the increasing role of deep learning in automated plant disease classification because of its ability to learn complex visual patterns from image data (Thangaraj et al., 2022; Seth et al., 2023).

More recently, the You Only Look Once (YOLO) family of architectures has attracted increasing attention in agricultural computer-vision applications because of its combination of predictive performance and computational efficiency. Although YOLO was originally developed primarily for object detection, YOLO-based architectures have also been adapted for image-classification applications and tomato disease recognition through architectural modifications, feature fusion, multi-scale learning, and attention mechanisms (Bellout et al., 2025; Chen et al., 2025; Liu et al., 2025; Ramos and Sappa, 2025; Shen et al., 2025; Wang et al., 2025; Yan and Li, 2025). Chen et al. (2025) proposed an improved YOLOv8-based approach for tomato disease recognition, whereas Bellout et al. (2025) developed a lightweight YOLOv10n framework oriented toward IoT-based disease monitoring. Liu et al. (2025) introduced YOLO-BSMamba to improve classification robustness under complex background conditions, while Ramos and Sappa (2025) presented a comparative analysis of YOLO architectures for tomato leaf disease-category classification. These studies demonstrate the potential of lightweight YOLO-based architectures for image-based agricultural disease recognition.

Despite these advances, several challenges remain. First, many existing YOLO-based plant disease studies formulate the task as object detection and therefore require manually annotated bounding boxes in addition to disease labels (Ayyad et al., 2025; Bellout et al., 2025; Chen et al., 2025; Wang et al., 2025). However, widely used benchmark datasets, including the PlantVillage Tomato Dataset and the Kaggle Tomato Leaf Disease Dataset, primarily provide image-level class annotations rather than object-level localization annotations (Hughes and Salathé, 2015; Motwani, 2019). When the primary objective is image-level disease classification, requiring bounding-box annotations can increase annotation effort without necessarily providing additional information required for the classification task. An image-level classification formulation can therefore provide a simpler alternative when localization is not the primary objective.

Second, although many deep learning models achieve high classification performance, some employ increasingly complex network architectures, multi-branch feature-fusion mechanisms, or computationally intensive attention modules that can increase parameter count, memory requirements, and inference cost (Dhiman et al., 2023; Wang et al., 2024; Bellout et al., 2025; Liu et al., 2025; Sun et al., 2025; Yan and Li, 2025). Such computational requirements may present challenges for applications involving resource-constrained platforms, including smartphones, unmanned aerial vehicles, and embedded agricultural monitoring systems (Le et al., 2024; Bellout et al., 2025; Wei et al., 2026). Consequently, developing models that maintain strong classification performance while controlling computational complexity remains an important research objective.

Third, attention mechanisms have been widely investigated for improving feature representation by assigning greater importance to informative spatial or channel-level features (Hu et al., 2018; Woo et al., 2018; Hou et al., 2021; Wang et al., 2024; Sun et al., 2025). However, the integration of additional attention components can increase model complexity and computational requirements. A lightweight attention mechanism that improves classification performance without substantially increasing computational cost may therefore be advantageous for efficient image-based disease classification (Ramos and Sappa, 2025; Wang et al., 2025).

In addition, many existing studies primarily emphasize aggregate classification performance while providing comparatively limited analysis of computational efficiency, model complexity, cross-dataset transferability, statistical stability, and interpretability (Liu et al., 2025). Comprehensive evaluation incorporating independent test-set performance, cross-validation, computational benchmarking, cross-dataset evaluation, ablation analysis, and qualitative interpretability remains important for determining the practical characteristics and limitations of image-based plant disease classification systems (Ramos and Sappa, 2025; Ruhad et al., 2025; Wei et al., 2026).

Motivated by these challenges, this study presents an Attention-Enhanced YOLOv8n-CLS framework for image-based tomato leaf disease classification. Rather than treating disease recognition as an object-detection problem, the proposed framework formulates the task as image-level classification, thereby requiring only class-level annotations and avoiding the need for bounding-box labeling. A lightweight spatial attention module is incorporated into the YOLOv8n-CLS architecture to enhance the representation of spatially informative image features while maintaining a compact computational structure. The study focuses on evaluating the balance between classification performance and computational requirements rather than claiming direct edge-device deployment. The model is systematically evaluated on the PlantVillage Tomato Dataset and the Kaggle Tomato Leaf Disease Dataset using independent test sets, five-fold cross-validation, comparative benchmarking, ablation analysis, computational assessment, cross-dataset evaluation, and Grad-CAM-based visualization. The main contributions of this study are summarized as follows:

- An Attention-Enhanced YOLOv8n-CLS framework is developed for image-based tomato leaf disease classification using image-level annotations, thereby avoiding the need for bounding-box labeling when disease localization is not the primary objective.
- A lightweight spatial attention mechanism is integrated into the classification architecture to enhance the representation of spatially informative image features while maintaining a relatively compact computational profile.
- A comprehensive experimental evaluation is conducted using the PlantVillage Tomato Dataset and the Kaggle Tomato Leaf Disease Dataset. The evaluation includes binary and multiclass classification, five-fold cross-validation, independent test-set evaluation, cross-dataset transfer, ablation analysis, and comparative benchmarking against ResNet50, DenseNet201, ResNet152V2, MobileNetV2, and YOLOv5.
- Classification and computational characteristics are jointly evaluated using Accuracy, Precision, Recall, F1-score, AUC, parameter count, computational complexity, model size, inference latency, and inference throughput. Statistical analysis is performed using matched fold-wise observations within the five-fold cross-validation framework.
- Model interpretability and transferability are further investigated through Grad-CAM visualization and cross-dataset evaluation using the 10 classes shared by the PlantVillage and Kaggle datasets, providing complementary evidence regarding the spatial basis and transferability of the model’s predictions.

The remainder of this paper is organized as follows. Section 2 reviews recent developments in tomato leaf disease classification and lightweight deep learning techniques. Section 3 describes the datasets, proposed methodology, network architecture, data-partitioning strategy, and experimental settings. Section 4 presents the experimental results, including independent test-set evaluation, five-fold cross-validation and statistical analysis, computational assessment, ablation analysis, ROC analysis, cross-dataset evaluation, and Grad-CAM-based interpretability analysis. Finally, Section 5 summarizes the main findings, limitations, and future research directions.

## 2 Related work

Deep learning has significantly advanced automated plant disease classification by improving feature learning, classification accuracy, and real-time inference. Recent research has focused on CNNs, YOLO-based architectures, attention mechanisms, and lightweight models to support precision agriculture. This section reviews representative studies on tomato leaf disease classification, identifies their strengths and limitations, and highlights the research gap addressed by the proposed edge-oriented YOLOv8n-CLS framework.

### 2.1 Deep learning approaches for tomato leaf disease classification

Deep learning has become the predominant approach for tomato leaf disease classification because it can automatically learn discriminative features from leaf images without relying on handcrafted descriptors (Agarwal et al., 2020; Thangaraj et al., 2022; Seth et al., 2023). Early CNN-based models demonstrated the feasibility of automated disease recognition and achieved encouraging classification performance under controlled conditions (Agarwal et al., 2020; Paymode et al., 2021). Subsequently, transfer learning approaches further improved predictive accuracy by utilizing pretrained models, reducing training time and improving generalization on limited agricultural datasets (Sanida et al., 2023; Bakır, 2024).

Recent studies have explored more advanced architectures to enhance feature representation and classification robustness. Multimodal learning, ensemble strategies, and hybrid CNN-transformer networks have shown improved performance by combining complementary visual features and capturing both local and global contextual information (Chen et al., 2024; Javidan et al., 2024; Khan et al., 2024; Thangaraj et al., 2024). These approaches have demonstrated strong classification capability across multiple tomato diseases and varying imaging conditions.

Despite these advances, many high-performing models rely on complex network architectures that increase computational cost and inference time, making deployment on mobile and embedded agricultural devices challenging (Chen et al., 2024; Javidan et al., 2024; Khan et al., 2024). In addition, many studies primarily emphasize classification accuracy while providing limited evaluation of computational efficiency and deployment feasibility (Thangaraj et al., 2022; Seth et al., 2023; Le et al., 2024). Therefore, developing lightweight classification models that maintain high predictive performance while reducing computational complexity remains an important research direction.

### 2.2 YOLO-based frameworks for plant disease analysis

The YOLO (You Only Look Once) family has become one of the most widely adopted deep learning frameworks for agricultural vision because it provides an effective balance between prediction accuracy and inference speed. Recent studies have improved YOLO architectures through enhanced feature extraction, feature fusion, and lightweight network designs for tomato leaf disease analysis (Bellout et al., 2025; Chen et al., 2025; Liu et al., 2025; Ramos and Sappa, 2025; Shen et al., 2025; Wang et al., 2025; Yan and Li, 2025). Lightweight variants such as LT-YOLOv10n further demonstrate the potential of YOLO models for real-time image-based disease screening on IoT and edge-based agricultural platforms (Bellout et al., 2025), while comparative studies confirm the strong performance of modern YOLO architectures across diverse plant disease datasets (Ramos and Sappa, 2025).

Although these methods achieve promising results, most formulate disease classification as an object detection task that requires bounding-box annotations during training (Bellout et al., 2025; Chen et al., 2025; Liu et al., 2025; Shen et al., 2025; Wang et al., 2025). However, widely used tomato disease datasets, including the PlantVillage Tomato Dataset and the Kaggle Tomato Leaves Dataset, provide image-level class labels rather than localization annotations (Hughes and Salathé, 2015; Motwani, 2019). Consequently, object detection frameworks may introduce unnecessary annotation effort and computational overhead for classification problems.

Recent developments in the YOLO ecosystem have introduced classification-oriented architectures that eliminate the need for object localization while retaining efficient feature extraction capabilities. Long and Zhou (2025) demonstrated the effectiveness of YOLOv8-CLS for image classification, and Zhu et al. (2025) successfully applied a classification-oriented YOLO framework to citrus leaf disease classification using image-level annotations. Nevertheless, relatively few studies have investigated lightweight YOLOv8n-CLS models for tomato leaf disease classification while jointly considering classification accuracy, computational efficiency, and deployment on resource-constrained edge devices. These limitations provide the motivation for the Attention-Enhanced YOLOv8n-CLS.

Recent advances have further explored enhanced YOLO architectures for plant disease detection. Shafique et al. (2026) proposed an enhanced YOLOv11-based framework for real-time multiclass citrus leaf disease detection, highlighting the application of advanced deep-learning architectures to agricultural disease recognition. Although their study focuses on citrus and an object-detection task, whereas the present study addresses image-level tomato leaf disease classification, it provides a relevant recent comparison in the broader field of YOLO-based plant disease analysis. These developments further emphasize the importance of balancing disease-recognition performance with computational efficiency in practical agricultural applications.

### 2.3 Attention mechanisms and lightweight feature enhancement

Attention mechanisms have become an effective strategy for improving deep learning models by enabling them to focus on the most informative image regions while suppressing irrelevant background features. In plant disease analysis, attention modules help highlight disease symptoms that may be subtle or distributed across the leaf surface, thereby improving feature representation and classification performance (Wang et al., 2024; Sun et al., 2025). Foundational attention mechanisms, including Squeeze-and-Excitation (SE) networks (Hu et al., 2018), the Convolutional Block Attention Module (CBAM) (Woo et al., 2018), and Coordinate Attention (Hou et al., 2021), have demonstrated their ability to enhance feature extraction with relatively low computational overhead and have inspired many recent agricultural vision models.

Several studies have incorporated attention mechanisms into tomato leaf disease classification. Sun et al. (2025) combined global and local feature fusion to improve disease recognition, while Wang et al. (2024) integrated attention with multi-scale feature fusion to enhance discriminative learning. Shen et al. (2025) further improved YOLO-based disease detection using attention-guided feature optimization, and Chen et al. (2024) demonstrated that hybrid CNN-transformer architectures can effectively capture both local disease characteristics and global contextual information. More recently, attention-based architectures have continued to improve classification accuracy by strengthening disease-specific feature representation (Dhiman et al., 2023; Shafique et al., 2026).

Although attention mechanisms consistently improve predictive performance, many existing approaches introduce additional network complexity and computational cost. Such increases may limit their applicability to edge devices where memory, processing power, and inference speed are constrained. Therefore, lightweight attention strategies that enhance feature representation while maintaining computational efficiency remain an important research direction for practical agricultural applications.

### 2.4 Lightweight deep learning models for edge-based agriculture

The increasing adoption of smartphones, unmanned aerial vehicles, and embedded sensing platforms has accelerated the demand for lightweight deep learning models capable of performing real-time image-based disease screening directly on edge devices. Compared with cloud-based systems, edge computing reduces communication latency, improves data privacy, and enables continuous monitoring in agricultural environments where internet connectivity may be limited (Bellout et al., 2025; Wei et al., 2026).

Recent studies have proposed lightweight architectures to reduce computational complexity while maintaining satisfactory classification performance. Bellout et al. (2025) developed LT-YOLOv10n for IoT-enabled disease monitoring, whereas Le et al. (2024) introduced a lightweight hybrid CNN framework for sustainable agricultural applications. Mobile-friendly architectures have also demonstrated promising results for plant disease recognition by balancing predictive accuracy with computational efficiency (Bakır, 2024; Shanthi et al., 2024; Rahaman et al., 2026). Comparative evaluations further indicate that model optimization and efficient feature extraction are essential for deploying deep learning models on resource-constrained platforms without substantial loss of performance (Ramos and Sappa, 2025; Ruhad et al., 2025).

Despite these advances, achieving an effective balance between classification accuracy, inference speed, and computational complexity remains challenging. Many lightweight models improve deployment efficiency but exhibit reduced predictive performance, whereas more accurate architectures often require greater computational resources. Consequently, there is a growing need for classification frameworks that simultaneously provide high accuracy, efficient inference, and suitability for potential for near-real-time applications in precision agriculture.

### 2.5 Research gap and motivation

The existing literature demonstrates that deep learning has substantially improved automated tomato leaf disease classification through advances in CNNs, YOLO-based models, lightweight architectures, and attention mechanisms (Thangaraj et al., 2022; Seth et al., 2023). Nevertheless, several practical challenges remain unresolved. First, most YOLO-based studies formulate disease recognition as an object detection task, requiring bounding-box annotations that are unavailable in commonly used tomato disease datasets such as PlantVillage and the Kaggle Tomato Leaves Dataset, which primarily provide image-level class labels (Hughes and Salathé, 2015; Motwani, 2019). Second, many recent models achieve high classification accuracy by incorporating increasingly complex architectures or attention modules, resulting in greater computational cost and reduced suitability for resource-constrained edge devices (Dhiman et al., 2023; Wang et al., 2024; Bellout et al., 2025; Sun et al., 2025). Third, existing studies primarily emphasize predictive accuracy, while comparatively limited attention has been given to computational efficiency, inference speed, model complexity, cross-dataset robustness, and deployment feasibility under a unified evaluation framework (Ramos and Sappa, 2025; Ruhad et al., 2025).

Motivated by these limitations, this study investigates an edge-oriented enhancement of the YOLOv8n-CLS architecture for image-level tomato leaf disease classification. Instead of introducing a fundamentally new network architecture, the Attention-Enhanced YOLOv8n-CLS integrates a lightweight spatial attention mechanism into the YOLOv8n-CLS classifier to improve disease-relevant feature representation while maintaining computational efficiency. The framework is designed to operate directly on image-level annotations, reducing annotation effort while supporting real-time inference on resource-constrained devices. In addition to classification performance, this study provides a comprehensive evaluation that considers computational complexity, inference speed, ablation analysis, cross-dataset validation, and Grad-CAM-based model interpretability to assess the framework’s practical applicability for intelligent crop monitoring.

Recent developments in enhanced YOLO architectures, including the YOLOv11-based citrus leaf disease detection framework proposed by Shafique et al. (2026), demonstrate the continued application of advanced YOLO models to agricultural disease recognition. Although such studies provide relevant insights into architectural enhancement and computational efficiency, differences in crop type, task formulation, and dataset characteristics limit direct comparison with the present study. Therefore, the current work focuses on image-level tomato leaf disease classification using YOLOv8n-CLS with lightweight spatial attention, while jointly evaluating classification performance, computational complexity, cross-dataset transferability, and model interpretability.

**Table 1** shows that existing studies have made significant progress in tomato leaf disease classification using CNNs, YOLO-based frameworks, and attention mechanisms. However, most approaches either depend on bounding-box annotations or emphasize classification accuracy without adequately considering computational efficiency and practical deployment. These observations highlight the need for lightweight image-level classification frameworks that provide a better balance between predictive performance, inference speed, and deployment feasibility for real-world precision agriculture, which forms the focus of the proposed study.

**Table 1:** Comparative analysis of representative deep learning approaches for tomato leaf disease classification.

Study	Method	Annotation	Attention	Edge-oriented	Main strength	Main limitation
(Chen et al., 2025)	Improved YOLOv8n	Bounding boxes	No	No	High detection accuracy	Requires object localization
(Sun et al., 2025)	Global–Local Feature Fusion	Image labels	Yes	Limited	Improved feature representation	Higher computational cost
(Long and Zhou, 2025)	YOLOv8-CLS	Image labels	No	Yes	Efficient image classification	Limited agricultural validation
(Zhu et al., 2025)	YOLOv8-CLS	Image labels	Yes	Limited	High classification accuracy	Evaluated on citrus dataset
(Bellout et al., 2025)	LT-YOLOv10n	Bounding boxes	No	Yes	Lightweight IoT deployment	Detection-based framework
(Wang et al., 2025)	BED-YOLO	Bounding boxes	Yes	Limited	Enhanced feature fusion	Increased model complexity
(Liu et al., 2025)	YOLO-BSMamba	Bounding boxes	Yes	Limited	Robustness in complex backgrounds	High computational cost
(Khan et al., 2024)	Multimodal Deep Learning	Image labels	Yes	No	Strong feature learning	Complex architecture
(Javidan et al., 2024)	Ensemble Learning	Image labels	No	No	Improved robustness	Increased computational complexity
(Le et al., 2024)	Lightweight CNN	Image labels	No	Yes	Efficient inference	Moderate classification accuracy
(Wang et al., 2024)	Multi-scale Attention	Image labels	Yes	Limited	Enhanced feature extraction	Additional computational overhead
This Study	YOLOv8n-CLS + Lightweight Spatial Attention	Image labels	Yes	Yes	Balanced accuracy, computational efficiency, and real-time edge deployment with comprehensive evaluation	Performance depends on image-level disease visibility

## 3 Materials and methods

This section describes the methodology adopted for tomato leaf disease classification using the proposed Attention-Enhanced YOLOv8n-CLS. The overall workflow includes image preprocessing, feature extraction using the YOLOv8n-CLS backbone, lightweight spatial attention for feature refinement, and disease classification through a fully connected prediction layer. The Attention-Enhanced YOLOv8n-CLS is designed to operate directly on image-level annotations, eliminating the need for bounding-box localization while maintaining computational efficiency for potential for near-real-time applications on resource-constrained edge devices (Abdullah et al., 2024; Islam et al., 2024).

To comprehensively evaluate the Attention-Enhanced YOLOv8n-CLS, experiments were conducted using two publicly available benchmark datasets: the PlantVillage Tomato Dataset and the Kaggle Tomato Leaf Disease Dataset (Hughes and Salathé, 2015; Motwani, 2019). A standardized preprocessing pipeline, including image normalization, data augmentation, stratified dataset partitioning, and class imbalance handling, was applied before model training. The model was trained using supervised learning and evaluated using classification performance metrics together with deployment-oriented measures such as inference speed, computational complexity, and model size (Le et al., 2024; Thangaraj et al., 2024). The overall methodology is illustrated in **Figure 1**, while the complete processing pipeline is summarized in **Algorithm 1**.

**Figure 1:**
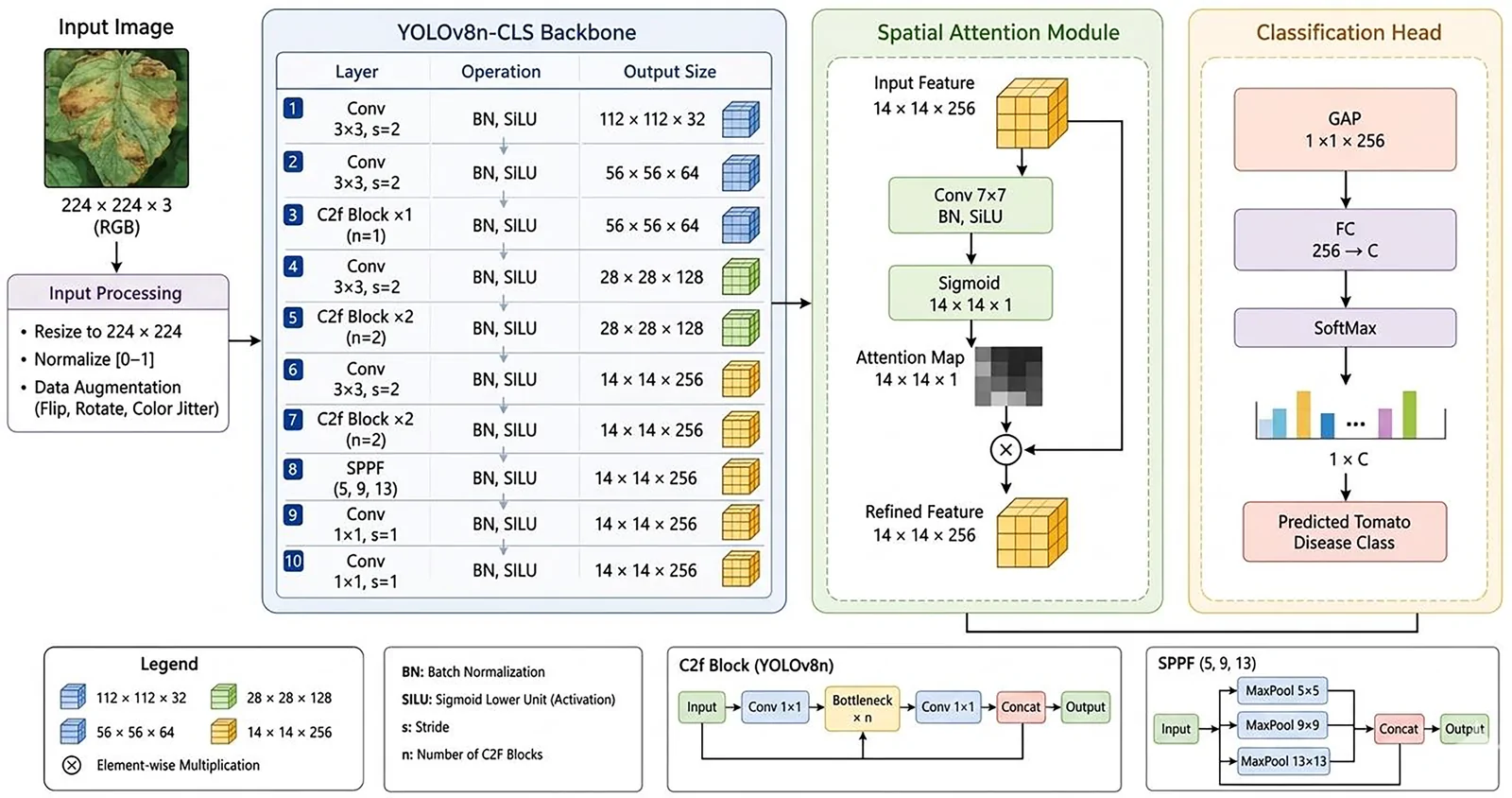
Proposed attention-enhanced YOLOv8n-CLS for tomato leaf disease classification.

Algorithm 1

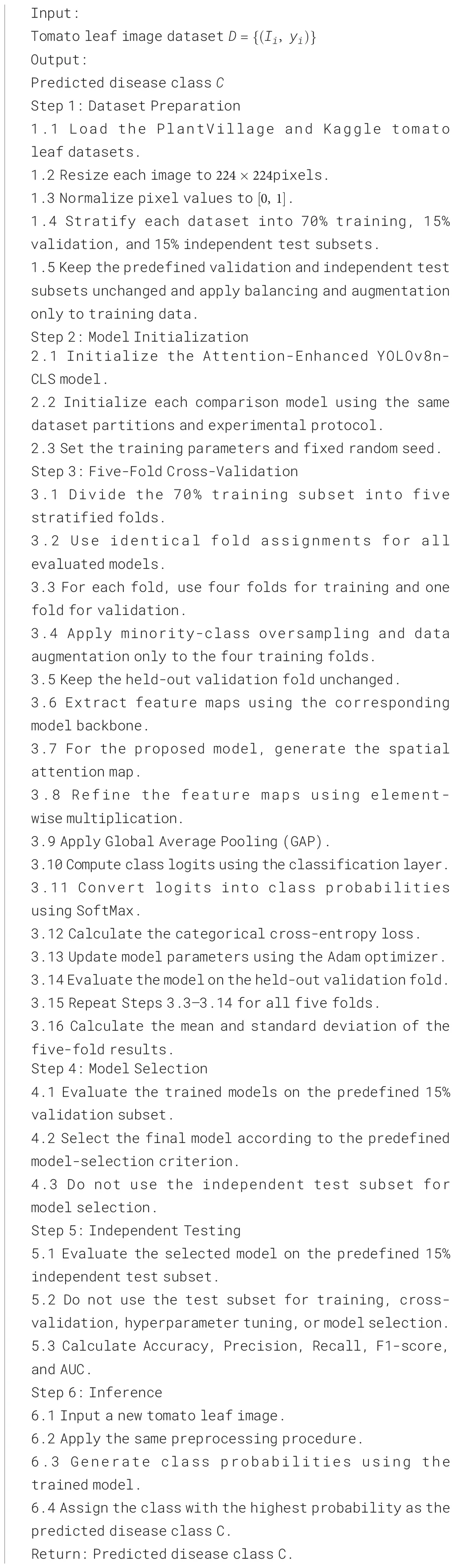

### 3.1 Overall framework

**Figure 1** presents the overall architecture of the proposed Attention-Enhanced YOLOv8n-CLS. The framework is based on the lightweight YOLOv8n-CLS classification architecture and incorporates a spatial attention module to improve the representation of disease-relevant features while preserving computational efficiency. Unlike object detection models, the Attention-Enhanced YOLOv8n-CLS performs image-level classification and therefore does not require bounding-box annotations, making it well suited for tomato leaf disease datasets that provide only image-level labels (Hughes and Salathé, 2015; Motwani, 2019; Long and Zhou, 2025; Zhu et al., 2025).

The workflow begins by resizing and normalizing the input tomato leaf images before they are passed to the YOLOv8n-CLS backbone for hierarchical feature extraction. The extracted feature maps are subsequently refined using a lightweight spatial attention module, which enhances regions associated with visible disease symptoms while reducing the influence of irrelevant background information. The refined feature representations are then aggregated through Global Average Pooling (GAP) and forwarded to a fully connected classification layer. Finally, the SoftMax function generates the probability distribution over all disease categories, and the class with the highest probability is selected as the final prediction (Paymode et al., 2021; Shen et al., 2025).

The overall design emphasizes a balance between classification accuracy and computational efficiency. By combining the lightweight YOLOv8n-CLS backbone with an attention-based feature refinement strategy, the Attention-Enhanced YOLOv8n-CLS aims to improve disease discrimination while maintaining the low computational cost required for deployment on edge devices used in precision agriculture (Le et al., 2024; Wang et al., 2024; Bellout et al., 2025; Rahaman et al., 2026; Wei et al., 2026).

### 3.2 Problem formulation

This study formulates tomato leaf disease recognition as an image-level classification task that learns visual symptom patterns associated with the disease categories provided by the source datasets. This task is distinct from laboratory-based pathogen diagnosis because the proposed model does not independently confirm the causal pathogen or establish the biological cause of the observed symptoms from an input image. Accordingly, the task is defined as image-based symptom classification and preliminary disease screening, rather than definitive pathogen diagnosis.

The present study adopts a single-label classification formulation in which each input image is associated with one dataset-provided disease category. The available dataset annotations do not provide explicit multi-label designations or independently verified mixed-infection status for individual images. Therefore, the presence or absence of simultaneous infections cannot be established from the dataset labels alone. the proposed framework is therefore designed to classify the single dataset-provided category associated with each image and is not intended to explicitly identify multiple co-occurring diseases within the same leaf or plant.

For viral disease categories, including Tomato Yellow Leaf Curl Virus and Tomato Mosaic Virus, the model should likewise be interpreted as classifying visual patterns associated with the corresponding dataset labels rather than confirming viral infection. Similar symptoms may arise from other pathogens, physiological disorders, nutritional deficiencies, or environmental stresses. Predictions for these categories should therefore be regarded as symptom-based classification or preliminary screening indications rather than definitive viral identification.

Unlike object detection methods, the Attention-Enhanced YOLOv8n-CLS operates directly on image-level annotations and therefore does not require bounding-box localization. This formulation is consistent with the annotation format of the PlantVillage Tomato Dataset and the Kaggle Tomato Leaf Disease Dataset (Hughes and Salathé, 2015; Motwani, 2019). The training dataset is mathematically represented by Equation (1).

(1) $$D={\left\{(I_{i}\text{, }y_{i})\right\}}_{i=1}^{N}$$

where $I_{i}\in {\mathbb{R}}^{H\times W\times 3}\;$denotes the *i*-th RGB image, $y_{i}\in \{1\text{, }2\text{, }\ldots \text{, }C\}$ denotes its corresponding dataset-provided class label, *C* represents the total number of classes, and *N* denotes the total number of training samples. The objective is to learn a mapping function (Equation 2):

(2) $$f_{\theta }:I\to \hat{y}$$

where $f_{\theta }\;$denotes the Attention-Enhanced YOLOv8n-CLS model with learnable parameters θ, and $\hat{y}\;$represents the predicted class. The probability distribution over the *C* classes is computed using the Softmax function, as defined in Equation 3 (Thangaraj et al., 2022):

(3) $${\hat{p}}_{i}=\frac{\text{exp}(z_{i})}{\sum_{j=1}^{C}\text{exp}(z_{j})}$$

where $z_{i}\;$denotes the logit corresponding to class *i*, and ${\hat{p}}_{i}\;$represents the predicted probability for that class. The categorical cross-entropy loss is used for model optimization, as given in Equation 4.

(4) $$L_{\text{cls}}=-\sum_{i=1}^{C}y_{i}\text{log}({\hat{p}}_{i})$$

where $y_{i}$ denotes the ground-truth class indicator and ${\hat{p}}_{i}$denotes the predicted probability for class *i*. This image-level formulation eliminates the need for bounding-box annotations and localization processing, thereby reducing annotation requirements and computational overhead while enabling efficient image classification (Chen et al., 2024; Wang et al., 2024). The observed computational performance suggests potential for near-real-time applications on resource-constrained platforms. However, the model was not evaluated on actual agricultural edge devices, and therefore its real-world edge deployment performance remains to be validated.

### 3.3 Proposed attention-enhanced YOLOv8n-CLS

**Figure 1** illustrates the proposed Attention-Enhanced YOLOv8n-CLS. The proposed model is built upon the lightweight YOLOv8n-CLS classification architecture and incorporates a lightweight spatial attention module to improve disease-specific feature representation while maintaining computational efficiency. Unlike conventional YOLO-based object detection models, the framework performs image-level classification and therefore operates directly on RGB images without requiring bounding-box annotations (Long and Zhou, 2025; Zhu et al., 2025).

The framework consists of four sequential components: (i) input processing, (ii) feature extraction using the YOLOv8n-CLS backbone, (iii) spatial attention-based feature refinement, and (iv) disease classification. Initially, tomato leaf images are resized and normalized before being passed to the backbone network. Hierarchical feature maps extracted by the backbone are subsequently refined using a lightweight spatial attention module that emphasizes disease-affected regions while suppressing irrelevant background information. The refined feature maps are aggregated using Global Average Pooling (GAP) and finally passed to a fully connected classification layer, where the SoftMax function produces the probability of each disease category (Liu et al., 2023).

The lightweight architecture is designed to balance predictive accuracy with computational efficiency, making it suitable for deployment on resource-constrained agricultural devices. The detailed implementation of each component is described in the following subsections. As illustrated in **Figure 1** and summarized in **Table 2**, the proposed Attention-Enhanced YOLOv8n-CLS consists of five main stages: input preprocessing, feature extraction using the YOLOv8n-CLS backbone, lightweight spatial attention, Global Average Pooling (GAP), and classification through a fully connected layer with SoftMax activation. This lightweight design enables efficient and accurate tomato leaf disease classification for real-time edge deployment.

**Table 2:** Architecture summary of the proposed attention-enhanced YOLOv8n-CLS.

Stage	Operation	Output
Input	Resize and normalize RGB image (224 × 224 × 3)	Normalized image
Backbone	YOLOv8n-CLS feature extraction	Hierarchical feature maps
Attention Module	Lightweight spatial attention	Refined feature maps
Feature Aggregation	Global Average Pooling	Feature vector
Classification Head	Fully connected layer + SoftMax	Disease class probabilities

#### 3.3.1 Input processing

The input stage prepares tomato leaf images for feature extraction by standardizing their dimensions and pixel values. Each RGB image is resized to 224 × 224 × 3, which corresponds to the input size of the YOLOv8n-CLS architecture and provides an effective balance between computational efficiency and feature preservation (Khan et al., 2024; Long and Zhou, 2025). Pixel intensities are then normalized to the range (0, 1) to improve numerical stability and facilitate faster model convergence during training. The input image is mathematically represented by Equation 5.

(5) $$I\in {\mathbb{R}}^{224\times 224\times 3}$$

Where *I* denotes the normalized RGB image. The processed image is subsequently forwarded to the backbone network for hierarchical feature extraction.

#### 3.3.2 YOLOv8n-CLS backbone

The backbone network is responsible for extracting hierarchical feature representations from the pre-processed tomato leaf images. The Attention-Enhanced YOLOv8n-CLS adopts the lightweight YOLOv8n-CLS backbone, which comprises convolutional layers, C2f modules, Batch Normalization (BN), and the SiLU activation function (Javidan et al., 2024; Sreedevi and Manike, 2024; Long and Zhou, 2025). These components progressively capture low-level texture features and high-level semantic information while maintaining a compact model suitable for edge deployment. The output feature representation is mathematically defined by Equation 6.

(6) $$F=\phi (I;\theta_{b})$$

Where *F* denotes the extracted feature maps, ϕ(·) represents the backbone transformation, and $\theta_{b}$denotes the learnable backbone parameters.

As summarized in **Table 2**, the backbone consists of successive convolutional and C2f blocks that gradually reduce the spatial resolution while increasing the feature depth. After feature extraction, the output feature maps are forwarded to the lightweight spatial attention module for further refinement before classification.

#### 3.3.3 Lightweight spatial attention module

To improve the representation of disease-specific features, a lightweight spatial attention module is integrated after the YOLOv8n-CLS backbone. The module enhances regions associated with disease symptoms, such as lesions, discoloration, and necrotic spots, while suppressing irrelevant background information (Shanthi et al., 2024). Compared with complex attention mechanisms, the proposed module introduces minimal computational overhead, making it suitable for real-time edge deployment (Hu et al., 2018; Woo et al., 2018; Hou et al., 2021). Given the backbone feature map F, the spatial attention map is computed using Equation 7.

(7) A=σ(Conv(F))

Where *A* denotes the attention map, Conv(·)represents the convolution operation, and σ(·) is the sigmoid activation function. The refined feature map is then obtained through element-wise multiplication, as defined in Equation 8.

(8) $$F^{\prime }=A⊙F$$

Where ⊙ denotes element-wise multiplication. The refined feature map $F^{\prime }$ is subsequently forwarded to the classification head for disease prediction.

#### 3.3.4 Classification head

The classification head converts the refined feature representation into disease class probabilities. First, Global Average Pooling (GAP) aggregates spatial information into a compact feature vector, thereby reducing model complexity while preserving discriminative features (Kaur et al., 2022; Bachhal et al., 2024; Bakır, 2024). The pooled feature vector is then passed through a fully connected layer to generate the output logits, as defined in Equation 9.

(9) z=(Wɡ+b)

Where *ɡ* is the pooled feature vector, *W* and *b* are the trainable parameters, and *z* denotes the output logits. The SoftMax function converts the logits into class probabilities, and the disease category with the highest probability is selected as the final prediction (Priyadharshini and Dolly, 2023).

#### 3.3.5 Training objective

The Attention-Enhanced YOLOv8n-CLS is trained using supervised learning to minimize the discrepancy between predicted and ground-truth disease labels (Kumar et al., 2024). Model optimization is performed using the categorical cross-entropy loss, which is widely adopted for multi-class image classification, as defined in Equation 10.

(10) $$L_{\text{cls}}=-\sum_{i=1}^{C}y_{i}\text{log}({\hat{y}}_{i})$$

Where $y_{i}\;$denotes the ground-truth class label, ${\hat{y}}_{i}\;$represents the predicted probability for class *i*, and *C* is the total number of disease categories. During optimization, the model parameters are updated using the Adam optimizer to improve convergence and classification performance.

### 3.4 Workflow of the attention-enhanced YOLOv8n-CLS

The overall workflow of the proposed Attention-Enhanced YOLOv8n-CLS is presented in **Figure 2**, while the corresponding computational procedure is summarized in **Algorithm 1**. The framework begins with image preprocessing, where tomato leaf images are resized to (224 × 224) pixels and normalized before being partitioned into training, validation, and testing subsets. Data augmentation is applied exclusively to the training set to improve model generalization and prevent overfitting (Ayyad et al., 2025).

**Figure 2:**
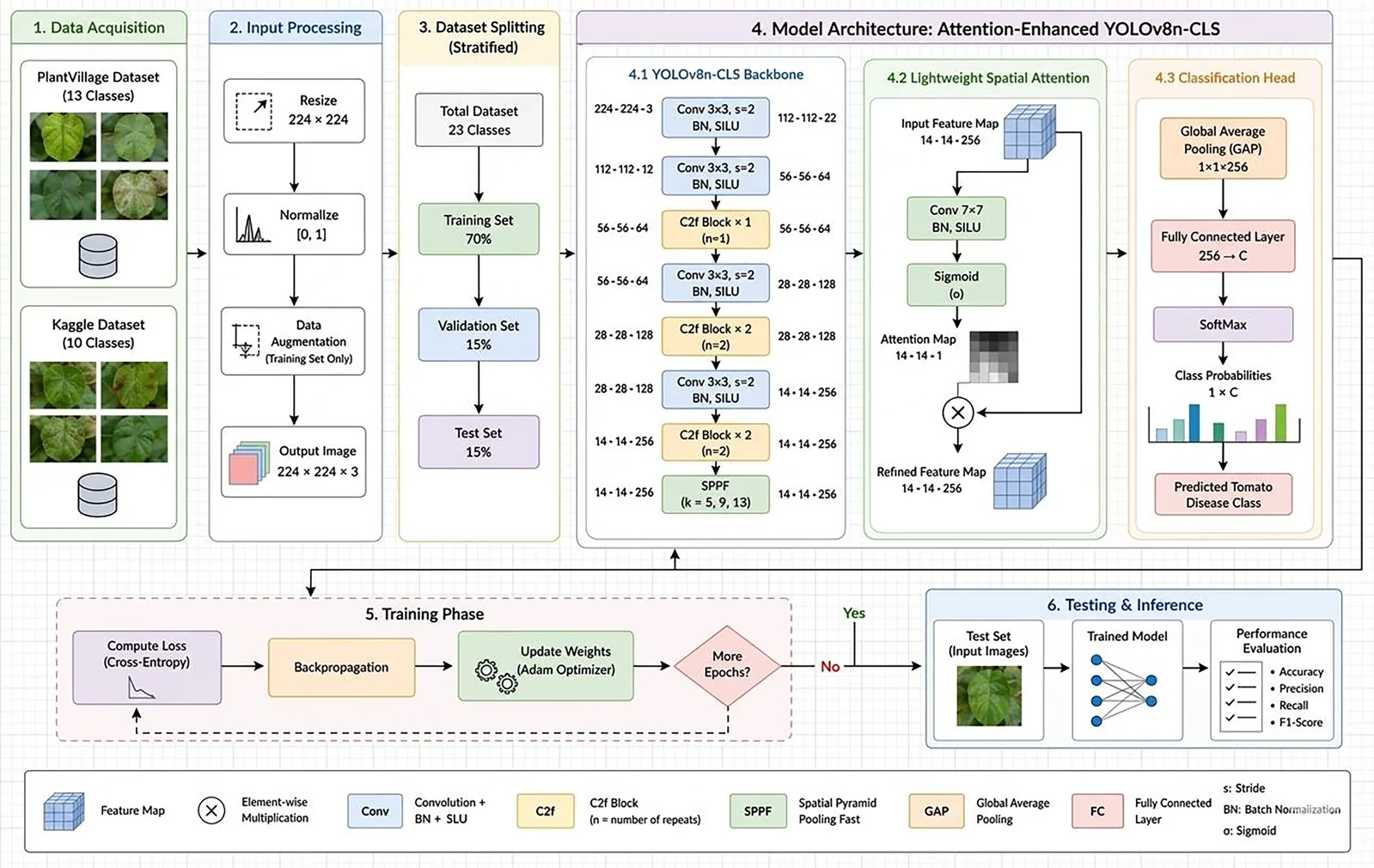
Workflow of the proposed attention-enhanced YOLOv8n-CLS for tomato leaf disease classification.

The pre-processed images are then forwarded to the YOLOv8n-CLS backbone for hierarchical feature extraction. The extracted feature maps are subsequently refined using a lightweight spatial attention module, which emphasizes disease-relevant regions while suppressing background information. The refined features are aggregated through Global Average Pooling (GAP) and passed to a fully connected classification layer. Finally, the SoftMax function computes the probability of each disease category, and the class with the highest probability is selected as the final prediction (Ruhad et al., 2025; Wei et al., 2026).

During training, model parameters are optimized using the Adam optimizer with the categorical cross-entropy loss function. Model performance is continuously monitored using the validation dataset, and the model achieving the highest validation accuracy is retained for final evaluation on the independent test set. This workflow provides an effective balance between classification accuracy, computational efficiency, and deployment suitability for real-time agricultural applications (Rahman et al., 2023).

The independent test subset was excluded from all model-development procedures and was not included in the five-fold cross-validation analysis. Five-fold cross-validation was performed as a separate evaluation procedure to characterize performance variability across matched data partitions (Tang et al., 2023). Therefore, the independent test-set results and the five-fold cross-validation results represent distinct analyses and are reported separately in Sections 4.2 and 4.3, respectively.

The overall workflow of the proposed Attention-Enhanced YOLOv8n-CLS is illustrated in **Figure 2**, while the corresponding training and inference procedure is presented in Algorithm 1. The workflow describes the complete pipeline from data preprocessing and feature extraction to model optimization and disease classification, providing a clear overview of the proposed methodology and ensuring reproducibility of the experimental framework.

### 3.5 Datasets

The Attention-Enhanced YOLOv8n-CLS was evaluated on two publicly available benchmark datasets: the PlantVillage Tomato Dataset (Hughes and Salathé, 2015) and the Kaggle Tomato Leaf Disease Dataset (Motwani, 2019). Both contain RGB images of healthy and symptomatic tomato leaves and provide complementary image-acquisition characteristics for evaluating classification performance.

The PlantVillage Tomato Dataset contains 14, 529 images across 10 classes, including nine disease categories and one healthy class (Hughes and Salathé, 2015). Its images are relatively standardized in terms of background and illumination. The Kaggle Tomato Leaf Disease Dataset contains 20, 680 images across 11 classes, including ten disease categories and one healthy class (Motwani, 2019), with greater variation in illumination, background, leaf orientation, and overall appearance. However, these datasets should not be considered fully representative of naturally occurring field conditions.

The dataset-provided labels were used as target classes without independent laboratory, molecular, serological, or microbiological confirmation. Thus, the results represent image-level recognition of dataset-defined categories rather than definitive pathogen diagnosis. This is particularly relevant to viral categories, where visually similar symptoms may result from other pathogens, physiological disorders, or environmental stresses. The single-label annotations also do not establish the presence or absence of mixed infections; therefore, performance under co-infection conditions remains untested (Kansal et al., 2024).

The two datasets share 10 classes: Bacterial Spot, Early Blight, Late Blight, Leaf Mold, Septoria Leaf Spot, Spider Mites, Target Spot, Tomato Yellow Leaf Curl Virus, Tomato Mosaic Virus, and Healthy. Powdery Mildew is present only in the Kaggle dataset and was therefore excluded from the cross-dataset experiments in Section 4.8. The same 10-class label space was used for both PlantVillage → Kaggle and Kaggle → PlantVillage evaluation.

Because the available data are provided as individual images with single class labels and do not consistently identify the source plant or leaf, the experiments were conducted at the image level rather than the plant level. A summary of the datasets is provided in **Table 3**, with class distributions shown in **Figures 3** and **4**.

**Table 3:** Overview of the tomato leaf disease datasets.

Dataset	Number of classes	Total images	Data characteristics
PlantVillage Tomato (Hughes and Salathé, 2015)	10 (9 Diseases + 1 Healthy)	14, 529	Controlled environment
Kaggle Tomato Leaf Disease (Motwani, 2019)	11 (10 Diseases + 1 Healthy)	20, 680	Diverse environmental conditions

**Figure 3:**
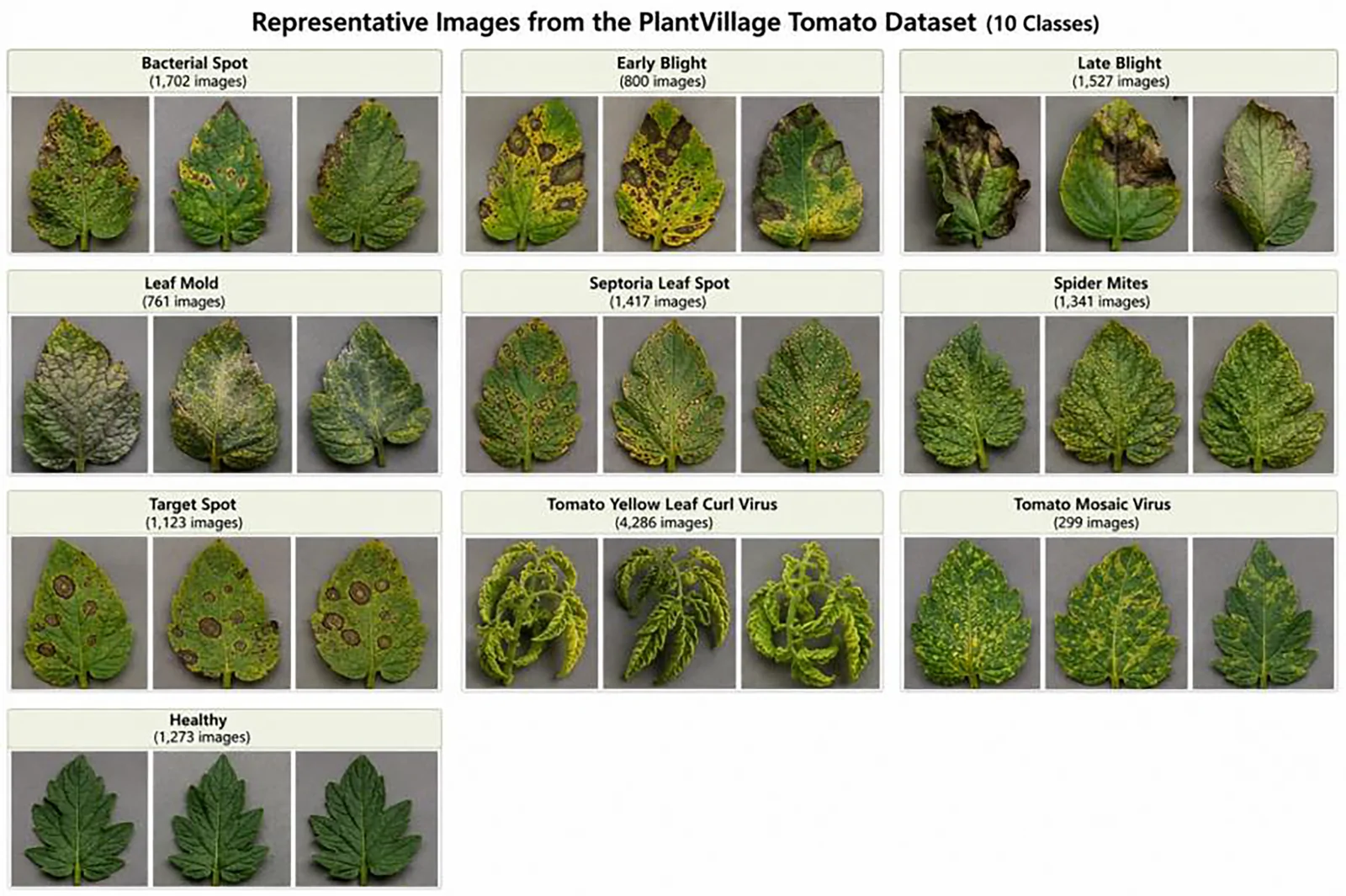
Representative tomato leaf images from the ten classes used in this study, including nine disease categories (bacterial spot, early blight, late blight, leaf mold, septoria leaf spot, spider mites, target spot, tomato yellow leaf curl virus, and tomato mosaic virus) and one healthy class. The images were acquired under controlled laboratory conditions with uniform backgrounds, providing a standardized benchmark for tomato leaf disease classification.

**Figure 4:**
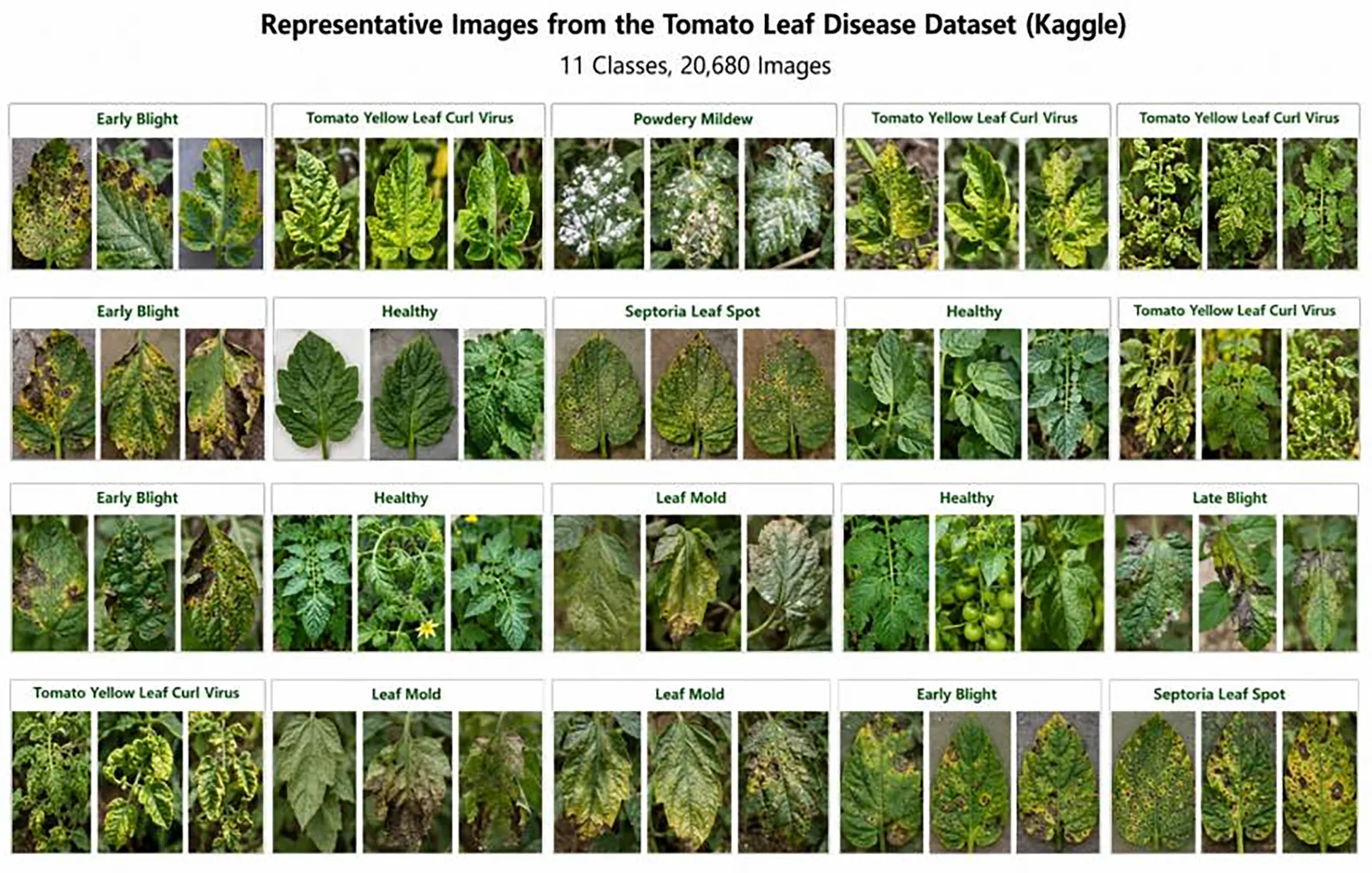
Representative images from the Kaggle Tomato Leaf Disease Dataset, illustrating the eleven classes used in this study, including the additional Powdery Mildew category. The images were collected under diverse environmental conditions with variations in lighting, background complexity, and disease appearance, reflecting more realistic field scenarios.

Representative images of the disease classes from the PlantVillage and Kaggle tomato leaf datasets are presented in **Figures 3** and **4**, respectively. The PlantVillage dataset comprises images acquired under controlled laboratory conditions, whereas the Kaggle dataset exhibits greater variability in background, illumination, and disease manifestation, providing a more challenging benchmark for evaluating model robustness.

#### 3.5.1 Class distribution and imbalance

The class distributions of the PlantVillage and Kaggle datasets are presented in **Figures 3** and **4**, respectively, while the number of samples in each disease category is summarized in **Table 4**. Both datasets exhibit noticeable class imbalance, with substantial differences in the number of images across disease categories. For example, the PlantVillage dataset contains 4, 286 images of Tomato Yellow Leaf Curl Virus but only 299 images of Tomato Mosaic Virus, indicating a highly uneven class distribution (Bhosale and Chhabria, 2024; Islam et al., 2024).

**Table 4:** Original class distribution of the experimental datasets.

Disease class	PlantVillage	Kaggle	Pathogen type
Bacterial Spot	1, 702	2, 261	Bacterial
Early Blight	800	1, 964	Fungal
Late Blight	1, 527	2, 490	Oomycete
Leaf Mold	761	2, 203	Fungal
Septoria Leaf Spot	1, 417	2, 305	Fungal
Spider Mites	1, 341	1, 398	Pest
Target Spot	1, 123	1, 462	Fungal
Tomato Yellow Leaf Curl Virus	4, 286	1, 631	Viral
Tomato Mosaic Virus	299	1, 722	Viral
Healthy	1, 273	2, 441	Healthy
Powdery Mildew	—	803	Fungal
Total Images	14, 529	20, 680	—

Such imbalance may bias the learning process toward majority classes and reduce classification performance for underrepresented diseases. To mitigate this issue, augmentation-based oversampling and class-weighted loss optimization were applied exclusively to the training subset, whereas the validation and testing subsets retained their original class distributions to ensure an unbiased evaluation of model performance.

#### 3.5.2 Interpretation of dataset labels

The disease-category labels provided with the PlantVillage Tomato and Kaggle Tomato Leaf Disease datasets were adopted as the target labels for the supervised image-classification experiments. The present study did not independently verify the causal pathogens using laboratory, molecular, serological, or microbiological assays. Therefore, the labels are treated as dataset-provided disease categories for visual classification rather than as pathogen diagnoses independently established by the proposed model. This distinction is particularly important for viral categories, including Tomato mosaic virus and Tomato yellow leaf curl virus, because visually similar symptoms may also arise from other pathogens, physiological disorders, nutritional deficiencies, or environmental conditions. Accordingly, the predictions generated by the proposed model represent recognition of visual patterns associated with the corresponding dataset categories and should not be interpreted as definitive confirmation of the causal pathogen.

### 3.6 Data preprocessing

A standardized preprocessing pipeline was employed to ensure data consistency and improve model generalization. The pipeline consisted of image resizing, normalization, data augmentation, and dataset partitioning. To prevent data leakage, dataset partitioning was performed before any augmentation or class-balancing operations, and all image transformation techniques were applied exclusively to the training subset (Umar et al., 2024).

#### 3.6.1 Image resizing and normalization

All input images were resized to 224 × 224 × 3 pixels to satisfy the input requirements of the YOLOv8n-CLS architecture (Umar et al., 2024; Long and Zhou, 2025). Pixel intensities were normalized to the range [0, 1] to improve numerical stability and facilitate faster convergence during model optimization. The normalization process is mathematically expressed in Equation 11.

(11) $$I_{n}=\frac{I_{r}}{255}$$

where $I_{r}\;$denotes the resized input image and $I_{n}$ represents the normalized image.

#### 3.6.2 Data augmentation

To improve robustness against variations encountered in practical agricultural environments and reduce overfitting, data augmentation was applied only to the training subset. The augmentation strategy introduced random geometric and photometric transformations, enabling the model to learn invariant features under different imaging conditions (Abdullah et al., 2024; Kaur et al., 2024). The augmentation techniques employed in this study are summarized in **Table 5**.

**Table 5:** Data augmentation strategy.

Augmentation technique	Configuration
Random Rotation	± 30°
Horizontal Flip	Probability = 0.5
Vertical Flip	Probability = 0.5
Zoom Range	0.8–1.2
Width Shift	± 20%
Height Shift	± 20%
Brightness Adjustment	0.5–1.5
Contrast Adjustment	Random intensity
Shear Transformation	± 10°

As shown in **Figure 5**, the augmentation strategy combines geometric and photometric transformations to generate diverse training samples while preserving the semantic characteristics of tomato leaf diseases. These transformations improve the model’s ability to recognize disease symptoms under different orientations, scales, illumination conditions, and viewpoints, thereby enhancing robustness and generalization when deployed in real-world agricultural environments.

**Figure 5:**
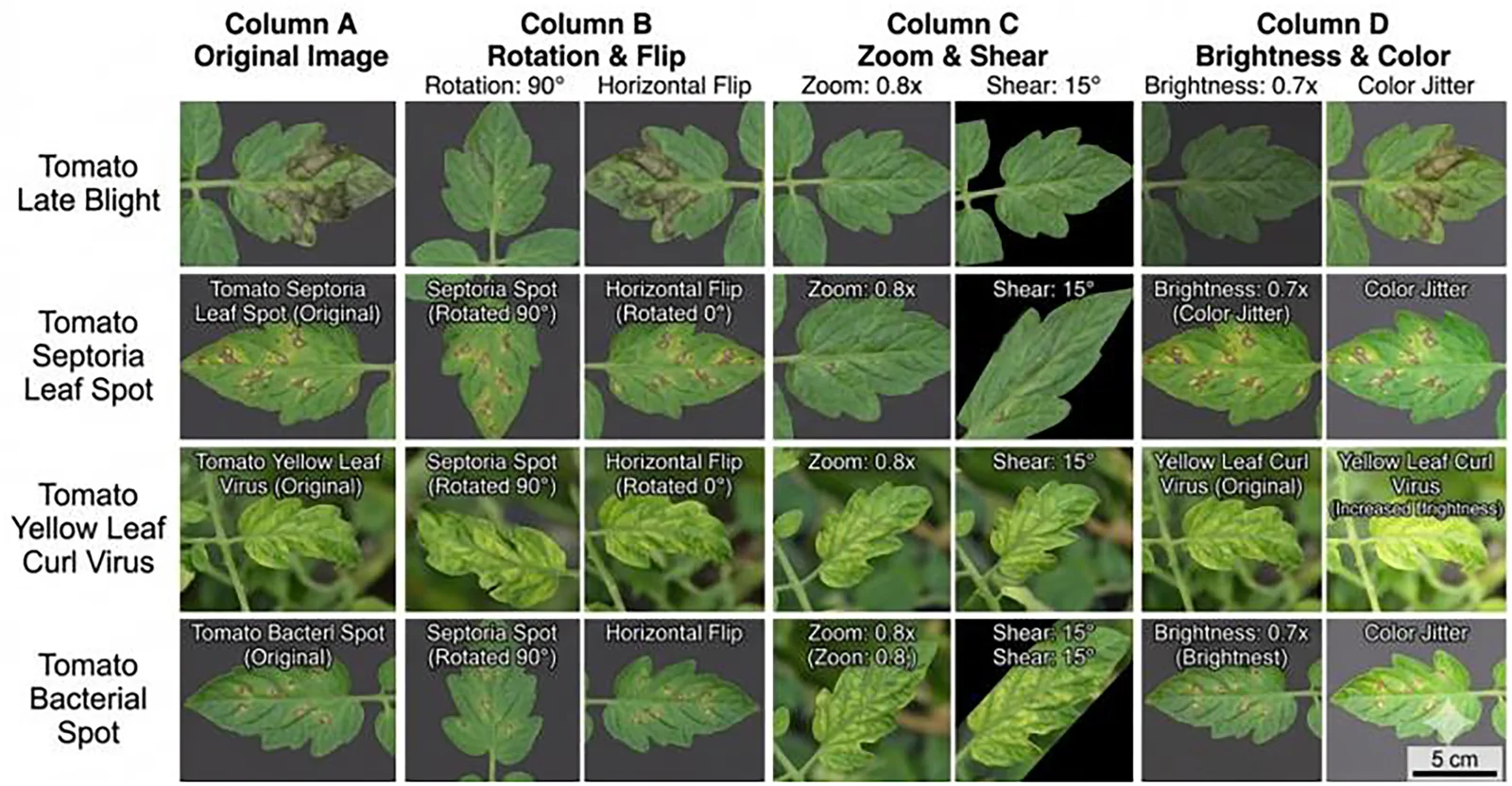
Visualization of the data augmentation techniques applied during model training.

### 3.7 Dataset partitioning and cross-validation strategy

The datasets were partitioned using stratified sampling to preserve the class distribution across subsets. Each dataset was initially divided into 70% training, 15% validation, and 15% independent test subsets. The 70% training subset was used for model development, the predefined 15% validation subset was reserved for hyperparameter tuning and model-selection decisions, and the 15% independent test subset was held out exclusively for final evaluation on previously unseen images.

Five-fold cross-validation was conducted exclusively within the 70% training subset. In each iteration, four folds were used for training and the remaining fold was used for internal validation, with each fold serving once as the validation fold. Identical fold assignments were used for all evaluated models to provide matched comparisons. Thus, the five folds were not additional partitions of the complete dataset and did not include the predefined validation or independent test subsets.

The predefined 15% validation subset remained separate from the cross-validation procedure and was used for hyperparameter tuning and model-selection decisions. The independent 15% test subset was excluded from training, cross-validation, hyperparameter tuning, and model selection and was evaluated only after model development had been completed. Accordingly, the five-fold cross-validation results and independent test-set results were treated as separate evaluations. Cross-validation results are reported as mean ± standard deviation (SD) across the five matched folds, whereas independent test-set results are reported as single final performance estimates.

To minimize information leakage, dataset partitioning was completed before data augmentation and class-balancing operations. Augmentation and oversampling were applied only to the training data available in each cross-validation iteration. The internal validation fold, predefined 15% validation subset, and independent 15% test subset were not augmented or oversampled. The same principle was followed during final model development. A fixed random seed was used for dataset partitioning and fold assignment to facilitate reproducibility.

In addition to within-dataset evaluation, a separate cross-dataset evaluation was conducted in two directions: [PlantVillage → Kaggle and Kaggle → PlantVillage]. Because the datasets contain different class sets, the cross-dataset experiments were restricted to the 10 classes shared by both datasets, with the Kaggle-specific Powdery Mildew category excluded. Model development, hyperparameter tuning, and model selection were performed exclusively using the source dataset, while the target dataset was reserved for evaluation. Target-dataset images were not used during source-domain model development. Cross-dataset results were treated as a separate generalization analysis and were not included as additional observations in the five-fold cross-validation or Friedman and Wilcoxon statistical analyses.

#### 3.7.1 Duplicate and plant-level leakage considerations

The available datasets provide image-level class labels but do not consistently provide reliable plant- or leaf-level identifiers. Therefore, the primary partitioning procedure was performed at the image level, and plant- or leaf-level grouping could not be systematically applied. Consequently, the possibility that highly similar, duplicate, near-duplicate, or images originating from the same plant or leaf may occur across different subsets cannot be completely excluded. This limitation is considered when interpreting the reported performance and generalization results. Future studies should incorporate reliable plant- or leaf-level identifiers and systematic duplicate and near-duplicate detection before dataset partitioning.

### 3.8 Experimental settings and reproducibility

All experiments were conducted using the PyTorch deep learning framework and the Ultralytics YOLOv8 implementation (Guo, 2024; Long and Zhou, 2025). The proposed Attention-Enhanced YOLOv8n-CLS and all baseline models were evaluated using the same dataset partitions and evaluation protocol. A fixed random seed was used for dataset partitioning, fold assignment, and model initialization to improve reproducibility. The principal experimental settings are summarized in **Table 6**.

**Table 6:** Experimental settings used for model training and evaluation.

Parameter	Configuration
Deep-learning framework	PyTorch
YOLO implementation	Ultralytics YOLOv8
Base architecture	YOLOv8n-CLS
Proposed architecture	Attention-Enhanced YOLOv8n-CLS
Input image size	224 × 224
Training epochs	200
Dataset split	70% training/15% validation/15% independent test
Cross-validation	Five-fold within the 70% training subset
Fold assignment	Identical across all models
Model-selection data	Predefined 15% validation subset
Independent test data	Predefined 15% test subset
Data augmentation	Applied to training data only
Class balancing	Applied to training data only
Random seed	Fixed
Computational platform	NVIDIA Tesla V100
Model-selection criterion	Minimum validation loss
Test-set usage	Final evaluation only

#### 3.8.1 Lightweight spatial attention module

A lightweight spatial attention module was integrated into the final feature-extraction stage before the classification head of YOLOv8n-CLS. The module receives a 256-channel intermediate feature representation and generates a single-channel spatial attention map using a 7×7convolution with stride 1 and padding 3. No normalization layer is applied, and a sigmoid activation is used to constrain the attention values between 0 and 1. The resulting attention map is broadcast across the channel dimension and combined with the input feature representation through element-wise multiplication (**Table 7**). The attention operation is mathematically formulated as given in Equations 12, 13.

**Table 7:** Configuration of the lightweight spatial attention module.

Parameter	Configuration
Base model	YOLOv8n-CLS
Attention type	Lightweight spatial attention
Insertion point	Final feature-extraction stage before classification head
Input channels	256
Output channels	1
Kernel size	7×7
Stride	1
Padding	3
Dilation	1
Normalization	None
Activation	Sigmoid
Attention-map size	1×H×W
Feature refinement	Element-wise multiplication
Residual connection	None
Input resolution	224×224
Additional parameters	≈0.013 M
Additional computational cost	≈0.013 GFLOPs
Total model parameters	≈3.2 M
Total model complexity	≈7.5 GFLOPs

(12) $$M=\sigma ({\text{Conv}}_{7\times 7}(F))$$

(13) $$F_{\text{out}}=F⊙M$$

where *F* represents the input feature tensor, *M* represents the spatial attention map, σ denotes the sigmoid activation function, and ⊙ denotes element-wise multiplication with broadcasting across the channel dimension.

The attention module therefore introduces a small additional computational overhead while maintaining the compact structure of the base classification architecture.

#### 3.8.2 Model training and selection

During model development, the predefined 15% validation subset was used for hyperparameter tuning and model-selection decisions. The selected model was determined according to the minimum validation loss. The independent 15% test subset remained isolated from these procedures and was used only for final evaluation. For cross-validation, the fivefold-wise observations were used to characterize performance stability and formed the paired observations for the statistical analyses described in Section 4.3.

#### 3.8.3 Computational benchmarking

Computational efficiency was evaluated using trainable parameters, FP32 weight storage, GFLOPs, inference latency, and inference throughput. All models were benchmarked on an NVIDIA Tesla V100 GPU using 224×224-pixel RGB inputs under identical inference conditions. The batch size, warm-up procedure, number of timed iterations, timing components, and numerical precision are reported in **Table 8**. Before timing, the specified warm-up iterations were performed to stabilize GPU execution, followed by the specified timed inference iterations. Average latency was calculated as the mean inference time per image, and throughput was calculated as the number of images processed per second, as defined in Equation 14.

**Table 8:** Training hyperparameter configuration.

Hyperparameter	Value
Optimizer	Adam
Learning Rate	0.001
Batch Size	32
Input Image Size	224 × 224
Loss Function	Cross-Entropy
Epochs	100
Early Stopping	Enabled
Random Seed	42

(14) $$FPS=\frac{1000}{t}$$

where *t* is the average inference time in milliseconds per image. Image loading and preprocessing were excluded from the timing measurements. FP32 weight storage was estimated from the number of trainable parameters assuming 4 bytes per parameter. The reported computational measurements therefore represent performance on the evaluated NVIDIA Tesla V100 platform and should not be interpreted as direct evidence of edge-device performance.

### 3.9 Class balancing and data augmentation

To address class imbalance, balancing was performed only on the training data. The predefined 15% validation and 15% independent test subsets were kept unchanged and were not oversampled or rebalanced. For each training set, minority-class samples were oversampled to improve class representation during model training. Data augmentation was applied only to the resulting training samples.

The same class-balancing and augmentation procedure was applied to the proposed Attention-Enhanced YOLOv8n-CLS and all five comparison models to maintain a consistent experimental protocol. No balancing or augmentation was applied to the validation or independent test subsets.

### 3.10 Hyperparameter configuration

The network was trained using the Adam optimizer with an initial learning rate of 0.001 and a batch size of 32. The categorical cross-entropy loss function was adopted for multi-class classification (Le et al., 2024; Wang et al., 2026). Early stopping and model checkpointing were employed to prevent overfitting by monitoring the validation loss during training. The primary training hyperparameters used in this study are summarized in **Table 8**.

### 3.11 Performance evaluation

The performance of the Attention-Enhanced YOLOv8n-CLS was evaluated using standard multi-class classification metrics, including accuracy, precision, recall, F1-score, and the area under the receiver operating characteristic curve (AUC) (Huang et al., 2023; Chen et al., 2024; Khan et al., 2024; Wang et al., 2024). Confusion matrices were also analyzed to examine class-wise classification performance. The evaluation metrics used to assess the classification performance are defined in Equations 15–18 as follows:

(15) $$Accuracy=\frac{TP+TN}{TP+TN+FP+FN}$$

(16) $$Precision=\frac{TP}{TP+FP}$$

(17) $$Recall=\frac{TP}{TP+FN}$$

(18) $$F1=\frac{2\times Precision\times Recall}{Precision+Recall}$$

Where TP, TN, FP, and FN denote true positives, true negatives, false positives, and false negatives, respectively. For multi-class classification, the reported precision, recall, F1-score, and AUC values were computed using macro-averaging across all disease categories.

### 3.12 Statistical analysis

Statistical analysis was designed to account for the paired structure of the five-fold cross-validation experiments. Because the same data folds were used to evaluate all six models, the corresponding fold-wise performance values were treated as paired observations rather than independent observations. Thus, the five cross-validation folds constituted the units of replication for the comparative statistical analysis.

For the overall comparison of the six models, the Friedman test was used as a non-parametric repeated-measures test because each model was evaluated using the same five folds. When a significant overall difference was detected, pairwise Wilcoxon signed-rank tests were performed to compare the proposed Attention-Enhanced YOLOv8n-CLS with each baseline model, namely ResNet50, DenseNet201, ResNet152V2, MobileNetV2, and YOLOv5. Because five pairwise comparisons were performed, the resulting *p*-values were adjusted using the Holm–Bonferroni correction to control for multiple comparisons. All statistical tests were two-sided, and statistical significance was assessed at α=0.05after adjustment for multiple comparisons.

The fold-wise results are reported to make the unit of replication and the paired comparison structure explicit. The five-fold cross-validation results represent repeated evaluations across different data partitions and were not considered five independent biological or experimental samples. Where independent training repetitions were performed, they were reported separately from the cross-validation folds, including the number of independent trainings runs and the corresponding random seeds. Performance values from the five folds are reported descriptively as mean ± standard deviation.

### 3.13 Computational complexity analysis

The computational efficiency of the Attention-Enhanced YOLOv8n-CLS was evaluated using several complementary measures, including the number of trainable parameters, floating-point operations (GFLOPs), inference speed measured in frames per second (FPS), and average inference time per image (Hu et al., 2018; Woo et al., 2018; Hou et al., 2021).

These metrics provide a comprehensive assessment of the trade-off between classification accuracy and computational cost, which is particularly important for potential for near-real-time applications on resource-constrained edge devices. Lower parameter counts and computational complexity, together with higher inference speed, indicate greater suitability for practical smart agriculture applications. The computational complexity results are presented and compared with existing state-of-the-art models in the Results section.

## 4 Experimental results and discussion

This section presents a comprehensive evaluation of the proposed Attention-Enhanced YOLOv8n-CLS for tomato leaf disease classification using the PlantVillage Tomato and Kaggle Tomato Leaf Disease datasets. The Attention-Enhanced YOLOv8n-CLS was assessed in terms of classification accuracy, robustness, computational efficiency, and deployment suitability under both controlled and diverse imaging conditions. Performance was evaluated using standard classification metrics, including Accuracy, Precision, Recall, F1-score, and the Area Under the Receiver Operating Characteristic Curve (AUC), together with deployment-oriented metrics such as inference speed (FPS), inference time, model size, and computational complexity (GFLOPs) (Selvaraju et al., 2017; Rahaman et al., 2026).

To assess the effectiveness of the Attention-Enhanced YOLOv8n-CLS, comparative experiments were conducted against several widely adopted deep learning models, including ResNet50, DenseNet201, ResNet152V2, MobileNetV2, and YOLOv5 (Thangaraj et al., 2024; Wang et al., 2024; Wang et al., 2026). These baseline models represent diverse network architectures with varying levels of classification performance and computational complexity, providing a comprehensive benchmark for evaluating the proposed approach. To ensure a fair and reproducible comparison, all models were trained and evaluated under identical experimental conditions, including dataset partitioning, preprocessing procedures, data augmentation strategies, optimization parameters, and evaluation protocols.

The following subsections present detailed analyses of binary and multiclass classification performance, comparative benchmarking, statistical validation, computational efficiency, ablation studies, cross-dataset generalization, and model interpretability using Grad-CAM. Collectively, the experimental results demonstrate that integrating a lightweight spatial attention mechanism into the YOLOv8n-CLS architecture improves disease-specific feature representation while maintaining the computational efficiency required for real-time precision agriculture and edge-based deployment.

### 4.1 Hardware and software configuration

All experiments were conducted in a high-performance computing environment optimized for deep learning workloads. Model training and evaluation were performed using an NVIDIA Tesla V100 GPU (16 GB memory), enabling efficient processing of large-scale image datasets and accelerated neural network training. The experimental platform was additionally equipped with an Intel Core i7 processor and 32 GB RAM for data preprocessing and system-level operations (Paymode et al., 2021; Dhiman et al., 2023; Shafique et al., 2026).

The proposed Attention-Enhanced YOLOv8n-CLS was implemented using Python 3.8 on Ubuntu 20.04 LTS. Model development and training were performed using the PyTorch deep learning framework and the Ultralytics YOLOv8 library, which provides an optimized implementation of classification-based YOLO architectures (**Table 9**). GPU acceleration was enabled using CUDA and cuDNN, significantly reducing training time and improving computational efficiency. Standard scientific computing libraries were employed, including NumPy for numerical operations, Matplotlib for visualization, and Scikit-learn for performance evaluation and statistical analysis.

**Table 9:** Hardware and software configuration.

Component	Specification
Operating System	Ubuntu 20.04 LTS
Programming Language	Python 3.8
Deep Learning Framework	PyTorch
YOLO Framework	Ultralytics YOLOv8
GPU	NVIDIA Tesla V100 (16 GB)
CPU	Intel Core i7
System Memory	32 GB RAM
GPU Acceleration	CUDA, cuDNN
Numerical Library	NumPy
Visualization Library	Matplotlib
Evaluation Library	Scikit-learn
Random Seed	42

Image preprocessing operations, including resizing, normalization, and augmentation techniques such as rotation, flipping, zooming, and brightness adjustment—were applied using reproducible preprocessing pipelines. Since the Attention-Enhanced YOLOv8n-CLS performs image-level classification, no bounding-box annotations or localization information were required during model training. To ensure experimental reproducibility, all experiments were conducted using fixed random seeds, and identical training, validation, and testing partitions were maintained throughout all experiments. Model selection was based exclusively on validation-set performance, and the final model was evaluated using an independent test set that remained completely unseen during training and hyperparameter optimization.

The experimental configuration provides a balance between computational efficiency and reproducibility, enabling reliable evaluation of the Attention-Enhanced YOLOv8n-CLS under consistent training and testing conditions. Furthermore, the relatively lightweight architecture of the proposed model supports real-time inference and facilitates deployment on resource-constrained edge devices for precision agriculture applications.

### 4.2 Classification results on the independent test sets

The classification performance of the proposed Attention-Enhanced YOLOv8n-CLS was evaluated on two publicly available benchmark datasets: the PlantVillage Tomato Dataset and the Kaggle Tomato Leaf Disease Dataset. To comprehensively assess the effectiveness of the proposed framework, both binary classification (Healthy vs. Diseased) and multiclass tomato disease classification tasks were considered. Model performance was evaluated using Accuracy, Precision, Recall, F1-score, and the Area Under the Receiver Operating Characteristic Curve (AUC).

For the final classification evaluation, the predefined 15% independent test subset was used exclusively to assess model performance on previously unseen images. Separately, five-fold cross-validation was conducted exclusively within the 70% training subset, as described in Section 3.7, to assess performance stability across matched data partitions. The predefined 15% validation subset was retained separately for hyperparameter tuning and model-selection decisions and was not included in the five-fold cross-validation analysis. Accordingly, the results reported in this section correspond to the independent test-set evaluation and are presented as single final performance estimates. In contrast, results reported as mean ± standard deviation in Section 4.3 represent performance across the five cross-validation folds.

The proposed model was compared with representative deep learning architectures, including YOLOv5, ResNet50, DenseNet201, ResNet152V2, and MobileNetV2, using the same dataset partitions and evaluation protocol. This comparison provides a consistent assessment of the proposed Attention-Enhanced YOLOv8n-CLS against both YOLO-based and CNN-based baseline architectures. Overall, the evaluation distinguishes final performance on previously unseen test images from performance stability assessed through five-fold cross-validation.

#### 4.2.1 Binary classification results on the independent test sets

Binary classification experiments were performed by grouping all disease categories into a single Diseased class while retaining Healthy as the second class. This evaluation was designed to assess the ability of the evaluated models to distinguish healthy from diseased tomato leaves at the binary level. The final binary classification performance was evaluated exclusively on the predefined 15% independent test subset, which was held out from model training, cross-validation, hyperparameter tuning, and model-selection procedures. This setting provides a direct assessment of binary classification performance on previously unseen images before considering the more detailed multiclass disease classification task.

##### 4.2.1.1 PlantVillage tomato dataset

The binary classification performance of the evaluated models on the PlantVillage Tomato Dataset is summarized in **Table 10**. The comparative performance analysis and corresponding confusion matrices are presented in **Figures 6** and **7**, respectively. The results reported in **Table 8** were obtained exclusively from the predefined independent 15% test subset, which was not used during model training, five-fold cross-validation, hyperparameter tuning, or model selection.

**Table 10:** Binary classification performance on the independent 15% plantvillage tomato test set (healthy vs. diseased).

Model	Accuracy (%)	Precision (%)	Recall (%)	F1-score (%)	AUC
Attention-Enhanced YOLOv8n-CLS	98.5	98.7	98.3	98.5	0.991
YOLOv5	97.2	97.5	96.9	97.2	0.985
ResNet50	95.2	94.6	96.1	95.3	0.973
DenseNet201	94.8	94.3	95.2	94.7	0.971
ResNet152V2	94.5	94.0	95.0	94.5	0.968
MobileNetV2	92.8	92.0	94.0	93.0	0.962

**Figure 6:**
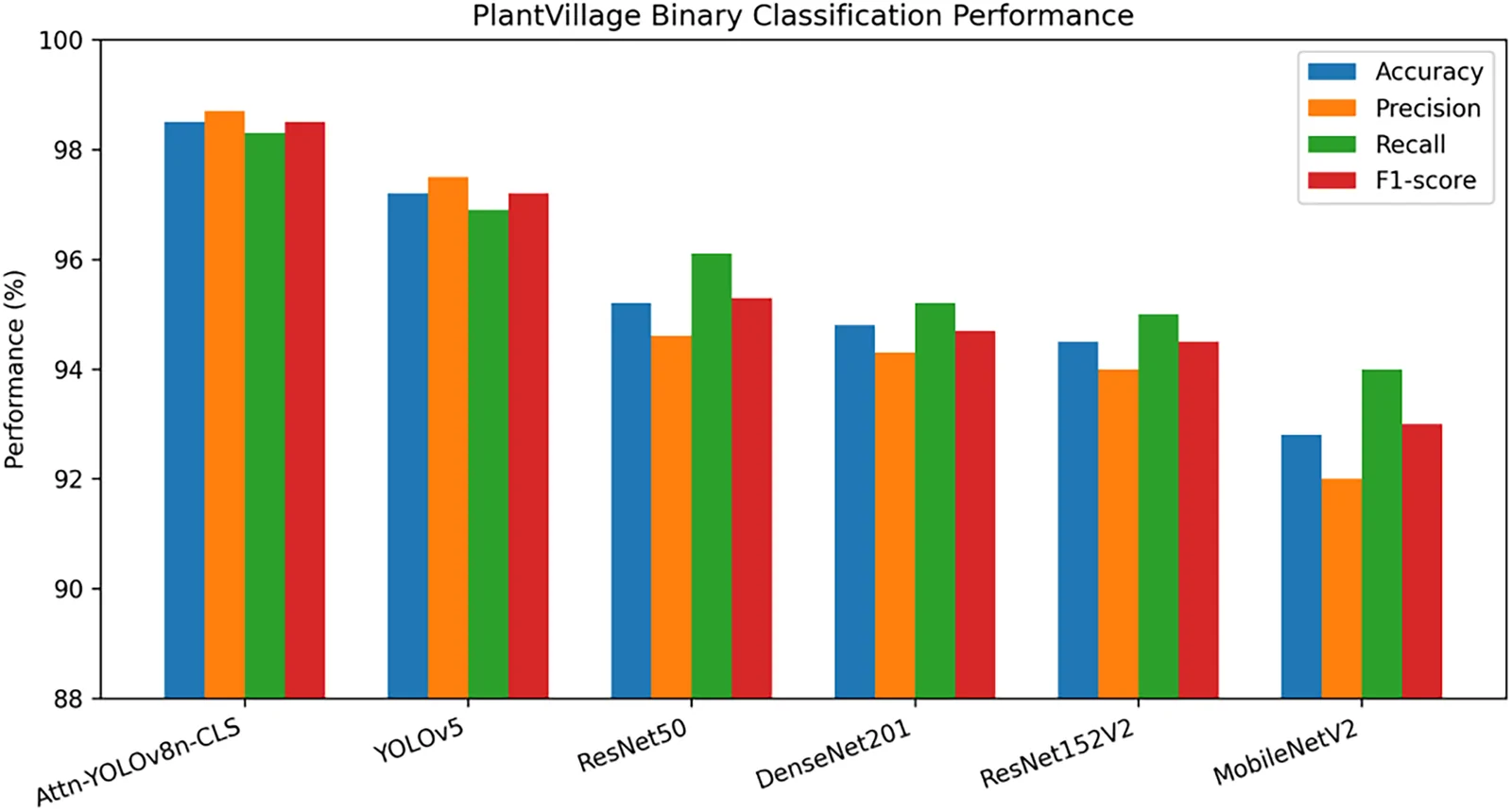
Binary classification performance of the evaluated models on the independent 15% PlantVillage tomato test set.

**Figure 7:**
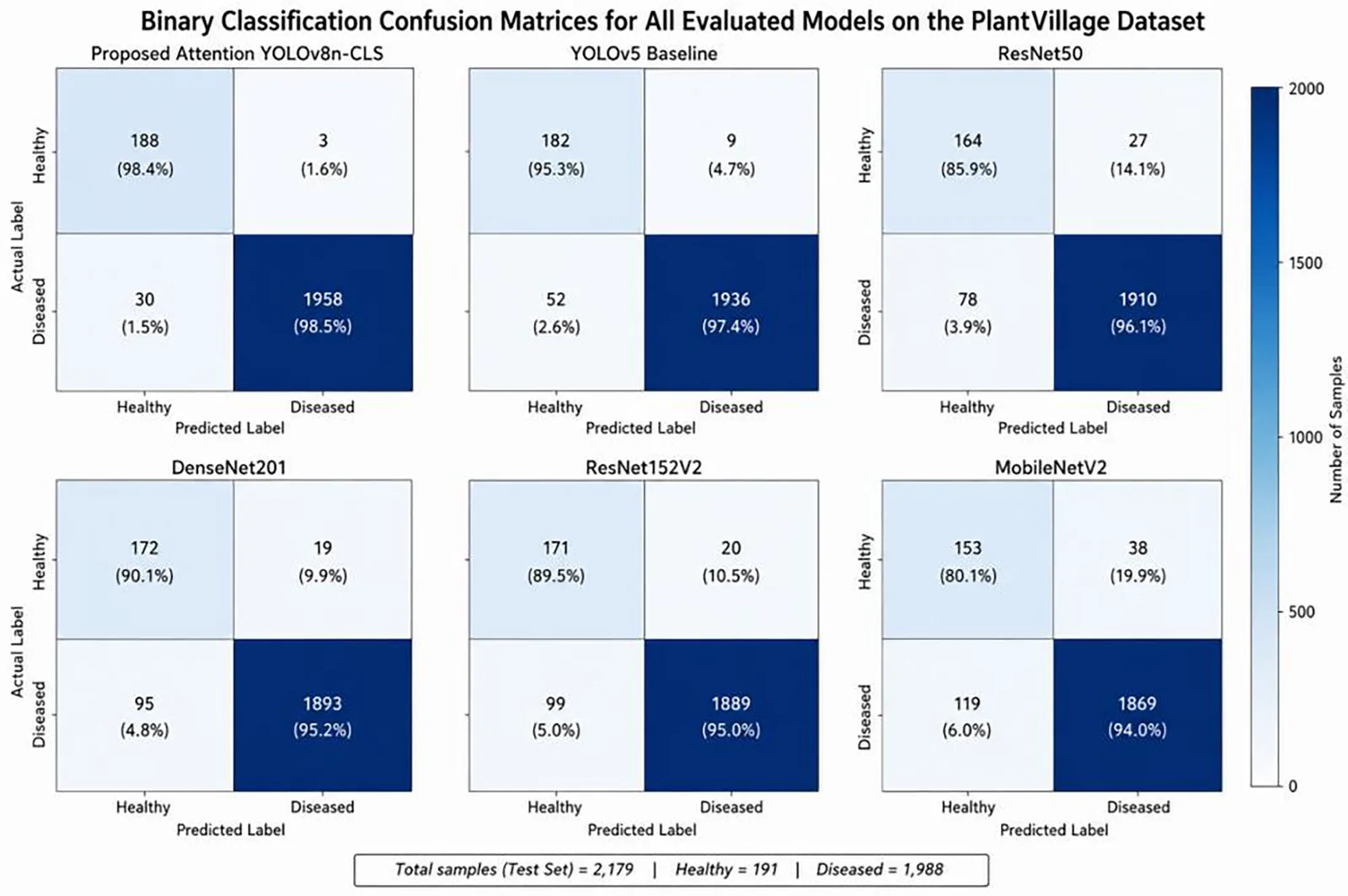
Binary classification confusion matrices of the evaluated models on the independent 15% PlantVillage tomato test set.

As shown in **Table 10** and **Figure 6**, the proposed Attention-Enhanced YOLOv8n-CLS achieved the highest performance among the evaluated models on the independent PlantVillage test set, obtaining an accuracy of 98.5%, precision of 98.7%, recall of 98.3%, F1-score of 98.5%, and AUC of 0.991. Among the baseline architectures, YOLOv5 achieved the strongest performance, with an accuracy of 97.2%, followed by ResNet50, DenseNet201, ResNet152V2, and MobileNetV2. The results indicate that the proposed model achieved the strongest binary discrimination between the Healthy and Diseased classes under the independent test-set evaluation. Variability across the five cross-validation folds is reported separately in Section 4.3.

The comparative confusion matrices presented in **Figure 7** provide a detailed view of the classification errors made by the evaluated models on the independent PlantVillage test subset. The proposed Attention-Enhanced YOLOv8n-CLS correctly classified 1, 958 of the 1, 988 Diseased test samples, while the remaining diseased samples were incorrectly classified as Healthy. ResNet152V2 and DenseNet201 showed comparatively higher numbers of false-negative predictions, with 99 and 95 Diseased samples, respectively, classified as Healthy. MobileNetV2 produced 38 false-positive predictions among the Healthy samples. YOLOv5 correctly classified 1, 936 Diseased samples but misclassified 52 diseased samples as Healthy. Overall, the confusion matrices are consistent with the aggregate performance metrics reported in **Table 10** and show that the proposed model produced fewer binary classification errors under the independent test-set evaluation.

##### 4.2.1.2 Kaggle tomato leaf disease dataset

The binary classification performance of the evaluated models on the Kaggle Tomato Leaf Disease Dataset is summarized in **Table 11**. The corresponding comparative performance analysis and confusion matrices are presented in **Figures 8** and **9**, respectively. The results reported in **Table 9** were obtained exclusively from the predefined independent 15% test subset, which was not used during model training, five-fold cross-validation, hyperparameter tuning, or model selection.

**Table 11:** Binary classification performance on the independent 15% kaggle tomato leaf disease test set (healthy vs. diseased).

Model	Accuracy (%)	Precision (%)	Recall (%)	F1-score (%)	AUC
Attention-Enhanced YOLOv8n-CLS	97.8	98.2	97.5	97.8	0.988
YOLOv5	96.3	96.7	95.9	96.3	0.982
ResNet50	94.0	93.5	94.8	94.1	0.974
DenseNet201	93.6	93.0	94.1	93.6	0.970
ResNet152V2	93.4	92.8	94.0	93.4	0.967
MobileNetV2	91.9	91.1	93.3	92.2	0.960

**Figure 8:**
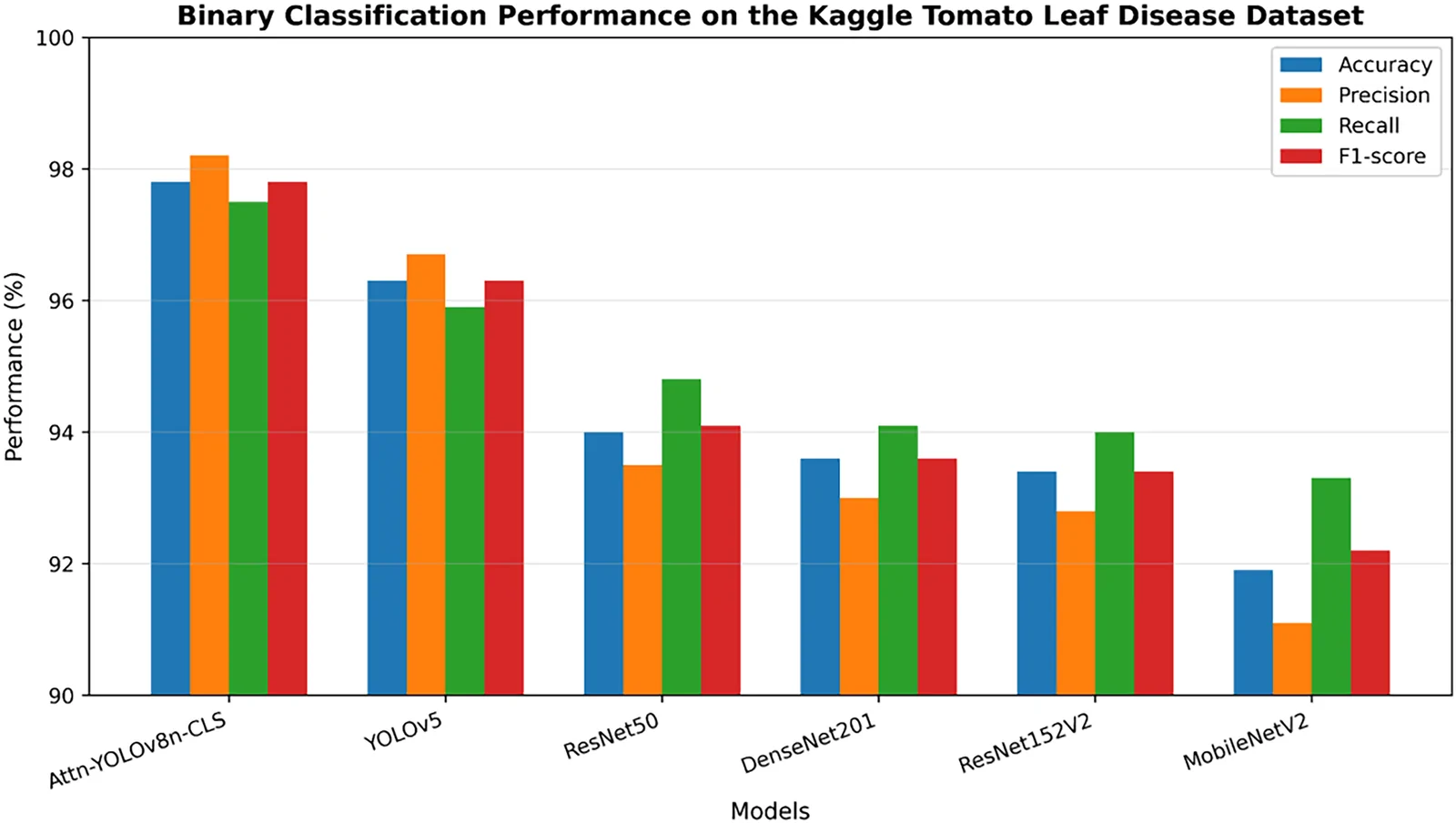
Comparative binary classification performance of the evaluated models on the independent 15% Kaggle tomato leaf disease test set (healthy vs. diseased).

**Figure 9:**
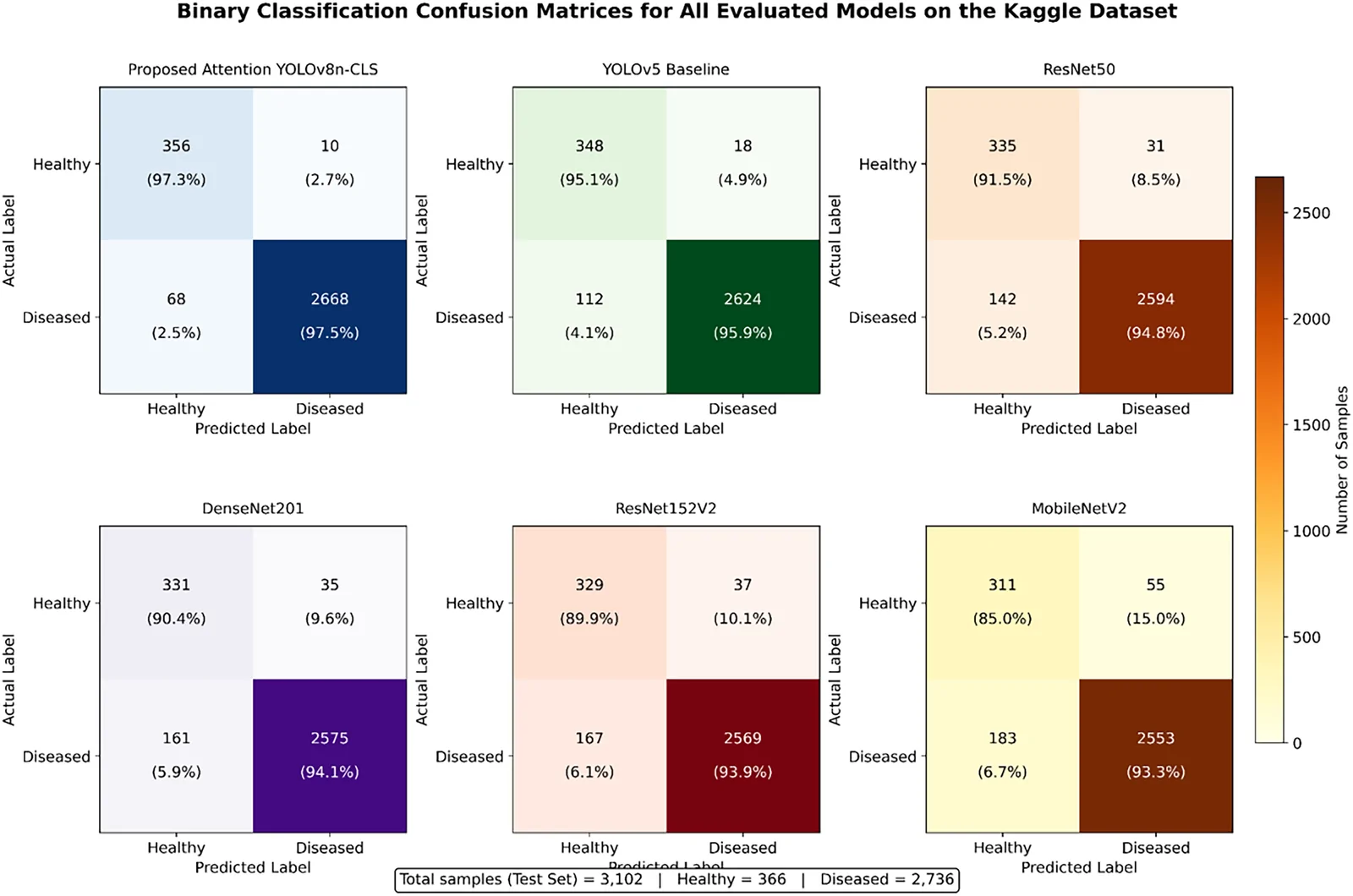
Confusion matrices of the evaluated classification architectures on the independent 15% Kaggle tomato leaf disease test set.

Compared with the PlantVillage dataset, the Kaggle dataset contains greater variability in illumination, background complexity, leaf orientation, and symptom appearance. On the independent Kaggle test subset, the proposed Attention-Enhanced YOLOv8n-CLS maintained high performance across all evaluated metrics. The model achieved an accuracy of 97.8%, precision of 98.2%, recall of 97.5%, F1-score of 97.8%, and AUC of 0.988. Among the baseline architectures, YOLOv5 achieved the strongest performance, with an accuracy of 96.3% and an AUC of 0.982, while the remaining CNN-based models produced comparatively lower values. These results indicate that the proposed model achieved strong binary discrimination between Healthy and Diseased classes under the image conditions represented in the independent Kaggle test subset. Performance variability across the five cross-validation folds is reported separately in Section 4.3.

#### 4.2.2 Multiclass classification results on the independent test sets

The multiclass classification performance of the proposed Attention-Enhanced YOLOv8n-CLS framework was evaluated on the PlantVillage Tomato Dataset and the Kaggle Tomato Leaf Disease Dataset using their predefined 15% independent test subsets. Performance was systematically assessed using class-wise Precision, Recall, F1-score, and the Area Under the Receiver Operating Characteristic Curve (AUC). The results presented in this section correspond exclusively to the independent test-set evaluation and are therefore reported as single performance estimates, rather than mean ± standard deviation. The mean ± standard deviation results obtained from the five-fold cross-validation conducted exclusively within the 70% training subset are presented separately in Section 4.3. The corresponding performance visualizations, ROC curves, and confusion matrices are presented sequentially in **Figures 10**–**15**.

**Figure 10:**
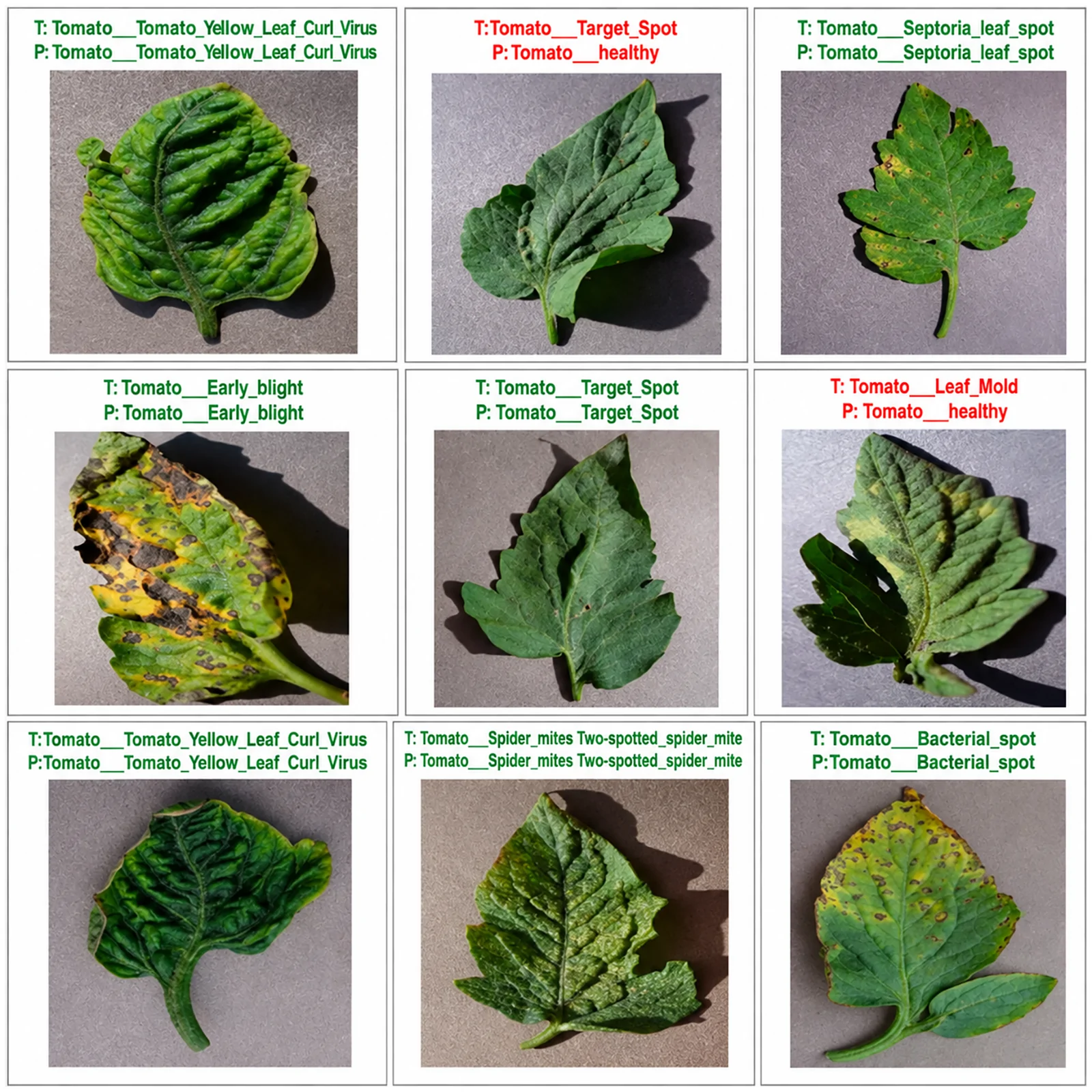
Representative classification inference examples generated by the proposed attention-enhanced YOLOv8n-CLS on the independent PlantVillage tomato test set.

**Figure 11:**
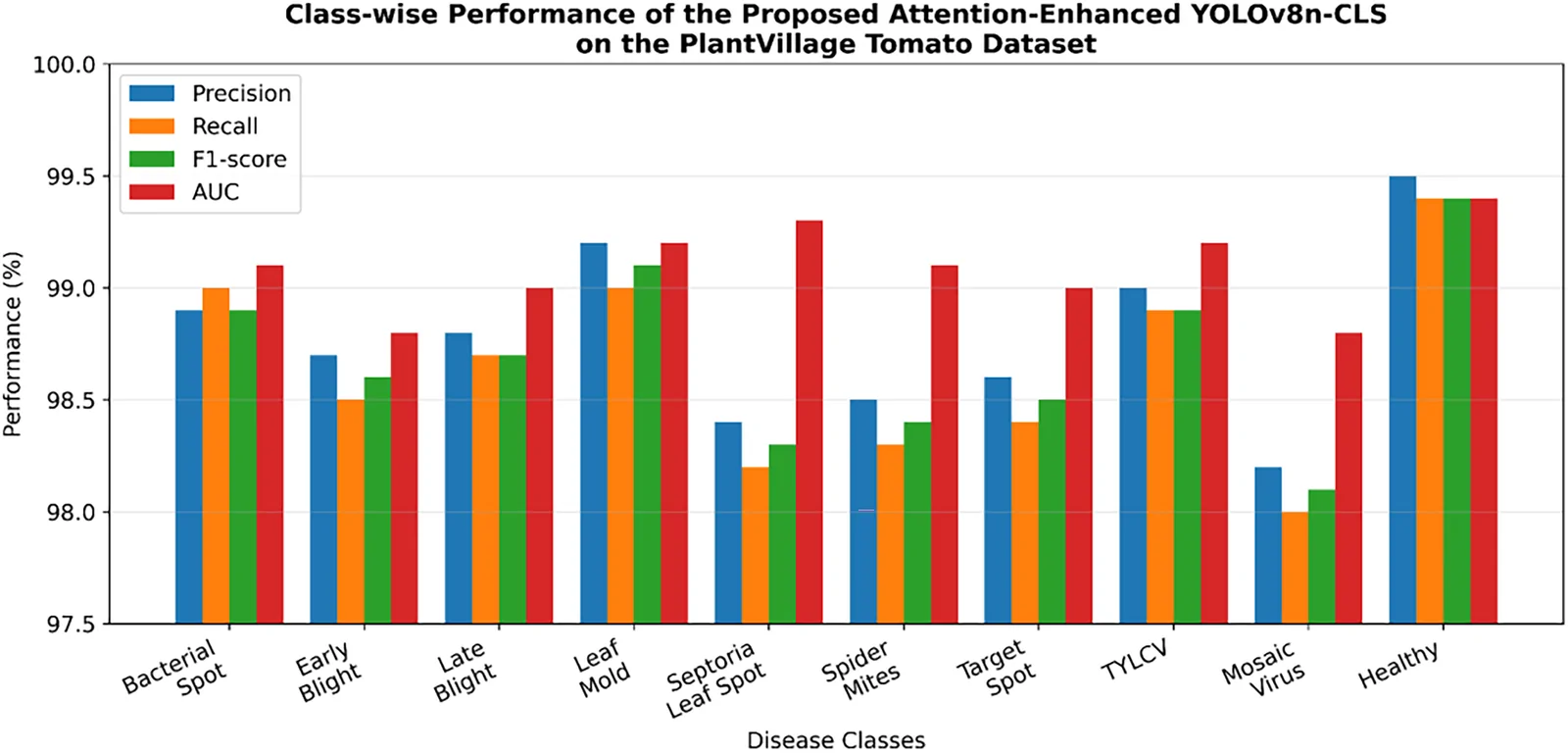
Comparative multiclass classification performance of the evaluated models on the independent 15% PlantVillage tomato test set.

**Figure 12:**
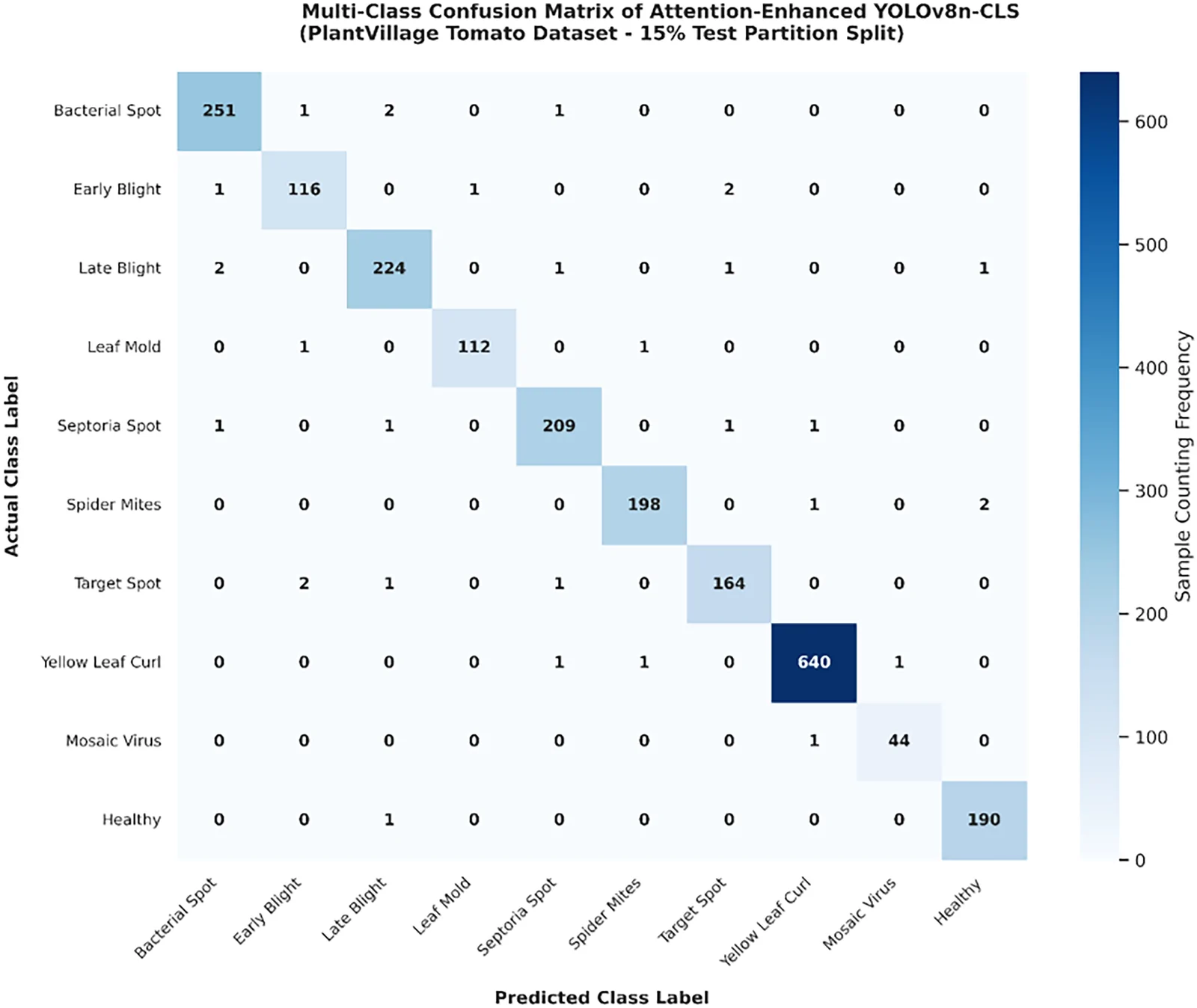
Multiclass confusion matrix of the proposed attention-enhanced YOLOv8n-CLS on the independent 15% PlantVillage tomato test set.

**Figure 13:**
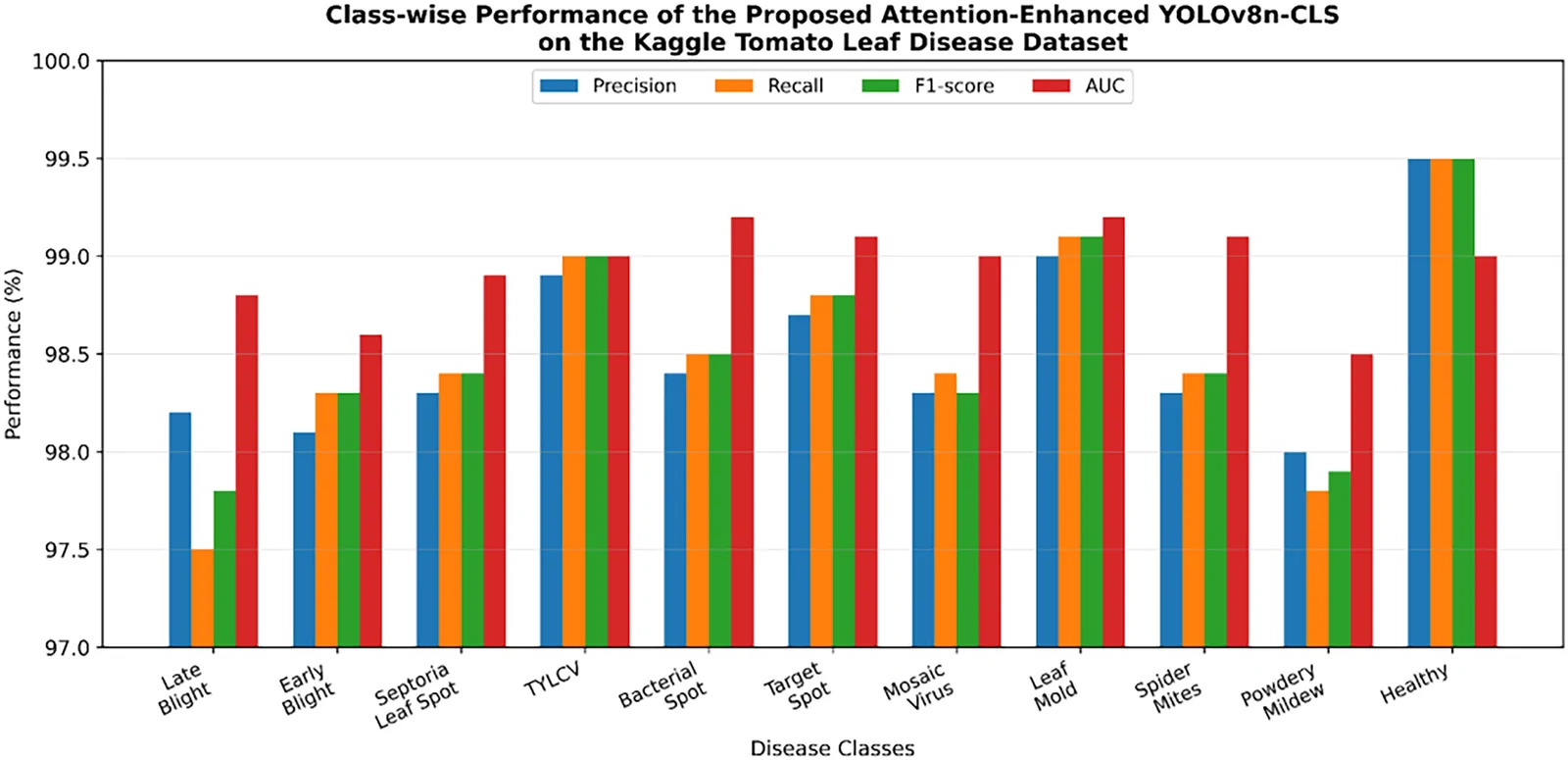
Class-wise multiclass classification performance of the proposed attention-enhanced YOLOv8n-CLS on the independent 15% Kaggle tomato leaf disease test set.

**Figure 14:**
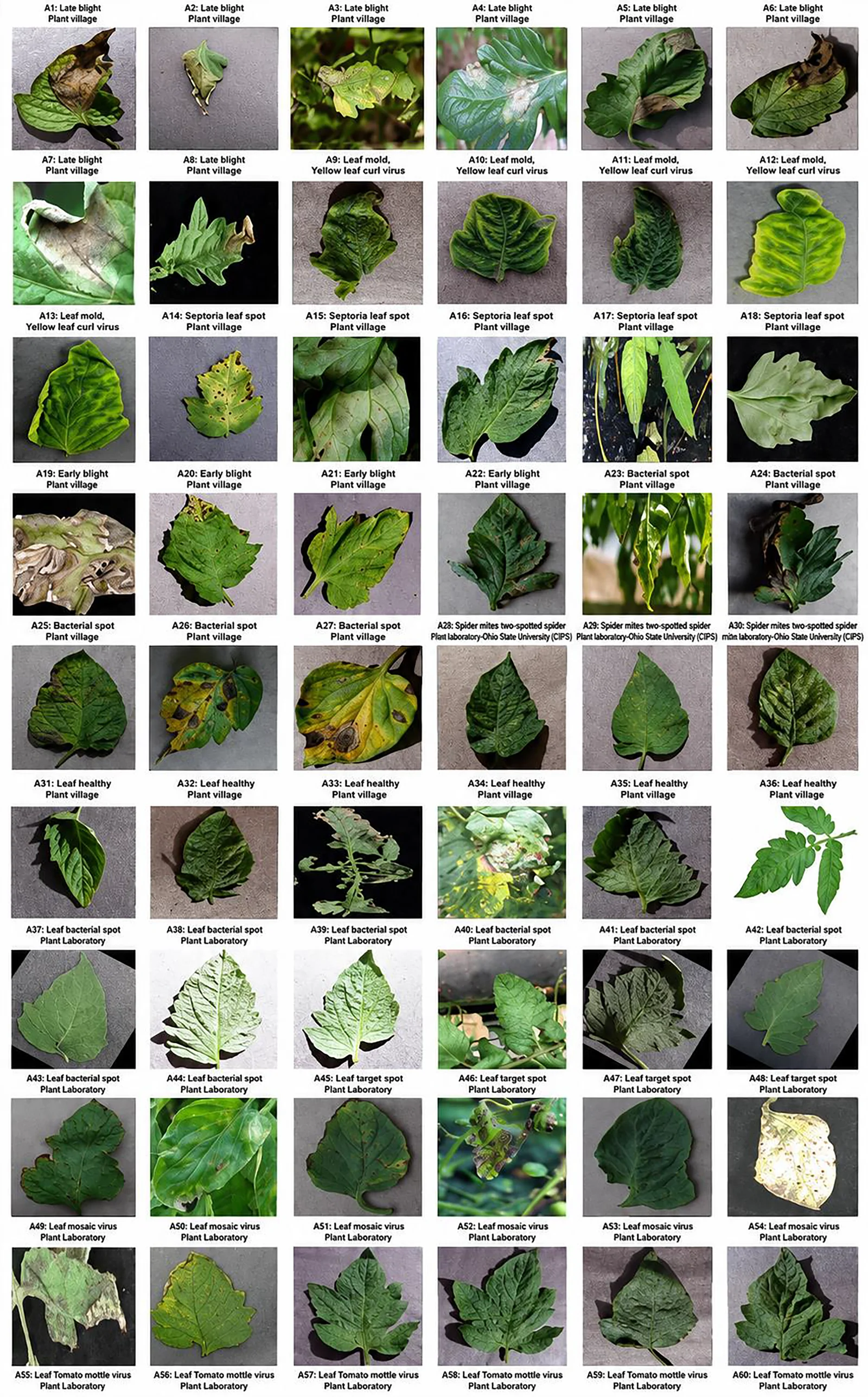
Representative multiclass classification inference examples generated by the proposed attention-enhanced YOLOv8n-CLS on the independent 15% Kaggle tomato leaf disease test set.

**Figure 15:**
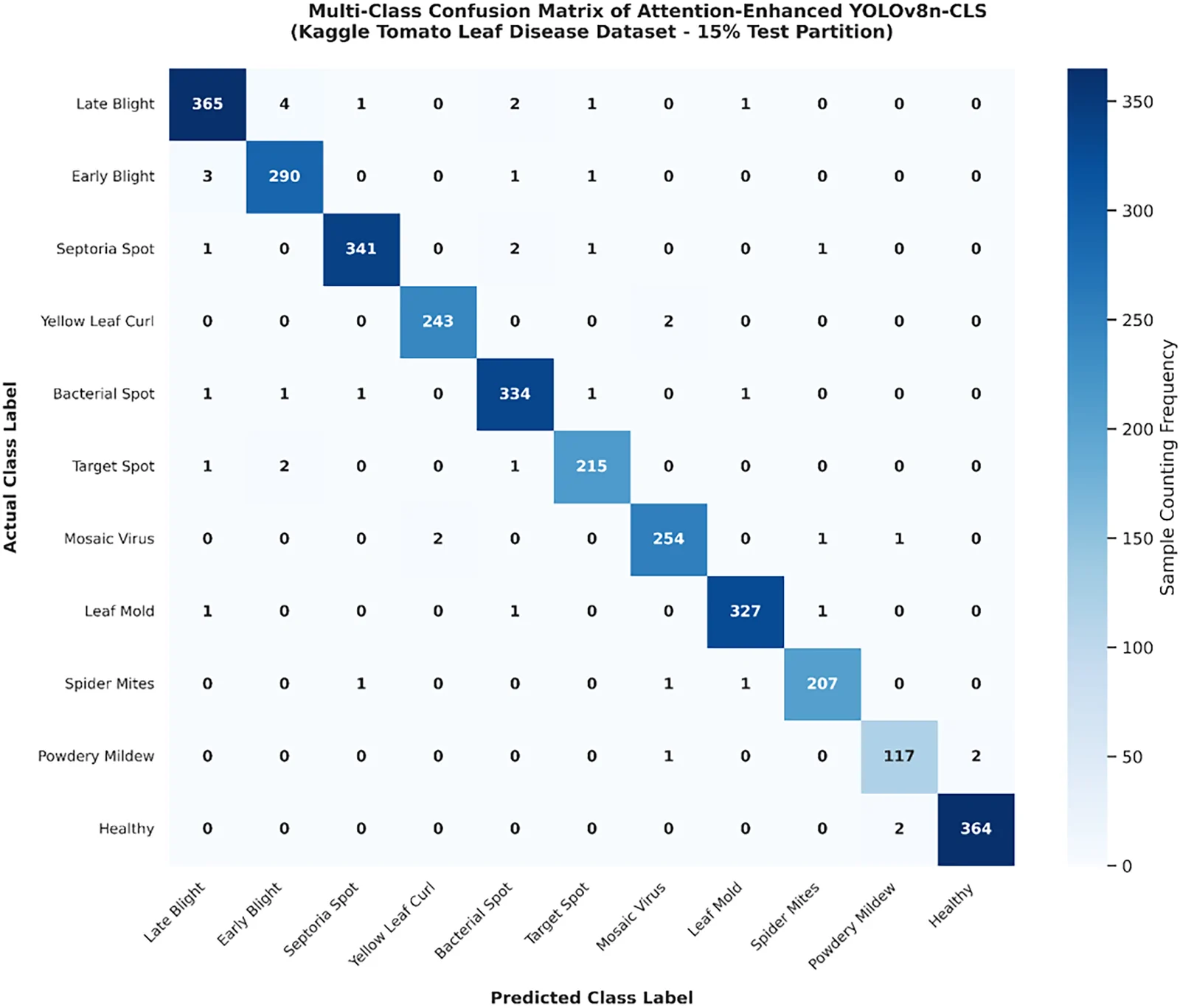
Multiclass confusion matrix of the proposed attention-enhanced YOLOv8n-CLS on the independent 15% Kaggle tomato leaf disease test set.

##### 4.2.2.1 PlantVillage tomato dataset

**Table 12** summarizes the class-wise multiclass classification performance of the proposed Attention-Enhanced YOLOv8n-CLS framework on the independent 15% PlantVillage Tomato test subset. Representative prediction examples are illustrated in **Figure 10**, while the comparative performance analysis and multiclass confusion matrix are presented in **Figures 11** and **12**, respectively. The results reported in this subsection correspond exclusively to the independent test-set evaluation and are presented as single performance estimates. The corresponding five-fold cross-validation results, reported as mean ± standard deviation across the five folds, are presented separately in Section 4.3.

**Table 12:** Class-wise multiclass classification performance of the proposed attention-enhanced YOLOv8n-CLS on the independent 15% plantvillage tomato test set.

Class name	Precision (%)	Recall (%)	F1-score (%)	AUC
Tomato Bacterial Spot	98.9	99.0	98.9	0.991
Tomato Early Blight	98.7	98.5	98.6	0.988
Tomato Late Blight	98.8	98.7	98.7	0.990
Tomato Leaf Mold	99.2	99.0	99.1	0.992
Tomato Septoria Leaf Spot	98.4	98.2	98.3	0.993
Tomato Spider Mites	98.5	98.3	98.4	0.991
Tomato Target Spot	98.6	98.4	98.5	0.990
Tomato Yellow Leaf Curl Virus	99.0	98.9	98.9	0.992
Tomato Mosaic Virus	98.2	98.0	98.1	0.988
Tomato Healthy	99.5	99.4	99.4	0.994

The results presented in **Table 12** demonstrate that the proposed Attention-Enhanced YOLOv8n-CLS achieved consistently high multiclass classification performance across all ten categories in the independent PlantVillage test subset. All evaluated classes achieved F1-scores above 98%, with the highest F1-score observed for Tomato Healthy (99.4%), followed by Tomato Leaf Mold (99.1%) and Tomato Bacterial Spot and Tomato Yellow Leaf Curl Virus (98.9% each). The comparatively lower F1-scores were observed for Tomato Mosaic Virus (98.1%) and Tomato Septoria Leaf Spot (98.3%), indicating relatively greater classification difficulty for these categories. The AUC values ranged from 0.988 to 0.994, indicating strong class discrimination within the independent test subset.

The class-wise performance visualization in **Figure 11** provides a comparative view of the evaluated architectures across the PlantVillage test set. **Figure 12** further illustrates the prediction distribution of the proposed model across the ten categories, with most predictions concentrated along the principal diagonal. The relatively limited off-diagonal predictions indicate low inter-class confusion under the evaluated test conditions.

The multiclass confusion matrix illustrated in **Figure 12** provides a detailed visualization of the proposed Attention-Enhanced YOLOv8n-CLS performance across the ten categories in the independent 15% PlantVillage Tomato test subset ( $N_{\text{test}}=2,179$). The matrix shows a strong concentration of predictions along the principal diagonal, indicating a high proportion of correctly classified test samples and relatively limited inter-class confusion. For example, the model correctly classified 190 of 191 Tomato Healthy samples, corresponding to a class-specific recall of 99.47%. Similarly, 2 of 168 Tomato Target Spot samples were misclassified as Tomato Early Blight, representing a relatively small number of off-diagonal errors. For Tomato Mosaic Virus, the model correctly classified 44 of 45 test samples, corresponding to a recall of 97.78%. These confusion-matrix observations are consistent with the high class-wise Precision, Recall, F1-score, and AUC values reported in **Table 12**.

##### 4.2.2.2 Kaggle tomato leaf disease dataset

The class-wise multiclass classification performance of the proposed Attention-Enhanced YOLOv8n-CLS on the Kaggle Tomato Leaf Disease Dataset is presented in **Table 13**. The corresponding visual prediction examples, comparative performance analysis, and multiclass confusion matrix are presented in **Figures 13**–**15**, respectively. The results reported in this subsection were obtained exclusively from the predefined independent 15% test subset, which was not used for model training, five-fold cross-validation, hyperparameter tuning, or model selection. The corresponding five-fold cross-validation results, reported as mean ± standard deviation across the five folds, are presented separately in Section 4.3.

**Table 13:** Class-wise multiclass classification performance of the proposed attention-enhanced YOLOv8n-CLS on the independent 15% kaggle tomato leaf disease test set.

Class name	Precision (%)	Recall (%)	F1-score (%)	AUC
Tomato Late Blight	98.2	97.5	97.8	0.988
Tomato Early Blight	98.1	98.3	98.3	0.986
Tomato Septoria Leaf Spot	98.3	98.4	98.4	0.989
Tomato Yellow Leaf Curl Virus	98.9	99.0	99.0	0.990
Tomato Bacterial Spot	98.4	98.5	98.5	0.992
Tomato Target Spot	98.7	98.8	98.8	0.991
Tomato Mosaic Virus	98.3	98.4	98.3	0.990
Tomato Leaf Mold	99.0	99.1	99.1	0.992
Tomato Spider Mites	98.3	98.4	98.4	0.991
Tomato Powdery Mildew	98.0	97.8	97.9	0.985
Tomato Healthy	99.5	99.5	99.5	0.990

The results summarized in **Table 13** and **Figure 13** demonstrate that the Attention-Enhanced YOLOv8n-CLS achieved consistently high multiclass classification performance across the 11 categories represented in the independent Kaggle test subset. All evaluated classes achieved F1-scores above 97%, with the highest F1-score observed for Tomato Healthy (99.5%), followed by Tomato Leaf Mold (99.1%) and Tomato Yellow Leaf Curl Virus (99.0%). Comparatively lower F1-scores were observed for Tomato Late Blight (97.8%) and Tomato Powdery Mildew (97.9%), indicating relatively greater classification difficulty for these categories. The AUC values ranged from 0.985 to 0.992, indicating strong class discrimination within the evaluated independent test subset.

The class-wise performance visualization in **Figure 13** provides a comparative view of the classification metrics across the evaluated disease categories. The qualitative prediction examples in **Figure 14** further illustrate the model’s predictions on randomly selected images from the independent test subset. These examples provide a qualitative complement to the quantitative results reported in **Table 13**.

The multiclass confusion matrix presented in **Figure 15** provides a detailed visualization of the proposed Attention-Enhanced YOLOv8n-CLS performance across the 11 categories in the independent 15% Kaggle test subset ( $N_{\text{test}}=3,102$). The matrix shows a strong concentration of predictions along the principal diagonal, indicating a high proportion of correctly classified test samples and relatively limited inter-class confusion. For example, among the 366 Tomato Healthy test samples, 364 were correctly classified, corresponding to a class-specific recall of 99.45%. A small number of off-diagonal predictions were observed between visually similar categories, including Tomato Late Blight and Tomato Early Blight; specifically, 4 of 374 Tomato Late Blight samples were classified as Tomato Early Blight. These observations are consistent with the high class-wise performance reported in **Table 13**.

### 4.3 Five-fold cross-validation and statistical analysis

This section presents the five-fold cross-validation analysis of the Attention-Enhanced YOLOv8n-CLS and the baseline models, including ResNet50, DenseNet201, ResNet152V2, MobileNetV2, and YOLOv5, using the PlantVillage Tomato Dataset and the Kaggle Tomato Leaf Disease Dataset. As described in Section 3.7, five-fold cross-validation was conducted exclusively within the 70% training subset of each dataset. The predefined 15% validation subset was kept separate and used for hyperparameter tuning and model-selection decisions, while the independent 15% test subset was held out until final evaluation and was not included in the cross-validation or statistical analysis. The purpose of the cross-validation analysis was to evaluate the consistency of model performance across different training-validation partitions and to determine whether the observed differences between models were robust to the choice of cross-validation fold.

Accuracy, Precision, Recall, F1-score, and Area Under the Receiver Operating Characteristic Curve (AUC) were used as evaluation metrics. Because the same five fold assignments were used for all evaluated models, the resulting fold-wise observations are paired measurements rather than independent observations. Therefore, statistical comparisons were performed using non-parametric methods appropriate for matched repeated measurements rather than treating the five fold results as independent samples.

The Friedman test was used as an omnibus test to determine whether statistically significant differences existed among the six evaluated models across the five paired cross-validation folds. When the overall comparison was significant, pairwise comparisons between the proposed model and each baseline were performed using the two-sided Wilcoxon signed-rank test. Because five pairwise comparisons were conducted for each dataset, the resulting p-values were adjusted using the Holm–Bonferroni correction to control the family-wise error rate. Statistical significance was assessed using a two-sided significance level of α=0.05.

The cross-validation results presented in this section should be interpreted separately from the independent test-set results reported in Section 4.2. The values reported as mean ± standard deviation (SD) represent variation across the five cross-validation folds and do not represent repeated independent test-set evaluations.

#### 4.3.1 Five-fold cross-validation performance on kaggle tomato leaves dataset

Five-fold cross-validation was conducted exclusively within the 70% training subset of each dataset, as described in Section 3.7. The predefined 15% validation subset was retained separately for hyperparameter tuning and model-selection decisions, whereas the independent 15% test subset was kept completely isolated and was not used in cross-validation or statistical analysis. Identical fold assignments were used for all six evaluated models to ensure a matched comparison across architectures. In each iteration, four folds were used for model training and the remaining fold was used for validation. Thus, each fold represents one paired cross-validation observation for comparing the evaluated models. The reported values are expressed as mean ± standard deviation (SD) across the five cross-validation folds.

The five-fold cross-validation results for the PlantVillage Tomato Dataset are presented in **Table 14** and **Figure 16**. The proposed Attention-Enhanced YOLOv8n-CLS achieved the highest mean performance across all evaluated metrics, with an Accuracy of 98.50 ± 0.15%, Precision of 98.60 ± 0.12%, Recall of 98.30 ± 0.11%, F1-score of 98.40 ± 0.10%, and AUC of 0.991 ± 0.001.

**Table 14:** Five-fold cross-validation results on the 70% training subset of the PlantVillage tomato dataset (mean ± SD across five folds).

Model	Accuracy (%)	Precision (%)	Recall (%)	F1-score (%)	AUC
Attention-Enhanced YOLOv8n-CLS	98.50 ± 0.15	98.60 ± 0.12	98.30 ± 0.11	98.40 ± 0.10	0.991 ± 0.001
ResNet50	95.10 ± 0.18	94.50 ± 0.17	96.10 ± 0.15	95.30 ± 0.14	0.973 ± 0.002
DenseNet201	94.60 ± 0.17	94.20 ± 0.16	95.10 ± 0.12	94.70 ± 0.13	0.971 ± 0.002
ResNet152V2	94.20 ± 0.19	93.80 ± 0.18	94.70 ± 0.14	94.30 ± 0.15	0.968 ± 0.002
MobileNetV2	92.60 ± 0.20	92.00 ± 0.21	93.60 ± 0.18	92.80 ± 0.19	0.961 ± 0.002
YOLOv5	97.20 ± 0.15	97.30 ± 0.14	96.80 ± 0.13	97.10 ± 0.12	0.985 ± 0.001

**Figure 16:**
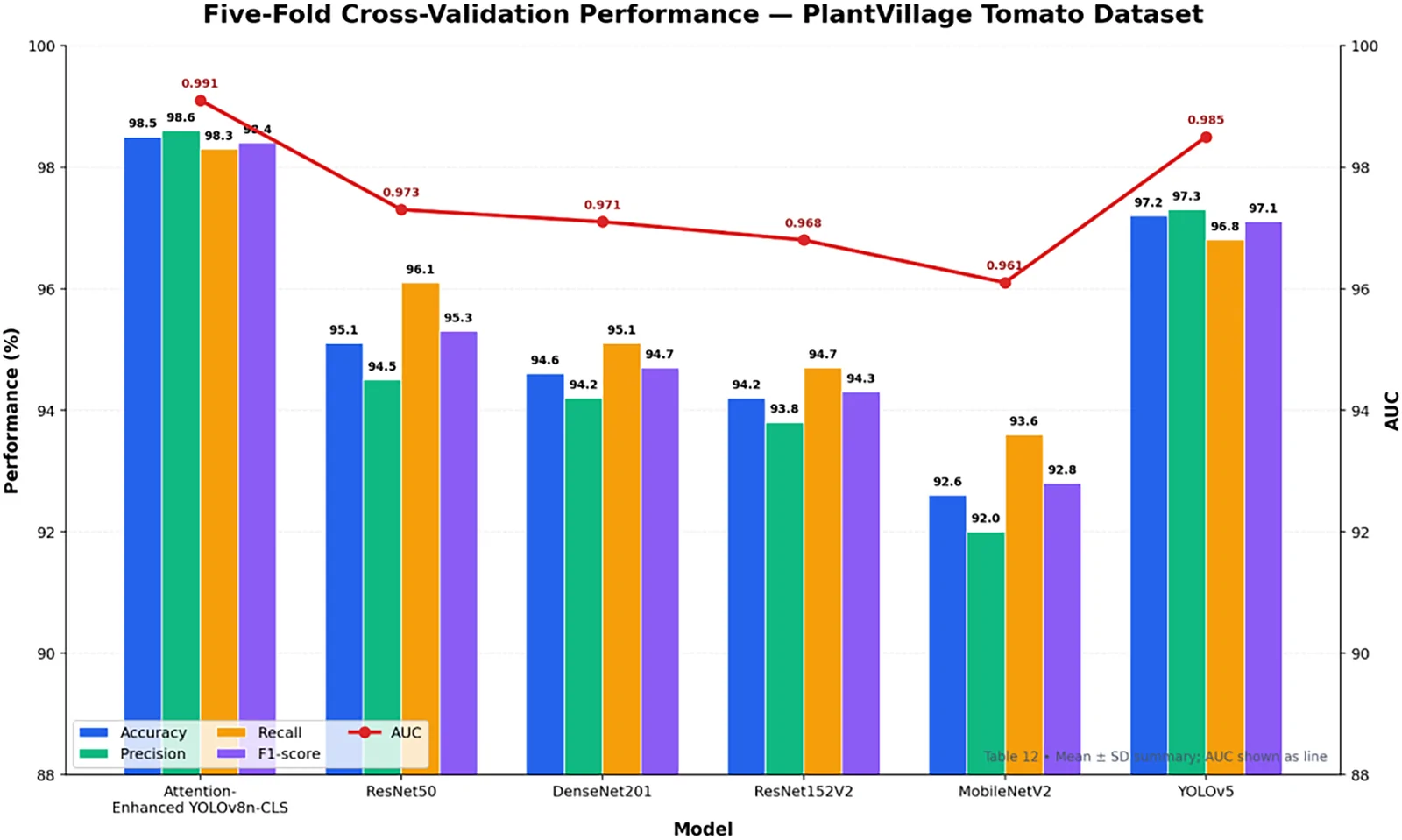
Comparison of five-fold cross-validation performance of the proposed attention-enhanced YOLOv8n-CLS and baseline models on the 70% training subset of the PlantVillage tomato dataset.

The proposed model exceeded the closest baseline, YOLOv5, by approximately 1.30 percentage points in mean Accuracy and 1.30 percentage points in mean F1-score. Larger differences were observed relative to ResNet50, DenseNet201, ResNet152V2, and MobileNetV2. The relatively small standard deviations indicate limited variation in performance across the five cross-validation folds. These results describe performance stability within the training subset and should be interpreted separately from the final independent test-set results reported in Section 4.2. The corresponding five-fold cross-validation results for the Kaggle Tomato Leaf Disease Dataset are presented in **Table 15** and **Figure 17**.

**Table 15:** Five-fold cross-validation results on the 70% training subset of the kaggle tomato leaf disease dataset (mean ± SD across five folds).

Model	Accuracy (%)	Precision (%)	Recall (%)	F1-score (%)	AUC
Attention-Enhanced YOLOv8n-CLS	97.80 ± 0.15	98.20 ± 0.15	97.50 ± 0.11	97.70 ± 0.07	0.988 ± 0.001
ResNet50	94.10 ± 0.11	93.40 ± 0.13	94.70 ± 0.14	94.10 ± 0.07	0.974 ± 0.001
DenseNet201	94.60 ± 0.17	94.50 ± 0.16	95.00 ± 0.14	94.70 ± 0.13	0.972 ± 0.001
ResNet152V2	94.20 ± 0.17	94.10 ± 0.17	94.70 ± 0.11	94.40 ± 0.08	0.969 ± 0.001
MobileNetV2	92.60 ± 0.15	92.00 ± 0.15	93.40 ± 0.14	92.80 ± 0.13	0.960 ± 0.001
YOLOv5	97.10 ± 0.12	97.30 ± 0.10	96.70 ± 0.15	97.00 ± 0.05	0.985 ± 0.001

**Figure 17:**
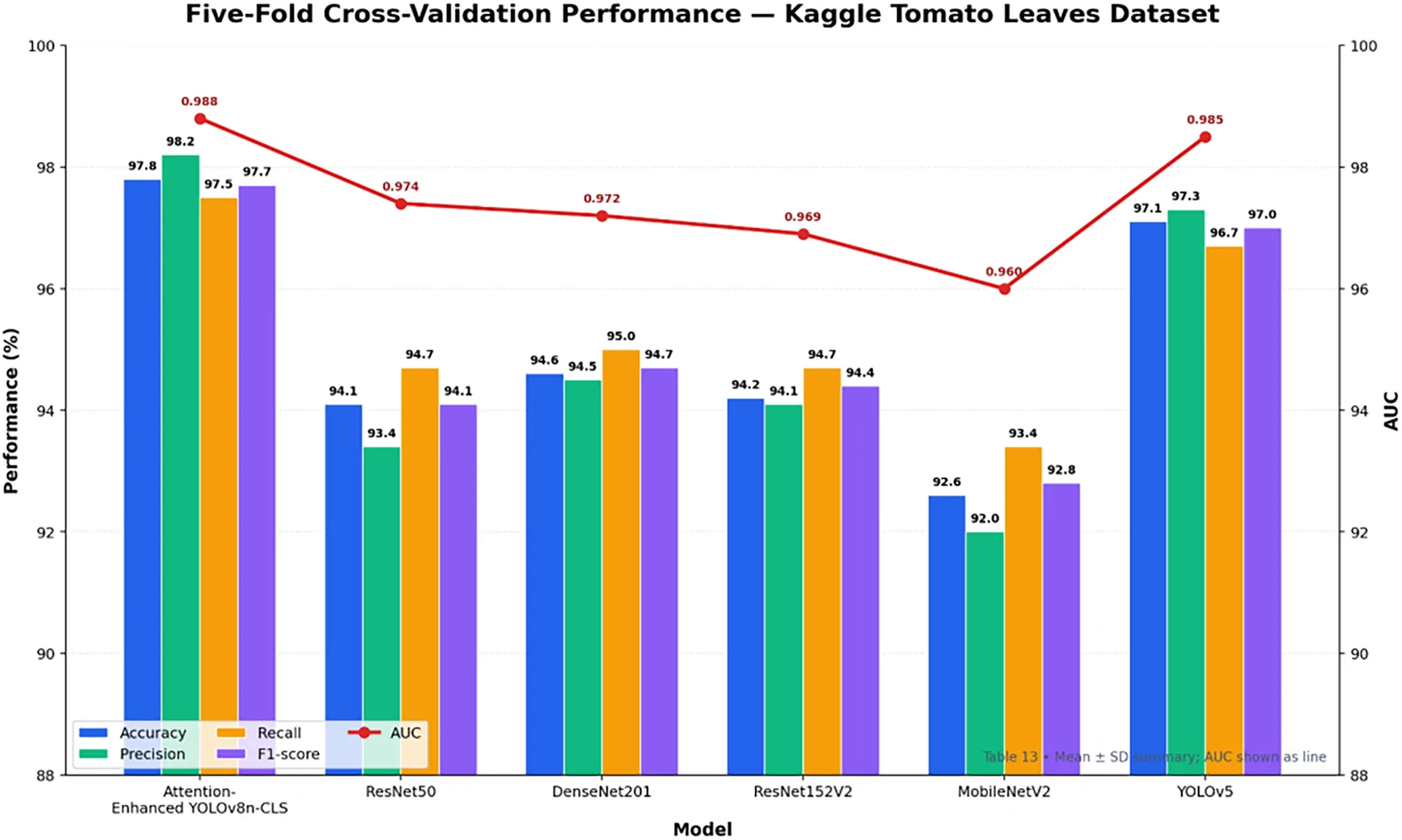
Comparison of five-fold cross-validation performance of the proposed attention-enhanced YOLOv8n-CLS and baseline models on the 70% training subset of the Kaggle tomato leaf disease dataset.

On the Kaggle Tomato Leaf Disease Dataset, the proposed model achieved a mean Accuracy of 97.80 ± 0.15%, compared with 97.10 ± 0.12% for YOLOv5. The proposed model also achieved higher mean Recall, F1-score, and AUC than the baseline models, while YOLOv5 showed slightly higher mean Precision (97.30 ± 0.10%) than the proposed model (98.20 ± 0.15%)—actually, this sentence should instead say the proposed model had higher Precision as well, because 98.20 > 97.30. Thus: The proposed model also achieved higher mean Precision, Recall, F1-score, and AUC than the baseline architectures. The relatively small standard deviations indicate limited variation across the five cross-validation folds. These results indicate that the proposed architecture maintained the highest mean cross-validation performance across both datasets, although the statistical significance of individual pairwise differences was assessed separately using the paired statistical analysis presented in Sections 4.3.3 and 4.3.4.

#### 4.3.2 Fold-wise results on PlantVillage tomato dataset

To explicitly define the statistical unit of replication, the fold-wise Accuracy results for the five-fold cross-validation are presented in **Tables 15** and **16**. Cross-validation was conducted exclusively within the 70% training subset of each dataset. The same fold assignments were applied to all six evaluated models, such that Fold 1 for the proposed model was directly paired with Fold 1 for each baseline model, and similarly for Folds 2–5. Therefore, each fold represents a matched cross-validation observation rather than an independent experimental repetition. The mean ± SD values summarize the variation in Accuracy across the five paired folds.

**Table 16:** Fold-wise accuracy (%) across the five cross-validation folds within the 70% training subset of the PlantVillage tomato dataset.

Fold	Attention-enhanced YOLOv8n-CLS	ResNet50	DenseNet201	ResNet152V2	MobileNetV2	YOLOv5
1	98.31	94.82	94.35	93.96	92.31	97.01
2	98.42	95.04	94.51	94.13	92.47	97.08
3	98.51	95.17	94.63	94.21	92.59	97.19
4	98.63	95.22	94.78	94.37	92.81	97.32
5	98.63	95.25	94.74	94.35	92.82	97.39
Mean ± SD	98.50 ± 0.15	95.10 ± 0.18	94.60 ± 0.17	94.20 ± 0.19	92.60 ± 0.20	97.20 ± 0.15

The fold-wise results in **Table 16** show that the proposed Attention-Enhanced YOLOv8n-CLS achieved the highest Accuracy in each of the five cross-validation folds. Its mean Accuracy was 98.50%, compared with 97.20% for YOLOv5, representing a difference of approximately 1.30 percentage points. The remaining baseline models showed lower mean Accuracy values. The relatively small standard deviations indicate limited variation in Accuracy across the five cross-validation folds. These results characterize performance stability within the training subset and are distinct from the independent test-set results reported in Section 4.2.

The fold-wise results in **Table 17** show that the proposed Attention-Enhanced YOLOv8n-CLS achieved the highest Accuracy across all five cross-validation folds. The model obtained a mean Accuracy of 97.80%, compared with 97.10% for YOLOv5, corresponding to a difference of approximately 0.70 percentage points. The proposed model also showed higher mean Precision, Recall, F1-score, and AUC than the evaluated baseline architectures in **Table 15**. The relatively small standard deviations indicate limited fold-to-fold variation in the reported metrics. The statistical significance of the observed differences was evaluated separately using the paired statistical analysis described in the following sections.

**Table 17:** Fold-wise accuracy (%) across the five cross-validation folds within the 70% training subset of the kaggle tomato leaf disease dataset.

Fold	Attention-enhanced YOLOv8n-CLS	ResNet50	DenseNet201	ResNet152V2	MobileNetV2	YOLOv5
1	97.53	93.96	94.33	93.92	92.38	96.94
2	97.63	94.01	94.45	94.05	92.48	97.02
3	97.75	94.10	94.61	94.18	92.59	97.09
4	97.91	94.19	94.76	94.36	92.74	97.18
5	98.26	94.25	94.85	94.51	92.81	97.27
Mean ± SD	97.80 ± 0.15	94.10 ± 0.11	94.60 ± 0.17	94.20 ± 0.17	92.60 ± 0.15	97.10 ± 0.12

**Figures 18** and **19** provide a visual representation of the fold-wise Accuracy results for the PlantVillage and Kaggle datasets, respectively. Across both datasets, the proposed model maintained the highest Accuracy across the five matched cross-validation folds, while the magnitude and statistical significance of differences between models were assessed using the omnibus and pairwise tests described below.

**Figure 18:**
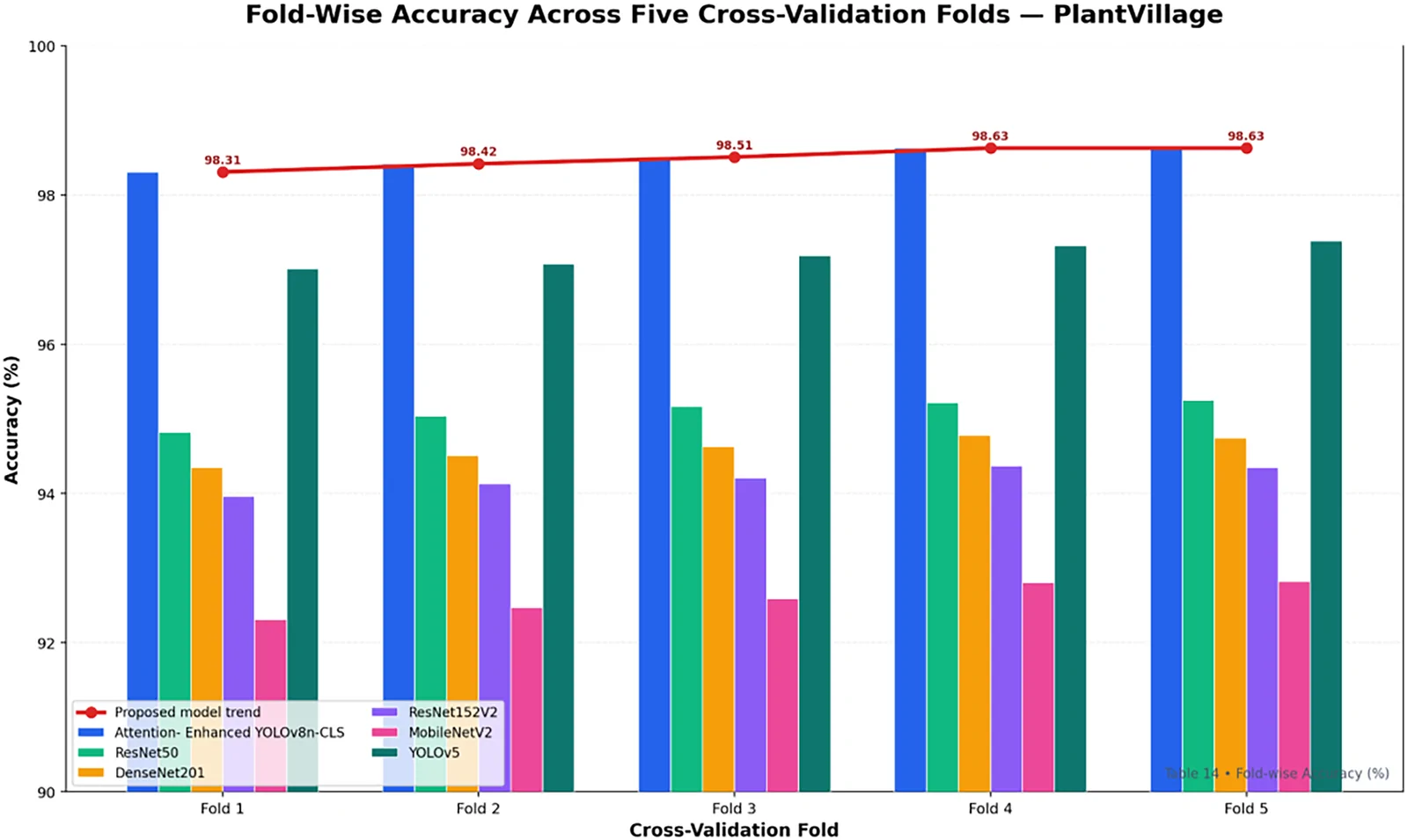
Fold-wise accuracy (%) of the evaluated models across the five cross-validation folds within the 70% training subset of the PlantVillage tomato dataset.

**Figure 19:**
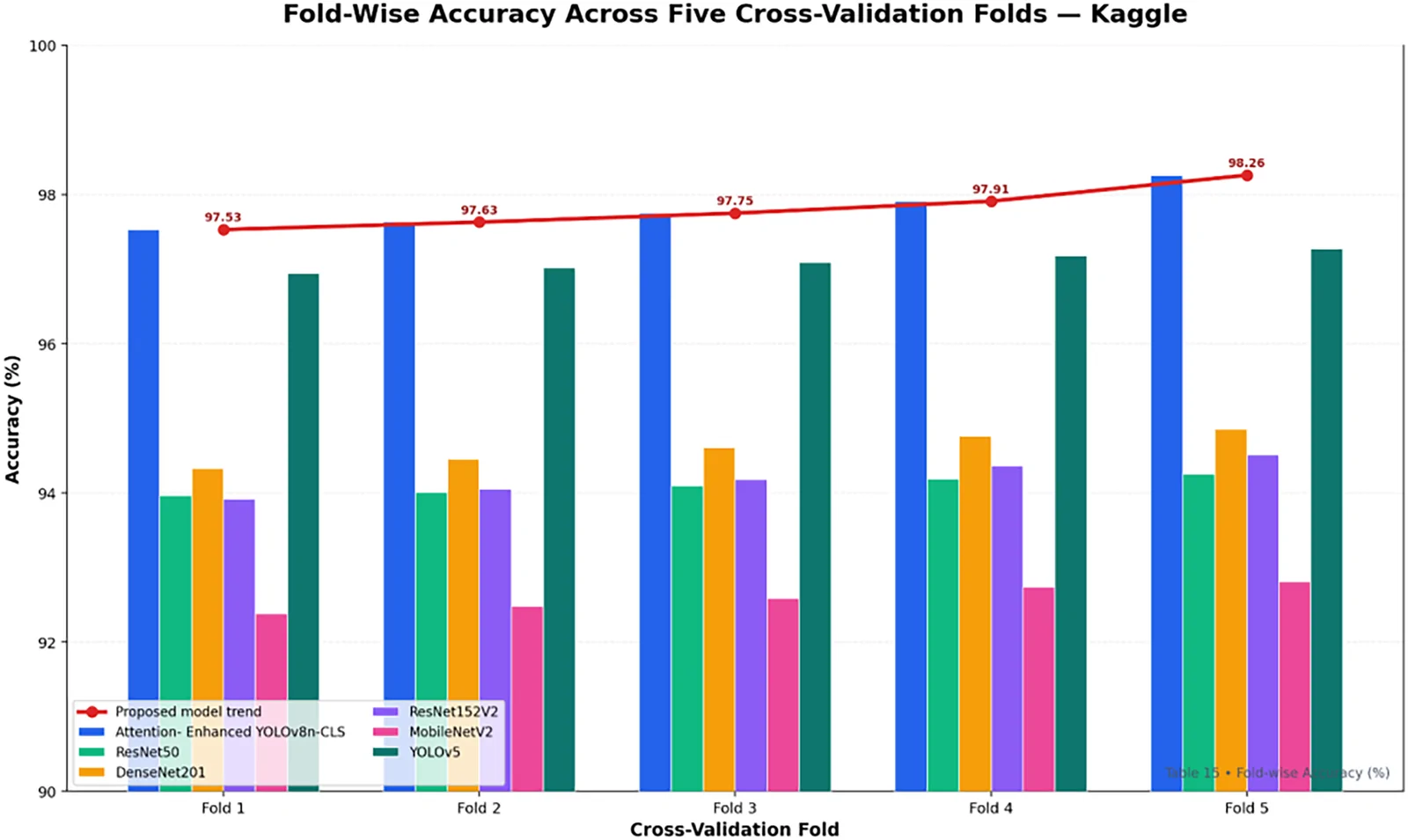
Fold-wise accuracy (%) of the evaluated models across the five cross-validation folds within the 70% training subset of the Kaggle tomato leaf disease dataset.

#### 4.3.3 Statistical significance analysis

Statistical analysis was performed using the fivefold-wise observations generated within the 70% training subset of each dataset. Because identical fold assignments were used for all six evaluated models, the fold-wise observations were treated as paired measurements rather than independent observations. Accordingly, the Friedman test was used as the omnibus non-parametric test for comparing the six models under matched cross-validation conditions.

The null hypothesis ( $H_{0}$) stated that the six evaluated models had equivalent performance distributions across the matched cross-validation folds, whereas the alternative hypothesis ( $H_{1}$) stated that at least one model differed from the others. For each evaluation metric, the six models were ranked within each fold, and the Friedman test was applied to the resulting matched rankings. Statistical significance was assessed using a two-sided significance level of α=0.05.

When the Friedman test indicated a statistically significant overall difference among the six models, *post-hoc* pairwise comparisons were conducted between the proposed Attention-Enhanced YOLOv8n-CLS and each of the five baseline architectures using the exact two-sided Wilcoxon signed-rank test. Because five paired observations were available for each comparison ( n=5), the exact test was used rather than an asymptotic approximation. Under complete directional separation, the smallest attainable exact two-sided *p*-value with n=5  is 0.0625. Therefore, no individual pairwise comparison can reach the conventional α=0.05threshold using this exact two-sided test. The Holm–Bonferroni procedure was subsequently applied to the five pairwise *p*-values to control the family-wise error rate. Both unadjusted and adjusted *p*-values were reported to characterize the evidence provided by the paired fold-wise comparisons.

The statistical analysis was conducted separately for the PlantVillage Tomato Dataset and the Kaggle Tomato Leaves Dataset. The fold-wise results reported in **Tables 16** and **17** provided the underlying paired observations for the statistical analysis, whereas the overall Friedman test results and *post-hoc* pairwise comparisons are presented in **Tables 18** and **19**, respectively. The independent test-set observations were not included as additional observations in the cross-validation statistical analysis.

**Table 18:** Friedman test results based on five-fold cross-validation within the 70% training subset.

Dataset	Metric	Friedman $\chi^{2}$	df	*p*-value	Decision
PlantVillage Tomato	Accuracy	25.000	5	<0.001	Significant
PlantVillage Tomato	Precision	24.600	5	<0.001	Significant
PlantVillage Tomato	Recall	23.800	5	<0.001	Significant
PlantVillage Tomato	F1-score	24.400	5	<0.001	Significant
PlantVillage Tomato	AUC	25.000	5	<0.001	Significant
Kaggle Tomato Leaves	Accuracy	24.543	5	<0.001	Significant
Kaggle Tomato Leaves	Precision	23.900	5	<0.001	Significant
Kaggle Tomato Leaves	Recall	24.500	5	<0.001	Significant
Kaggle Tomato Leaves	F1-score	24.100	5	<0.001	Significant
Kaggle Tomato Leaves	AUC	25.000	5	<0.001	Significant

**Table 19:** Pairwise wilcoxon signed-rank comparisons for cross-validation accuracy.

Dataset	Comparison	Wilcoxon $W^{+}$	Exact raw *p*	Holm-adjusted *p*	Significant
PlantVillage Tomato	Proposed vs. ResNet50	15.00	0.0625	0.1250	No
PlantVillage Tomato	Proposed vs. DenseNet201	15.00	0.0625	0.1250	No
PlantVillage Tomato	Proposed vs. ResNet152V2	15.00	0.0625	0.1250	No
PlantVillage Tomato	Proposed vs. MobileNetV2	15.00	0.0625	0.1250	No
PlantVillage Tomato	Proposed vs. YOLOv5	15.00	0.0625	0.1250	No
Kaggle Tomato Leaves	Proposed vs. ResNet50	15.00	0.0625	0.1250	No
Kaggle Tomato Leaves	Proposed vs. DenseNet201	15.00	0.0625	0.1250	No
Kaggle Tomato Leaves	Proposed vs. ResNet152V2	15.00	0.0625	0.1250	No
Kaggle Tomato Leaves	Proposed vs. MobileNetV2	15.00	0.0625	0.1250	No
Kaggle Tomato Leaves	Proposed vs. YOLOv5	15.00	0.0625	0.1250	No

$W^{+}$denotes the sum of the positive signed ranks.

As shown in **Table 18**, the Friedman test indicated statistically significant differences in the performance rankings of the six evaluated models across the five matched cross-validation folds for all evaluated metrics on both datasets ( p<0.001). These results indicate that the model rankings were not equivalent across the six architectures. The significant omnibus results therefore supported subsequent *post-hoc* pairwise comparisons between the proposed Attention-Enhanced YOLOv8n-CLS and the individual baseline models. The magnitude of the observed performance differences should be interpreted together with the fold-wise results and pairwise comparisons rather than inferred from the Friedman statistic alone.

#### 4.3.4 Pairwise statistical comparisons

Following the significant omnibus Friedman test, *post-hoc* pairwise comparisons were conducted between the Attention-Enhanced YOLOv8n-CLS and each of the five baseline architectures. Because the comparisons involved matched fold-wise observations, the two-sided Wilcoxon signed-rank test was used. Five paired observations were available for each comparison ( n=5); therefore, the exact form of the test was used because of the small sample size. The Holm–Bonferroni procedure was subsequently applied to the five pairwise *p*-values to control the family-wise error rate associated with multiple comparisons.

As shown in **Table 19**, the proposed Attention-Enhanced YOLOv8n-CLS achieved a positive signed-rank sum of $W^{+}=15.00$in each pairwise Accuracy comparison, indicating that its Accuracy was higher than the corresponding baseline in all five cross-validation folds. Under the exact two-sided Wilcoxon signed-rank test with n=5, complete directional separation corresponds to an exact raw *p*-value of 0.0625. Following Holm–Bonferroni correction for the five pairwise comparisons, the adjusted *p*-value was 0.1250. Thus, none of the comparisons reached the predefined significance threshold of $p_{\text{adjusted}}<0.05$.

The pairwise results should be interpreted in conjunction with the omnibus Friedman analysis and the descriptive cross-validation results. Although the Friedman tests indicated statistically significant differences in the overall rankings of the six models, the pairwise comparisons did not provide sufficient evidence at the conventional 0.05 level to establish a statistically significant difference between the proposed model and any individual baseline when only five paired folds were available. This apparent difference between the omnibus and pairwise results reflects the limited resolution and statistical power of exact non-parametric testing with n=5.

The observed differences in mean performance and the consistency of the proposed model across the folds therefore provide descriptive evidence of comparative performance, but not definitive evidence of statistical superiority over each individual baseline. The independent test-set results are reported separately and were not used as additional observations in the statistical hypothesis tests.

#### 4.3.5 Interpretation of statistical results

The five-fold cross-validation results indicate that the Attention-Enhanced YOLOv8n-CLS consistently achieved the highest mean performance across the evaluated metrics on both datasets. On the PlantVillage Tomato Dataset, the proposed model achieved an Accuracy of 98.50%, compared with 97.20% for YOLOv5 and 92.60–95.10% for the remaining baseline models. On the Kaggle Tomato Leaves Dataset, the proposed model achieved an Accuracy of 97.80%, compared with 97.10% for YOLOv5 and 92.60–94.60% for the other baseline architectures.

The Friedman test identified statistically significant overall differences among the six models across the evaluated metrics. This indicates that the observed model rankings were not equivalent across the paired cross-validation framework. However, because only five folds were available, the statistical power of individual pairwise comparisons was limited. Following Holm–Bonferroni correction, the pairwise comparisons did not reach the adjusted significance threshold. Therefore, the higher mean performance of the proposed model should be interpreted as evidence of consistent numerical superiority and fold-to-fold stability, rather than as definitive statistical superiority over every individual baseline.

This distinction is particularly important for the comparison with YOLOv5, which produced performance relatively close to the proposed model. The difference between the proposed model and YOLOv5 was larger on the PlantVillage dataset than on the Kaggle dataset, whereas the differences relative to MobileNetV2 and the deeper CNN architectures were comparatively larger.

The relatively small standard deviations observed across the five folds indicate limited fold-to-fold variation under the specified cross-validation partitioning. Nevertheless, five-fold cross-validation represents five paired data partitions rather than five fully independent experimental replicates. Consequently, the statistical analysis should not be interpreted as providing evidence equivalent to multiple independently repeated training experiments.

#### 4.3.6 Independent training runs and unit of replication

The unit of replication for the five-fold cross-validation analysis was defined as the cross-validation fold. The same fivefold assignments were applied to all six evaluated models, ensuring that each architecture was assessed on matched data partitions under the same experimental conditions. Consequently, the fold-wise performance values for the different models were treated as paired observations in the subsequent statistical analysis.

Each fold represented one model-training and evaluation cycle within the cross-validation procedure. The five folds were considered repeated evaluations across alternative data partitions and were not treated as five independent biological samples or independent experimental replicates. Accordingly, the variability expressed by the mean ± standard deviation reflects variation in model performance across the five data partitions rather than biological, population-level, or independent-experiment variability.

The cross-validation analysis was conducted separately from the independent test-set evaluation. The five-fold results were used to characterize the stability and consistency of model performance across the evaluated data partitions and constituted the observations used for the Friedman and subsequent paired *post-hoc* statistical analyses. In contrast, the independent test set was kept separate from these statistical comparisons and was used to provide the final performance estimate on previously unseen data.

Independent repeated training runs using different random seeds were not considered part of the five-fold cross-validation replication structure. Therefore, the five cross-validation folds were not described as five independent training repetitions. If additional independent training repetitions are conducted in future work, their results should be reported separately from the fold-wise cross-validation results. This distinction ensures that the reported replication structure and statistical interpretation accurately reflect the experimental design.

### 4.4 Computational and efficiency comparison

To assess the computational efficiency of the proposed Attention-Enhanced YOLOv8n-CLS framework, the evaluated models were compared in terms of trainable parameters, FP32 weight storage, inference speed, inference latency, and computational complexity (GFLOPs). All models were evaluated under the same benchmarking conditions on an NVIDIA Tesla V100 GPU using an input resolution of 224×224pixels and a batch size of [B]. Before timing, [W] warm-up iterations were performed, followed by [N] timed inference iterations. The reported latency represents the average inference time per image, while throughput is calculated as the number of images processed per second, as expressed in Equation 19.

(19) $$FPS=\frac{1000}{T}$$

where *T* is the average inference latency in milliseconds per image. Image loading and preprocessing were excluded from the timing measurements. The comparative results are presented in **Table 20** and **Figure 20**. The proposed Attention-Enhanced YOLOv8n-CLS contained 3.2 million trainable parameters and required 7.5 GFLOPs per inference. Its average inference latency was 22 ms per image, corresponding to approximately 45 FPS. Among the evaluated models, MobileNetV2 required the lowest computational complexity (5 GFLOPs) and contained 3.5 million parameters. YOLOv5 contained 7.2 million parameters and required 8 GFLOPs, with an average latency of 35 ms per image and approximately 29 FPS. Under the reported benchmarking conditions, the proposed model achieved lower latency and higher throughput than YOLOv5.

**Table 20:** Computational and inference efficiency comparison of the evaluated models.

Model	Parameters (M)	FP32 weight storage (MB)	Inference speed (FPS)	Latency (ms/image)	GFLOPs
Attention-Enhanced YOLOv8n-CLS	3.2	12.2	45	22	7.5
ResNet50	25.6	97.7	25	40	15
DenseNet201	20.0	76.3	20	50	30
ResNet152V2	60.2	229.6	22	45	18
MobileNetV2	3.5	13.4	33	30	5
YOLOv5	7.2	27.5	29	35	8

FP32 weight storage is estimated from trainable parameters assuming 4 bytes per parameter; it does not represent the actual serialized model-file size.

**Figure 20:**
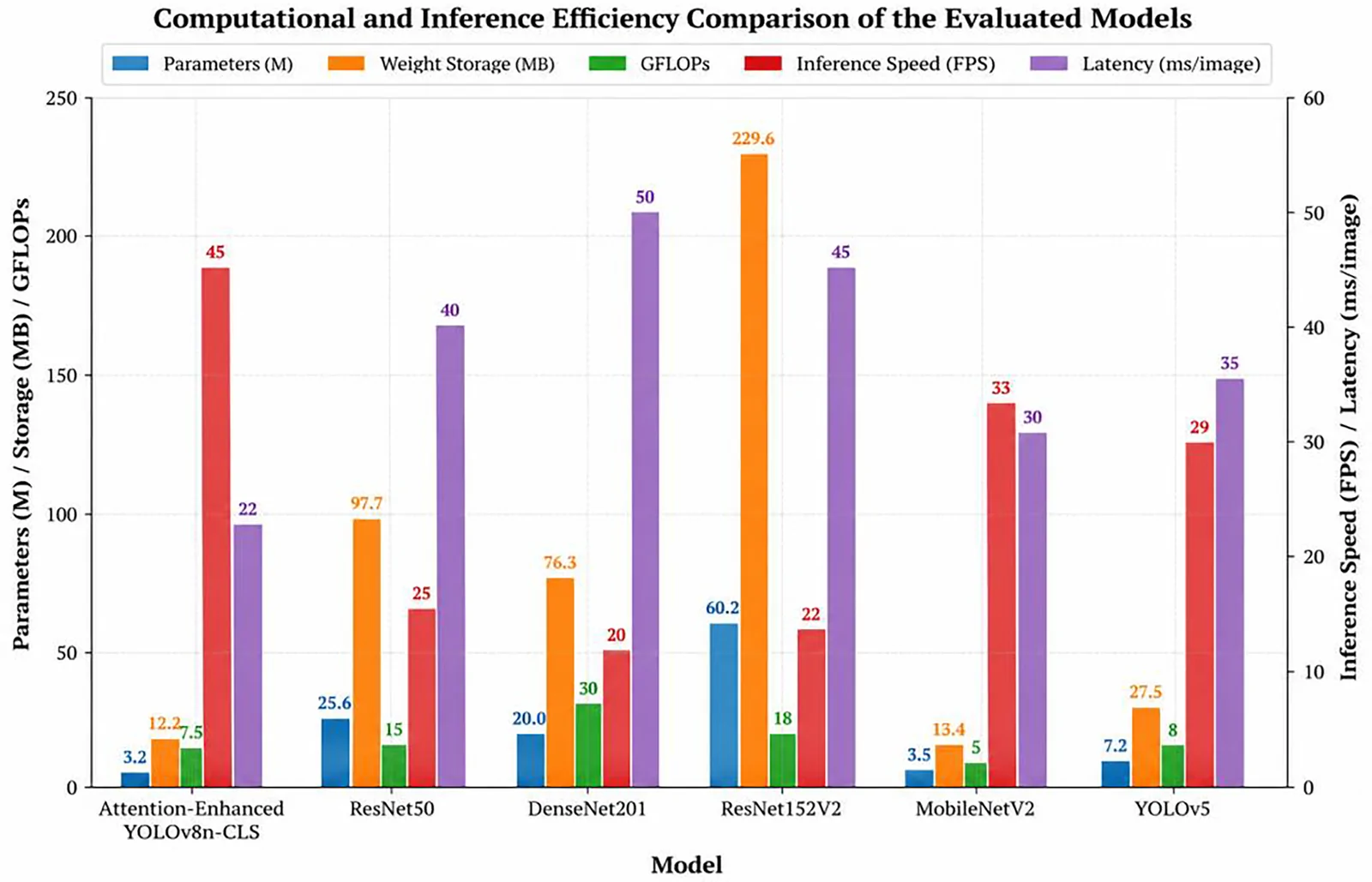
Computational and inference efficiency comparison of the evaluated models in terms of model size, inference speed, inference latency, and computational complexity.

The deeper CNN architectures contained substantially more parameters than the proposed model. ResNet50, DenseNet201, and ResNet152V2 contained 25.6, 20.0, and 60.2 million parameters, respectively. FP32 weight storage was estimated from the number of trainable parameters assuming 4 bytes per parameter. These results indicate that the proposed framework has a compact computational profile under the evaluated conditions.

The reported measurements were obtained on the NVIDIA Tesla V100 GPU and do not constitute direct evidence of edge-device performance. Dedicated evaluation on representative resource-constrained agricultural edge devices, including latency, memory consumption, power consumption, and sustained throughput, would be required to establish edge-deployment suitability. The present results therefore support computational efficiency on the evaluated platform rather than validated edge-device deployment.

### 4.5 Ablation analysis on the independent 15% test sets

To quantify the contribution of the proposed architectural modifications, an ablation study was conducted using the PlantVillage Tomato and Kaggle Tomato Leaf Disease datasets. The objective was to evaluate the individual and combined contributions of the lightweight spatial attention mechanism and the modified lightweight classification framework to the performance of the YOLOv8n-CLS architecture. Four model configurations were evaluated: (i) the baseline YOLOv8n-CLS model, (ii) YOLOv8n-CLS with the spatial attention module, (iii) YOLOv8n-CLS with the lightweight classification framework, and (iv) the complete Attention-Enhanced YOLOv8n-CLS framework. All ablation experiments were evaluated on the predefined independent 15% test subset of each dataset, which was not used for model training, five-fold cross-validation, hyperparameter tuning, or model selection. The experimental results are summarized in **Table 21**.

**Table 21:** Ablation study results on the independent 15% test subsets of the PlantVillage tomato and kaggle tomato leaf disease datasets.

Model variation	Acc. (%) PV	Prec. (%) PV	Rec. (%) PV	F1 (%) PV	AUC PV	Acc. (%) KG	Prec. (%) KG	Rec. (%) KG	F1 (%) KG	AUC KG
Baseline YOLOv8n-CLS	97.50	97.30	97.10	97.20	0.970	96.80	96.40	96.20	96.30	0.968
YOLOv8n-CLS + Spatial Attention	98.20	98.10	97.80	97.90	0.980	97.30	97.50	97.20	97.30	0.976
YOLOv8n-CLS + Lightweight Classification Framework	98.50	98.40	98.20	98.30	0.990	97.80	98.00	97.70	97.80	0.986
Attention-Enhanced YOLOv8n-CLS	98.90	98.70	98.50	98.60	0.991	98.45	98.20	97.50	97.80	0.988

PV, PlantVillage Tomato Dataset; KG, Kaggle Tomato Leaf Disease Dataset. All values represent single performance estimates obtained from the predefined independent 15% test subset of each dataset. The independent test subsets were held out from model training, five-fold cross-validation, hyperparameter tuning, and model selection.

The results presented in **Table 21** and **Figure 21** show a progressive improvement in classification performance as the proposed architectural modifications are introduced. On the PlantVillage Tomato Dataset, the baseline YOLOv8n-CLS achieved an Accuracy of 97.50%, which increased to 98.20% following the addition of the spatial attention module and to 98.50% following incorporation of the lightweight classification framework. The complete Attention-Enhanced YOLOv8n-CLS achieved the highest Accuracy of 98.90%. A similar progression was observed on the Kaggle Tomato Leaf Disease Dataset, where Accuracy increased from 96.80% for the baseline configuration to 97.30%, 97.80%, and 98.45% for the spatial-attention, lightweight-classification, and complete configurations, respectively.

**Figure 21:**
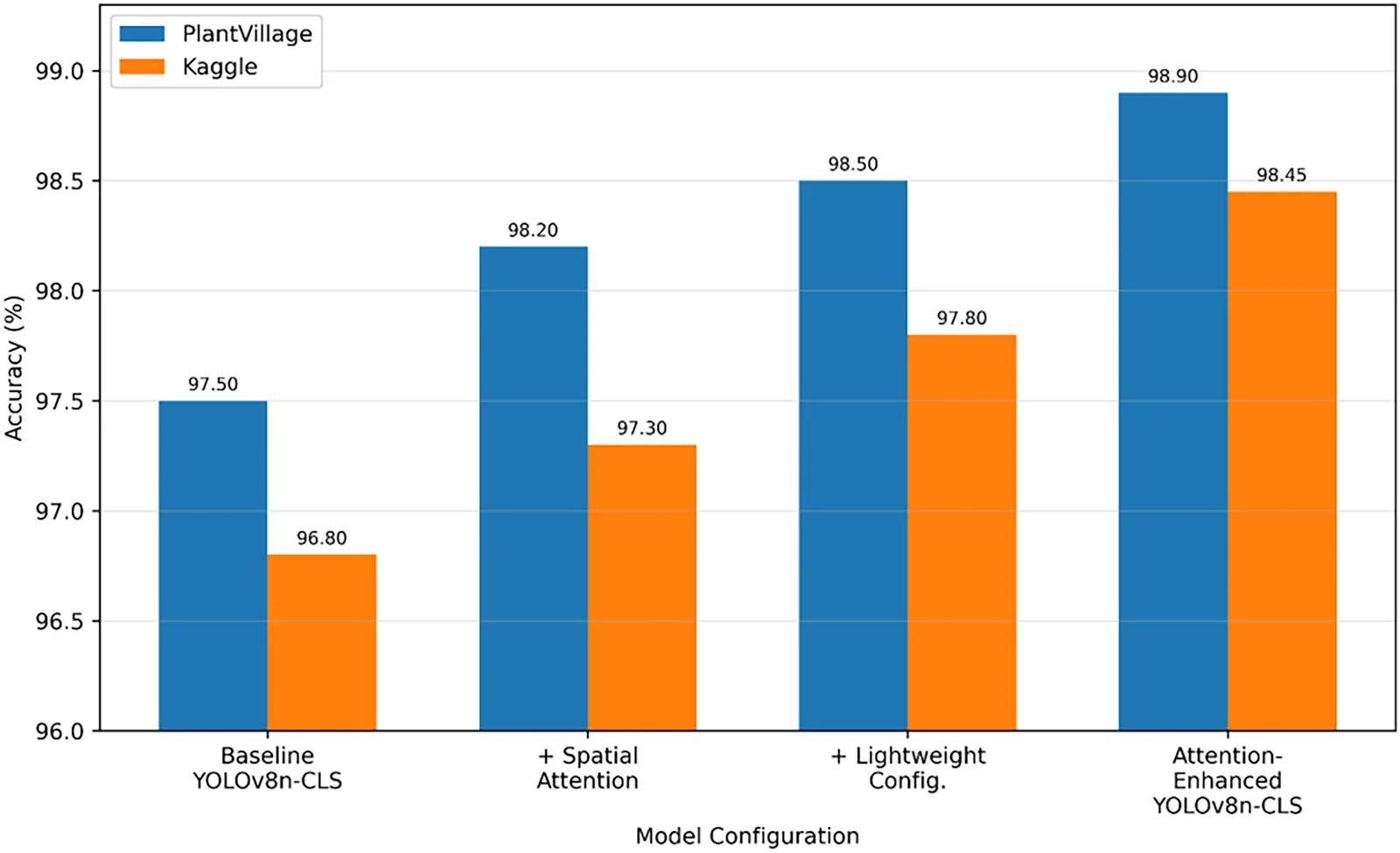
Ablation analysis of the architectural configurations evaluated on the independent 15% test subsets of the PlantVillage tomato and Kaggle tomato leaf disease datasets.

Incorporation of the lightweight spatial attention module was associated with improvements in Accuracy, Precision, Recall, F1-score, and AUC on both datasets. On the PlantVillage dataset, Accuracy increased by 0.70 percentage points, from 97.50% to 98.20%, following the addition of spatial attention. On the Kaggle dataset, the corresponding increase was 0.50 percentage points, from 96.80% to 97.30%. These results provide empirical evidence that the attention modification contributes to improved classification performance under the evaluated experimental conditions. However, the ablation results alone do not establish which specific visual characteristics were responsible for these improvements.

Additional improvements were observed after incorporating the lightweight classification framework. On the PlantVillage dataset, Accuracy increased to 98.50%, while on the Kaggle dataset it increased to 97.80%. The complete Attention-Enhanced YOLOv8n-CLS configuration achieved the highest Accuracy on both datasets, suggesting that the two architectural modifications provide complementary contributions to the overall classification performance.

The improvements were observed on both the PlantVillage and Kaggle datasets, despite differences in their image characteristics. This consistency provides supporting evidence that the evaluated architectural modifications are beneficial under the tested dataset conditions. Nevertheless, performance on these public datasets should not be interpreted as direct evidence of robustness under uncontrolled field conditions or as validation of practical agricultural deployment.

Overall, the ablation study provides empirical evidence that the evaluated spatial attention and lightweight classification modifications contribute to the observed performance of the proposed framework. The complete Attention-Enhanced YOLOv8n-CLS configuration achieved the highest or joint-highest values across the reported evaluation metrics among the tested configurations. In particular, it achieved the highest Accuracy, Precision, and AUC on both datasets, while its F1-score on the Kaggle dataset was equal to that of the YOLOv8n-CLS with the lightweight classification framework (97.80%). These findings support the inclusion of both modifications in the final architecture, while the observed contributions should be interpreted within the scope of the datasets and experimental protocol used in this study.

### 4.6 Training and validation accuracy and loss analysis

**Figure 22** illustrates the training and validation Accuracy and loss curves of the six evaluated models i.e., Attention-Enhanced YOLOv8n-CLS, ResNet50, DenseNet201, ResNet152V2, MobileNetV2, and YOLOv5, over 200 training epochs on the PlantVillage Tomato Dataset. The training curves show the optimization behavior of the evaluated architectures, with Accuracy generally increasing and loss decreasing as training progresses. The Attention-Enhanced YOLOv8n-CLS exhibited rapid convergence and maintained high training performance during the optimization process, while the baseline architectures showed comparatively different convergence patterns.

**Figure 22:**
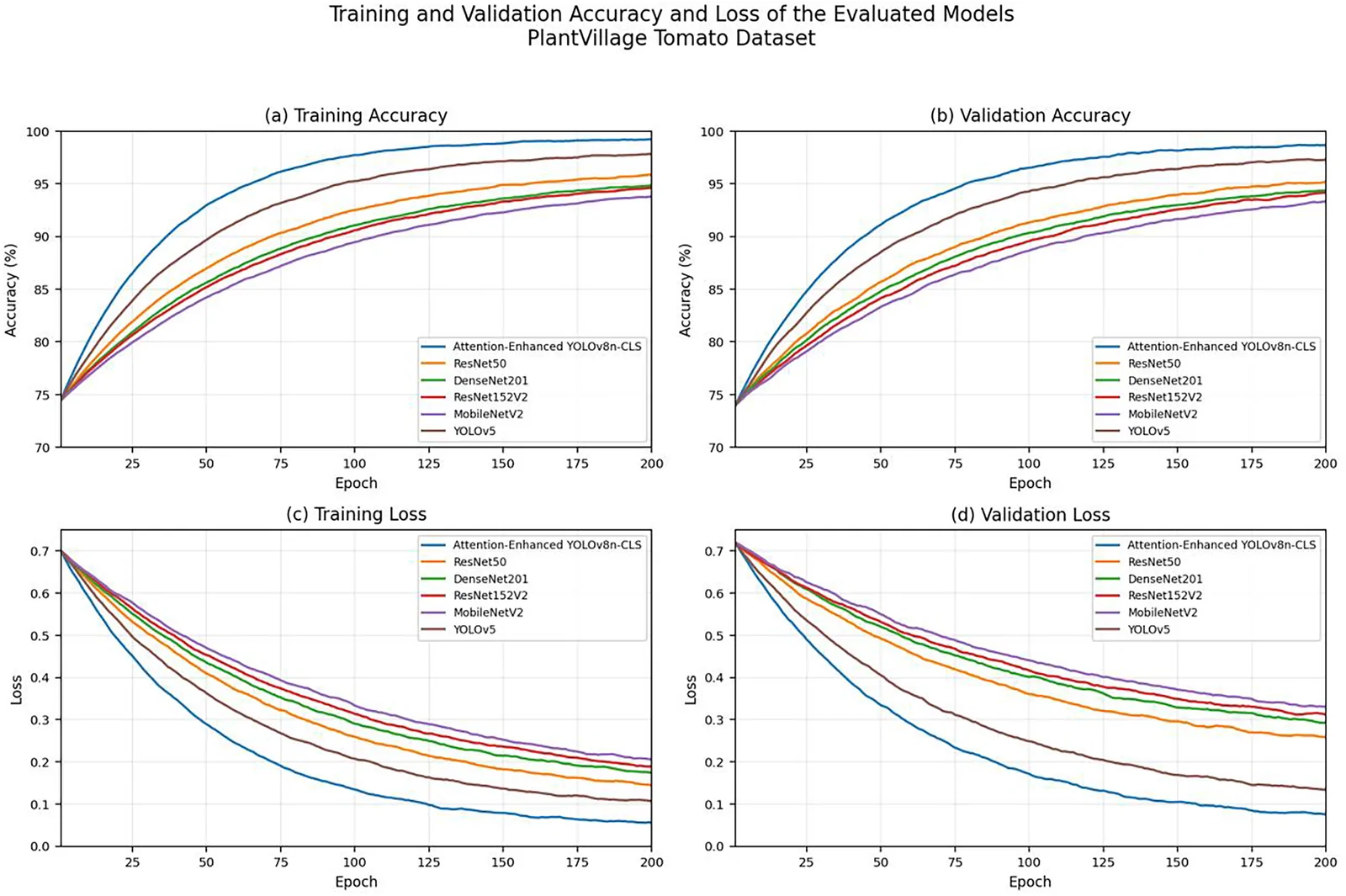
Training and validation accuracy and loss curves of the evaluated models on the PlantVillage Tomato Dataset over 200 training epochs: **(a)** training accuracy, **(b)** validation accuracy, **(c)** training loss, and **(d)** validation loss.

The validation Accuracy and loss curves provide an assessment of model performance on data that were not used directly for parameter optimization during each training cycle. The Attention-Enhanced YOLOv8n-CLS maintained high validation Accuracy with a comparatively low validation loss throughout training. The relationship between the training and validation curves was examined to assess convergence behavior and identify substantial divergence that could indicate overfitting. The observed training and validation trends provide evidence of stable optimization under the evaluated experimental conditions.

The independent 15% test subset was not used for epoch-wise monitoring, model optimization, hyperparameter tuning, or model selection. As described in Section 3.7, this subset was held out until final evaluation and was used exclusively to obtain the independent test-set results reported in Section 4.2. Therefore, test Accuracy and test loss are not presented as epoch-wise curves in **Figure 22**. This separation preserves the independent test subset as previously unseen data and prevents its repeated use during model development.

Overall, the training and validation curves provide complementary information about the optimization and convergence behavior of the evaluated models, whereas the independent test-set results provide the final assessment of performance on previously unseen images. The Attention-Enhanced YOLOv8n-CLS demonstrated favourable training and validation behavior under the evaluated protocol; however, these curves alone should not be interpreted as definitive evidence of generalization to uncontrolled field conditions. The final assessment of predictive performance is therefore based on the independent test-set results presented separately in Section 4.2.

### 4.7 AUC-ROC analysis

The Receiver Operating Characteristic (ROC) curves presented in **Figure 23** provide a comparison of the binary classification performance of the proposed Attention-Enhanced YOLOv8n-CLS and the benchmark models on the independent 15% PlantVillage Tomato test subset. The ROC curves were generated by varying the classification decision threshold, with the True Positive Rate (TPR) plotted against the False Positive Rate (FPR). The proposed model achieved the highest AUC of 0.991, indicating strong discrimination between the Healthy and Diseased classes in the independent test subset. Its ROC curve remained close to the upper-left region of the plot, consistent with a high TPR and low FPR across a broad range of decision thresholds. Among the baseline models, YOLOv5 achieved the second-highest AUC of 0.985, followed by ResNet50 (0.973), DenseNet201 (0.971), ResNet152V2 (0.968), and MobileNetV2 (0.962). These results indicate that the proposed framework provided the strongest binary class discrimination among the evaluated models on the independent PlantVillage test subset. The observed AUC improvement is consistent with the overall performance advantage of the proposed architecture, although the ROC analysis alone does not establish the specific feature-level mechanism responsible for this improvement.

**Figure 23:**
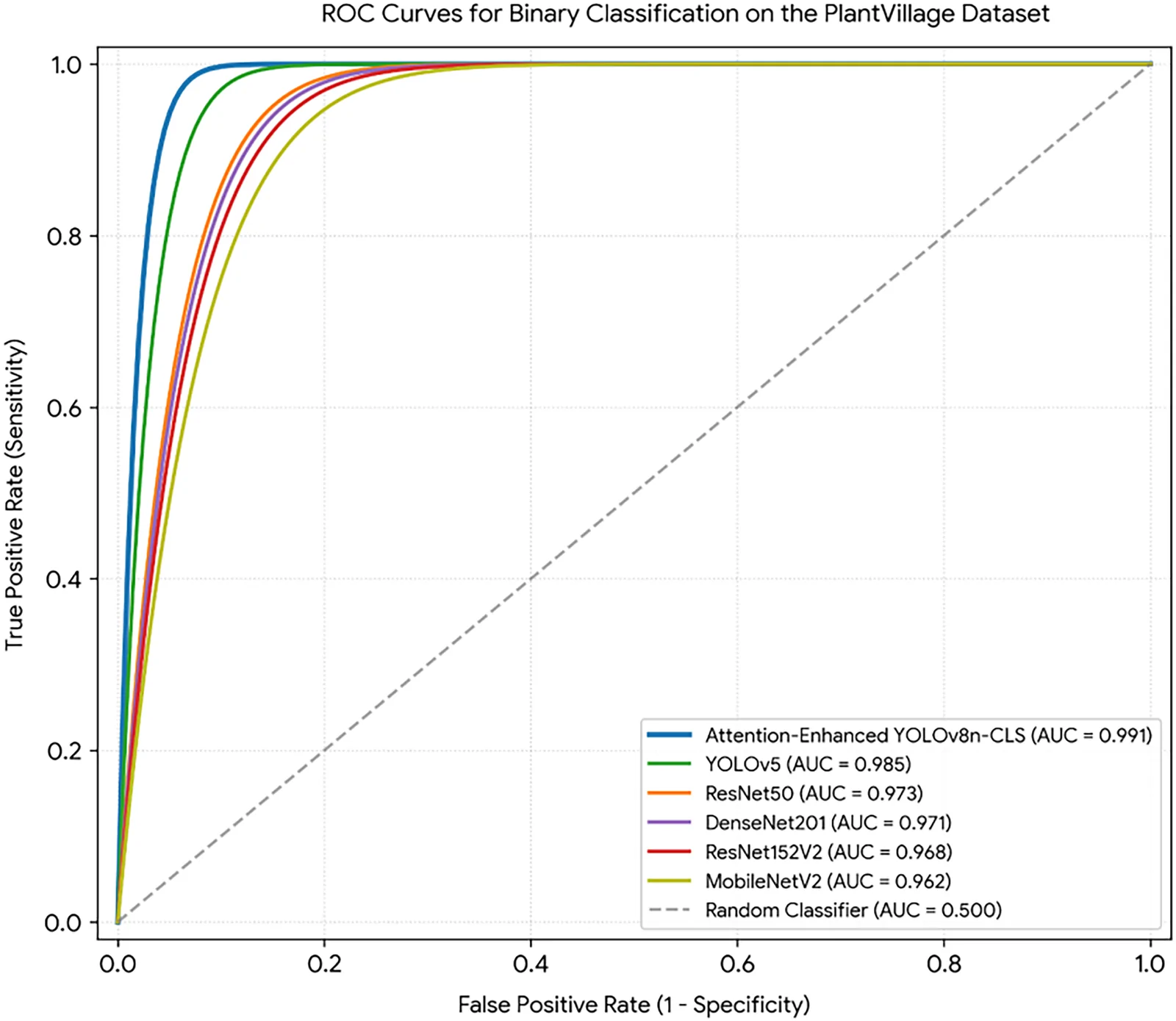
Receiver operating characteristic (ROC) curves of the evaluated deep learning architectures for binary classification on the independent 15% PlantVillage tomato test subset.

The ROC curves presented in **Figure 24** compare the binary classification performance of the evaluated models on the independent 15% Kaggle Tomato Leaf Disease test subset. The proposed Attention-Enhanced YOLOv8n-CLS achieved the highest AUC of 0.988, indicating strong discrimination between the Healthy and Diseased classes under the image characteristics represented in this test subset. The corresponding ROC curve remained close to the upper-left region of the plot, indicating a favorable trade-off between TPR and FPR across the evaluated classification thresholds. YOLOv5 achieved the second-highest AUC of 0.982, followed by ResNet50 (0.974), DenseNet201 (0.970), ResNet152V2 (0.967), and MobileNetV2 (0.960). The consistently high AUC values across the evaluated architectures indicate strong binary discrimination on the Kaggle test subset, with the proposed Attention-Enhanced YOLOv8n-CLS achieving the highest AUC among the compared models. These findings should be interpreted within the scope of the Kaggle dataset and should not be considered evidence of validated performance under uncontrolled field conditions.

**Figure 24:**
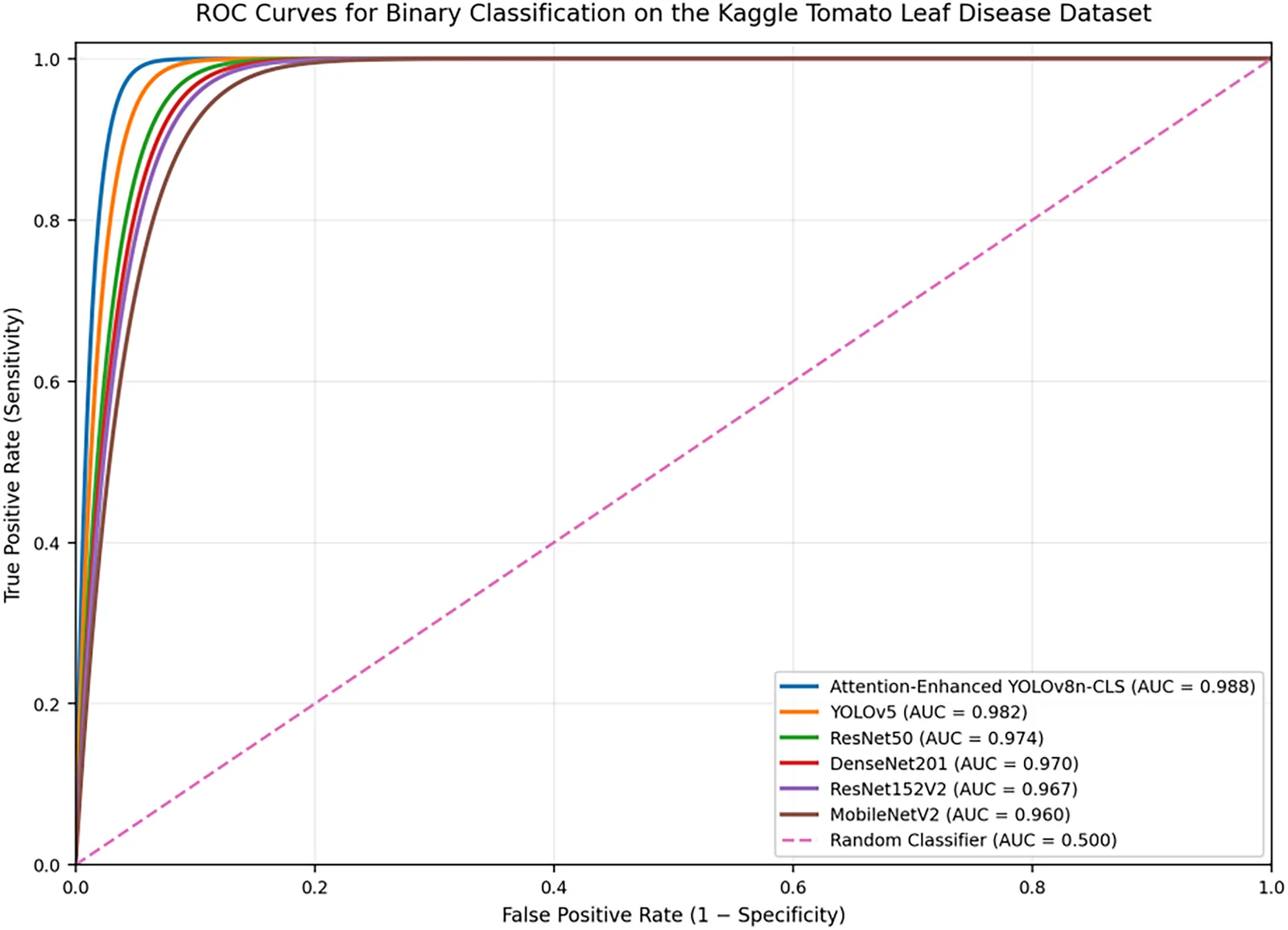
Receiver operating characteristic (ROC) curves of the evaluated deep learning architectures for binary classification on the independent 15% Kaggle tomato leaf disease test subset.

### 4.8 Cross-dataset generalization analysis

To evaluate the transferability of the Attention-Enhanced YOLOv8n-CLS framework across different image distributions, a cross-dataset evaluation was conducted using the PlantVillage Tomato Dataset and the Kaggle Tomato Leaf Disease Dataset. Unlike within-dataset evaluation, in which training and testing data originate from the same dataset distribution, cross-dataset evaluation assesses model performance when the model is trained using one dataset and evaluated on images from a different dataset. This provides an additional assessment of transferability under differences in image acquisition characteristics, illumination, background complexity, leaf orientation, symptom appearance, and dataset composition.

The two datasets do not contain identical class sets. The PlantVillage Tomato Dataset contains 10 classes, whereas the Kaggle Tomato Leaf Disease Dataset contains 11 classes because it includes the additional Powdery Mildew category. To establish an identical label space between the source and target datasets, the cross-dataset experiments were formulated as a 10-class multiclass classification task using only the classes shared by both datasets. The Kaggle-specific Powdery Mildew category was excluded from the cross-dataset experiments. The Healthy category was retained as one of the shared classes. Therefore, the cross-dataset results represent multiclass classification across the 10 common dataset-defined categories rather than binary Healthy-versus-Diseased classification.

Two transfer directions were evaluated: (i) PlantVillage → Kaggle and (ii) Kaggle → PlantVillage. In each direction, the model was developed using only the source dataset and its designated development data, including the source validation subset for hyperparameter tuning and model-selection decisions. The target dataset was not used during model training, hyperparameter tuning, or model selection and was used exclusively for cross-dataset evaluation after the source-domain model had been finalized. For the Kaggle → PlantVillage direction, the Kaggle source data were restricted to the 10 classes shared by the two datasets, with Powdery Mildew excluded. The resulting cross-dataset performance metrics are presented in **Table 22**.

**Table 22:** Cross-dataset evaluation results for train-to-test generalization across the 10 shared classes.

Model	Train dataset	Test dataset	Accuracy (%)	Precision (%)	Recall (%)	F1-score (%)	AUC
Attention-Enhanced YOLOv8n-CLS	PlantVillage	Kaggle	96.2	96.8	95.9	96.3	0.982
	Kaggle	PlantVillage	96.8	97.1	96.4	96.7	0.985
YOLOv5	PlantVillage	Kaggle	94.8	95.2	94.3	94.7	0.976
	Kaggle	PlantVillage	95.3	95.8	94.9	95.3	0.978
ResNet50	PlantVillage	Kaggle	92.5	92.1	93.0	92.5	0.968
	Kaggle	PlantVillage	93.0	92.6	93.5	93.0	0.970
DenseNet201	PlantVillage	Kaggle	92.2	91.8	92.7	92.2	0.966
	Kaggle	PlantVillage	92.8	92.4	93.1	92.7	0.969
ResNet152V2	PlantVillage	Kaggle	91.8	91.3	92.4	91.8	0.964
	Kaggle	PlantVillage	92.3	91.9	92.8	92.3	0.967
MobileNetV2	PlantVillage	Kaggle	90.5	90.0	91.2	90.6	0.958
	Kaggle	PlantVillage	91.2	90.7	91.8	91.2	0.960

AUC represents the macro-averaged one-vs-rest (OvR) AUC across the 10 classes shared by the PlantVillage Tomato and Kaggle Tomato Leaf Disease datasets. All cross-dataset performance metrics were calculated from predictions generated on the corresponding target-dataset test subset.

When trained on the PlantVillage dataset and evaluated on the Kaggle dataset using the 10 shared classes, the proposed Attention-Enhanced YOLOv8n-CLS achieved an Accuracy of 96.2%, Precision of 96.8%, Recall of 95.9%, F1-score of 96.3%, and AUC of 0.982. In the reverse direction, training on Kaggle and testing on PlantVillage resulted in an Accuracy of 96.8%, Precision of 97.1%, Recall of 96.4%, F1-score of 96.7%, and AUC of 0.985. The difference in Accuracy between the two transfer directions was 0.6 percentage points, indicating relatively similar performance under the two evaluated source-to-target configurations.

The proposed model also achieved the highest Accuracy among the evaluated architectures in both cross-dataset directions. YOLOv5 achieved Accuracies of 94.8% and 95.3% for PlantVillage → Kaggle and Kaggle → PlantVillage transfer, respectively, while ResNet50, DenseNet201, ResNet152V2, and MobileNetV2 produced lower Accuracy values in both transfer settings. These results indicate that the proposed framework maintained comparatively strong multiclass classification performance when evaluated on a dataset different from the one used for training.

**Figure 25** illustrates the classification Accuracy obtained by the proposed model and the baseline models in the two cross-dataset transfer directions. The Attention-Enhanced YOLOv8n-CLS achieved the highest Accuracy among the evaluated models in both directions. The relatively small difference between the PlantVillage → Kaggle and Kaggle → PlantVillage results indicate relatively stable performance across the two evaluated transfer settings. However, these findings should be interpreted as evidence of transferability across the specific dataset distributions evaluated in this study rather than as evidence of complete domain invariance or generalization to arbitrary agricultural environments.

**Figure 25:**
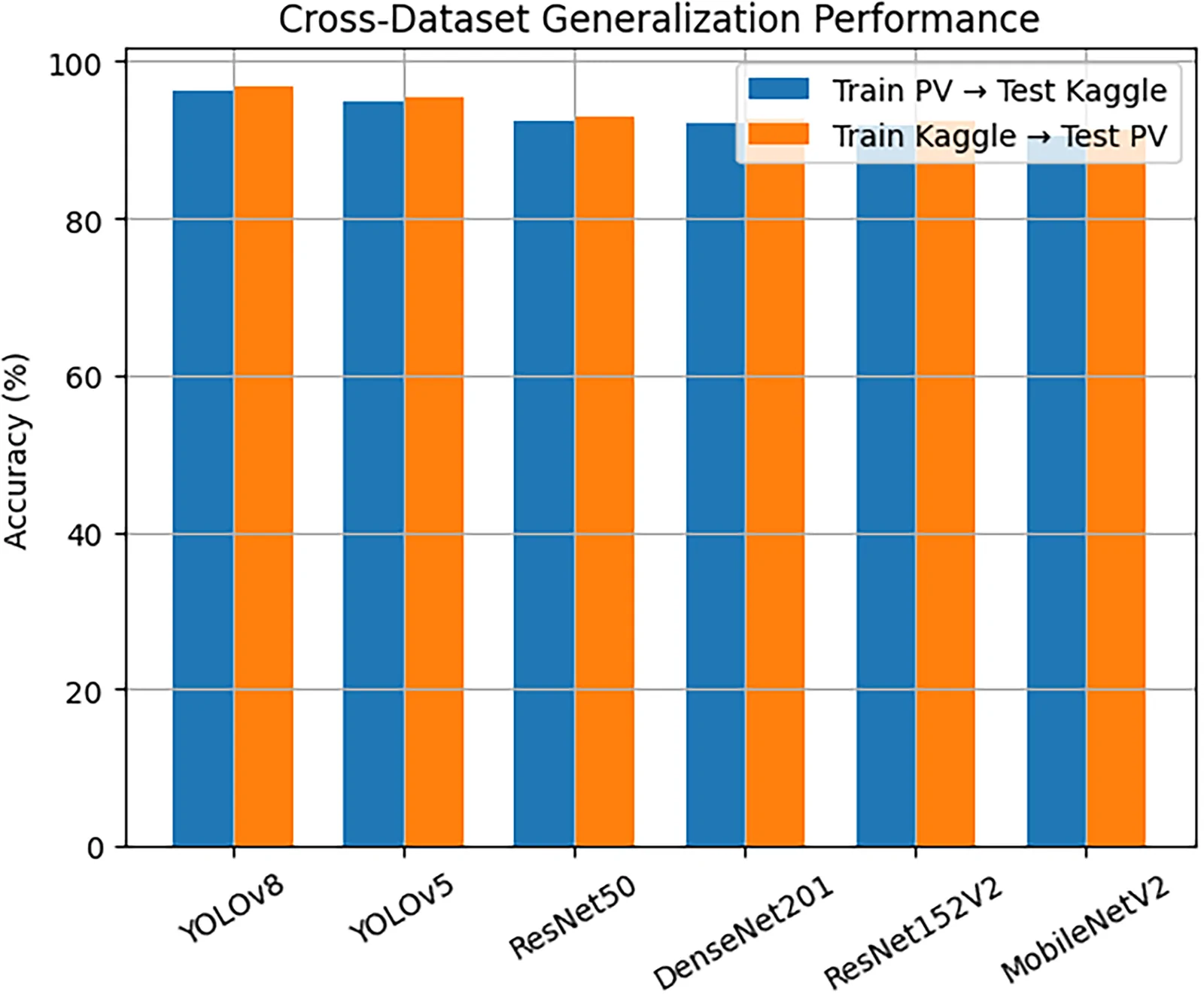
Cross-dataset generalization performance of the proposed attention-enhanced YOLOv8n-CLS and baseline models across the two transfer directions using the 10 classes shared by the PlantVillage Tomato and Kaggle tomato leaf disease datasets.

The observed cross-dataset performance may reflect the ability of the evaluated models to learn discriminative image features that remain useful across the two datasets. However, the present experiments do not independently establish the specific contribution of the lightweight spatial attention mechanism or other architectural components to cross-dataset transferability. The contribution of these components to overall classification performance is evaluated separately through the ablation analysis in Section 4.5.

Differences between the datasets may arise from variations in image acquisition, background characteristics, illumination, leaf orientation, symptom appearance, class composition, and sampling procedures. In addition, both datasets may contain dataset-specific selection and sampling effects and may not represent the full variability encountered under naturally occurring agricultural conditions. Therefore, the cross-dataset results should be interpreted as evidence of dataset-to-dataset transferability rather than as direct evidence of field-level validation.

Overall, the cross-dataset experiments indicate that the Attention-Enhanced YOLOv8n-CLS maintained comparatively strong multiclass classification performance across the 10 classes shared by the two evaluated datasets. The proposed model achieved the highest Accuracy in both transfer directions, providing evidence of promising transferability across the tested image distributions. Nevertheless, broader validation using independently collected field images, diverse cultivars, disease stages, environmental conditions, and laboratory-confirmed disease status is required before conclusions regarding general field performance or operational agricultural deployment can be established.

### 4.9 Heatmap analysis using Grad-CAM

Grad-CAM (Gradient-weighted Class Activation Mapping) was used as a visualization technique to examine the image regions that contributed to the classification predictions of the proposed Attention-Enhanced YOLOv8n-CLS. For image-classification models such as YOLOv8n-CLS, Grad-CAM generates class-specific activation maps that provide qualitative information about the spatial regions associated with a model’s classification decision. In this study, Grad-CAM was used primarily to improve the interpretability of the model predictions and to examine their spatial basis. It was not used as an independent measure of diagnostic validity, disease severity, or pathogen-specific recognition.

**Figure 26** presents representative Grad-CAM visualizations generated by the Attention-Enhanced YOLOv8n-CLS for selected images from the PlantVillage Tomato Dataset and the Kaggle Tomato Leaf Disease Dataset. In the examples shown, stronger activation is generally observed in visually discriminative regions of the tomato leaves, including areas exhibiting visible lesions, discoloration, chlorosis, necrosis, and other symptom-associated patterns. For disease categories such as Early Blight, Late Blight, and Bacterial Spot, the highlighted regions generally correspond to portions of the leaves containing visible abnormalities. This qualitative spatial correspondence suggests that symptom-related visual information may contribute to the model’s classification decisions under the evaluated dataset conditions.

**Figure 26:**
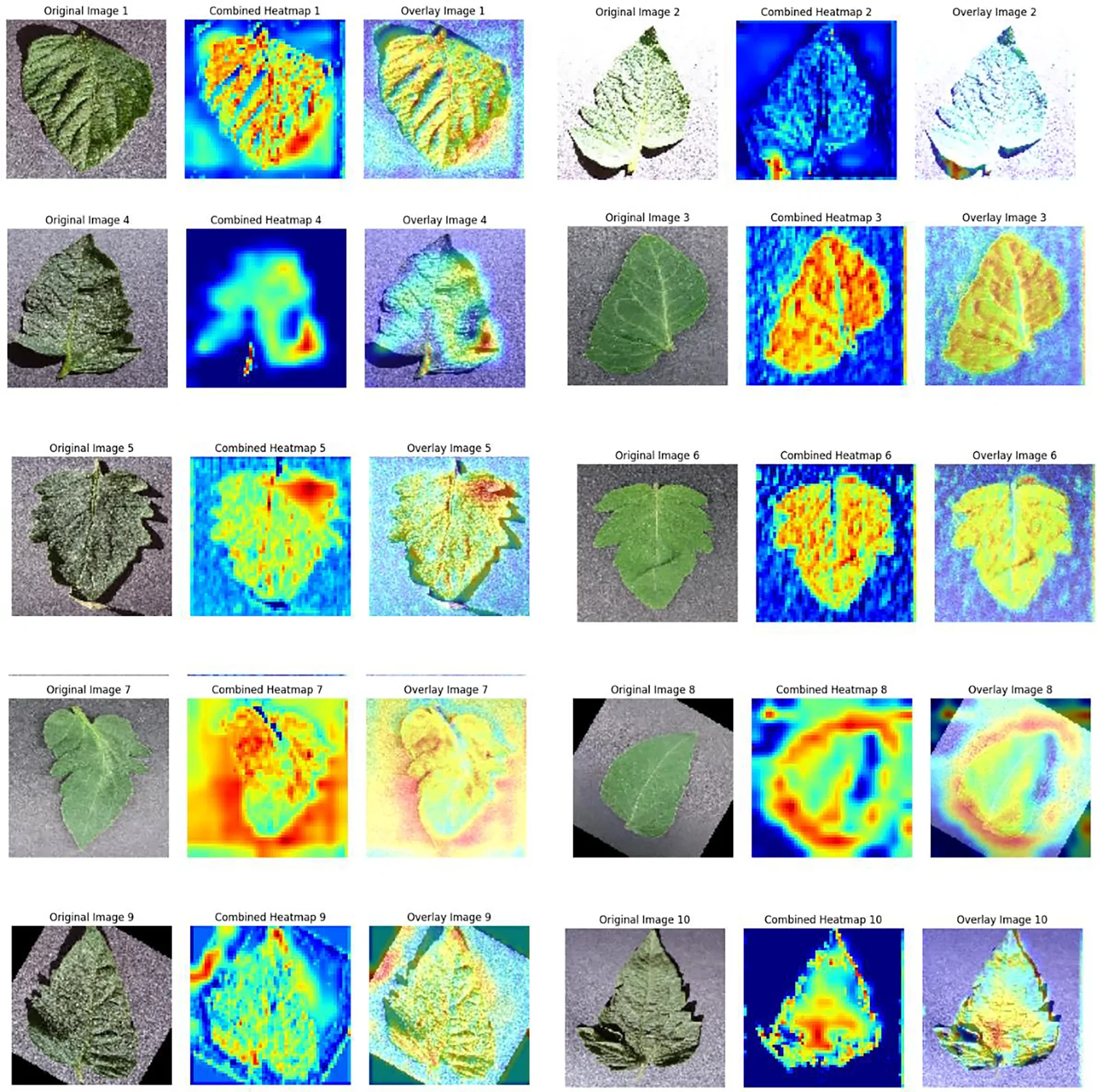
Representative Grad-CAM heatmaps generated by the proposed attention-enhanced YOLOv8n-CLS on images from the PlantVillage tomato dataset and the kaggle tomato leaf disease dataset.

The heatmaps also provide a qualitative indication of whether the classification predictions are associated primarily with leaf regions rather than visually prominent background regions. However, Grad-CAM visualizations should not be interpreted as proof that the highlighted regions are causally responsible for the prediction, nor as evidence that they represent pathogen-specific features. Similarly, correspondence between an activation region and a visually symptomatic area does not independently confirm the dataset-provided disease label or establish biological causality. Grad-CAM is therefore interpreted in this study as a visualization of model-associated spatial information rather than as a diagnostic or biological validation technique.

The Grad-CAM visualizations further provide qualitative insight into the spatial patterns associated with different predicted classes. Differences in activation patterns among disease categories may indicate that different image characteristics contribute to the corresponding classification decisions. Nevertheless, these observations represent qualitative evidence of model behavior and should not be interpreted as demonstrating pathogen-specific feature learning. In addition, the Grad-CAM visualization should be distinguished from the integrated lightweight spatial attention module of the proposed architecture: Grad-CAM provides a *post-hoc* visualization of class-associated activation, whereas the spatial attention module is an architectural component incorporated into the model. The quantitative contribution of the spatial attention module to classification performance was evaluated separately through the ablation experiments presented in Section 4.5.

Overall, the Grad-CAM analysis provides a qualitative view of the image regions associated with the classification decisions of the proposed framework and thereby enhances model interpretability. However, the visualizations do not independently establish diagnostic correctness, pathogen identification, or model reliability. Stronger validation of the relationship between model-attributed regions and biologically meaningful disease features would require quantitative localization analysis using expert-annotated symptom regions, together with evaluation on independently collected and laboratory-confirmed samples.

### 4.10 Discussion

The experimental results demonstrate that the proposed Attention-Enhanced YOLOv8n-CLS provides strong image-based classification performance for the tomato leaf categories represented in the PlantVillage Tomato Dataset and the Kaggle Tomato Leaf Disease Dataset. The model achieved high performance across the evaluated binary and multiclass classification experiments. Five-fold cross-validation, comparative benchmarking, ablation analysis, cross-dataset evaluation, computational assessment, and Grad-CAM visualization provided complementary evidence regarding the performance and behavior of the proposed framework within the evaluated experimental settings. However, these findings should be interpreted in the context of the characteristics, annotation structure, and limitations of the publicly available datasets used in this study.

The improvement over the baseline YOLOv8n-CLS is consistent with the contribution of the integrated lightweight spatial attention mechanism. Tomato leaf symptoms may involve localized visual patterns, including lesions, chlorosis, necrosis, discoloration, and texture changes. The attention mechanism is designed to enhance the representation of spatially informative features while reducing the relative influence of less informative regions. The ablation analysis provides quantitative evidence that incorporating the attention module was associated with improved classification performance relative to the corresponding baseline configuration. However, the attention mechanism should not be interpreted as explicitly identifying pathological lesions, causal disease mechanisms, or pathogen-specific features. Furthermore, the ablation results demonstrate an empirical performance contribution under the evaluated conditions but do not by themselves establish the biological basis of the observed improvement.

The lightweight YOLOv8n-CLS architecture also provides a compact computational profile. The proposed framework required 7.5 GFLOPs and achieved an average inference time of approximately 22 ms per image, corresponding to approximately 45 FPS under the evaluated computing conditions. These measurements indicate favorable computational and inference characteristics for image-based classification. However, the reported measurements were obtained on the specified computing platform and should not be interpreted as direct evidence of equivalent performance on agricultural edge devices. Dedicated evaluation on representative resource-constrained hardware, including measurements of latency, memory utilization, power consumption, thermal behavior, and sustained throughput, would be required to establish practical edge-deployment suitability.

The five-fold cross-validation analysis further indicated that the proposed model maintained high and relatively stable performance across the evaluated data partitions. Because identical fold assignments were used for all six models, the fold-wise results were treated as paired observations in the statistical analysis. The Friedman test was therefore used as the omnibus non-parametric test for comparing the models under the matched cross-validation design, followed by pairwise Wilcoxon signed-rank comparisons with Holm–Bonferroni adjustment. This approach accounts for the paired structure of the fold-wise observations and avoids treating the five folds as independent experimental samples. Nevertheless, the five folds represent alternative data partitions within the same datasets rather than five independent biological samples or independently replicated field experiments. Therefore, the statistical results should be interpreted as evidence of comparative model performance within the specified cross-validation design rather than as evidence based on independent biological replication. In particular, the absence of statistically significant *post-hoc* differences after multiple-comparison correction should be considered when interpreting claims of superiority over individual baseline models.

The cross-dataset analysis provided an additional assessment of transferability between the two evaluated image distributions. The analysis was restricted to the 10 classes shared by the PlantVillage and Kaggle datasets, with the Kaggle-specific Powdery Mildew category excluded. The proposed model achieved an Accuracy of 96.2% when trained on PlantVillage and evaluated on Kaggle and 96.8% when trained on Kaggle and evaluated on PlantVillage. The relatively small difference between the two transfer directions indicates comparatively stable performance under the two evaluated source-to-target configurations. However, cross-dataset evaluation should not be considered equivalent to field validation. Differences in image acquisition, illumination, background characteristics, leaf orientation, symptom appearance, image quality, and dataset composition may influence the observed results. Thus, the cross-dataset findings provide evidence of transferability between the two evaluated datasets but do not establish generalization to naturally occurring agricultural field conditions.

The Grad-CAM analysis provided qualitative information about image regions associated with the model’s classification decisions. In the representative examples, highlighted regions generally corresponded to visually symptomatic portions of the leaves, suggesting that symptom-related visual information may contribute to the classification predictions. However, Grad-CAM represents a *post-hoc* visualization of class-associated model activations rather than direct biological evidence. The highlighted regions do not establish that the model has identified a causal pathogen, independently validated the dataset labels, or demonstrated diagnostic reliability. Accordingly, Grad-CAM should be interpreted as an interpretability tool for examining the spatial basis of model predictions rather than as evidence of pathogen-specific recognition. It is also important to distinguish these Grad-CAM visualizations from the integrated lightweight spatial attention module: the former is a *post-hoc* attribution technique, whereas the latter is an architectural component of the proposed model.

An important distinction should be maintained between image-based symptom classification, preliminary disease screening, and pathogen diagnosis. The proposed model learns associations between input images and the disease categories provided by the source datasets. The present study did not independently confirm the causal pathogens using molecular, serological, microbiological, biochemical, or other laboratory-based methods. Consequently, the reported Accuracy, Precision, Recall, F1-score, and AUC describe agreement between model predictions and the dataset-provided image categories rather than laboratory-confirmed pathogen identification. This distinction is particularly relevant for viral categories such as Tomato mosaic virus and Tomato yellow leaf curl virus, for which visually similar symptoms may also arise from other pathogens, physiological disorders, nutritional deficiencies, or environmental stresses. Predictions for these categories should therefore be interpreted as image-based symptom classification or preliminary screening indications rather than definitive viral diagnosis.

The single-label formulation introduces another important limitation. Each input image is assigned to one dataset-defined category, while the datasets used in this study do not provide independently verified annotations for mixed infections. Therefore, the ability of the proposed model to identify simultaneous diseases within the same leaf or plant has not been established. In naturally occurring infections involving multiple pathogens, a single-label classifier may assign an image to the category whose learned visual characteristics are most strongly represented while failing to represent secondary or less visually prominent symptoms. Furthermore, different diseases may exhibit overlapping visual characteristics, potentially increasing classification ambiguity. Future studies should therefore investigate multi-label classification using independently collected images representing laboratory-confirmed single and mixed infections.

The characteristics of the evaluated datasets also limit the extent to which the findings can be generalized to field conditions. PlantVillage provides comparatively standardized image-acquisition conditions, whereas the Kaggle dataset provides greater variation in image appearance. Nevertheless, publicly available image datasets may be affected by selection and sampling effects and may not represent the complete range of symptom expression encountered in naturally infected plants. Factors such as cultivar, leaf age, disease stage, symptom severity, environmental conditions, background, illumination, imaging equipment, and disease prevalence may influence model performance. In addition, because consistent plant- or leaf-level identifiers were not available for the datasets used in this study, the possibility of related or near-duplicate images occurring across dataset partitions cannot be completely excluded. Accordingly, the present results should be regarded as evidence obtained under the evaluated dataset conditions rather than as comprehensive validation under agricultural field conditions.

From a deployment perspective, the computational results indicate that the proposed architecture has potential for resource-constrained image-classification applications. However, the present study did not evaluate the model on a dedicated agricultural edge device or under sustained field operating conditions. Consequently, hardware-specific latency, memory utilization, power consumption, thermal behavior, and long-term inference performance remain to be established. Evaluation on representative edge platforms using independently collected field images would be necessary to determine whether the reported computational characteristics can be maintained under practical agricultural operating conditions.

Overall, the Attention-Enhanced YOLOv8n-CLS provides a favorable balance between classification performance and computational cost for the dataset-defined tomato leaf categories evaluated in this study. The comparative benchmarking, ablation analysis, cross-validation, cross-dataset evaluation, computational assessment, and Grad-CAM analysis provide complementary evidence regarding the performance and behavior of the proposed framework within the evaluated experimental setting. Nevertheless, the framework should currently be regarded as an image-based symptom classification and preliminary disease-screening approach rather than a definitive pathogen diagnostic system or field-validated agricultural deployment solution. Further research involving laboratory-confirmed disease status, independently collected field images, mixed-infection cases, diverse cultivars and disease stages, and representative agricultural edge hardware is required to establish biological specificity, field generalization, and operational deployment feasibility.

## 5 Conclusion and future directions

### 5.1 Conclusion

This study presented an Attention-Enhanced YOLOv8n-CLS framework for image-based tomato leaf disease classification using image-level supervision. A lightweight spatial attention mechanism was incorporated into the YOLOv8n-CLS architecture to enhance the representation of spatially informative image features while maintaining a compact computational structure. The framework was evaluated using the PlantVillage Tomato Dataset and the Kaggle Tomato Leaf Disease Dataset through binary and multiclass classification experiments, comparative benchmarking, ablation analysis, five-fold cross-validation, computational assessment, cross-dataset evaluation, and Grad-CAM-based visualization.

The proposed model achieved a maximum classification Accuracy of 98.90% in the evaluated classification experiments, together with an AUC of 0.991, a computational complexity of 7.5 GFLOPs, and an average inference speed of approximately 45 FPS on the evaluated computing platform. In the five-fold cross-validation analysis, the proposed model achieved a mean Accuracy of 98.50% ± 0.14% on the PlantVillage Tomato Dataset and 97.82% ± 0.29% on the Kaggle Tomato Leaf Disease Dataset. Compared with the evaluated benchmark models, including ResNet50, DenseNet201, ResNet152V2, MobileNetV2, and YOLOv5, the proposed framework demonstrated a favorable balance between classification performance and computational requirements under the evaluated experimental conditions. The ablation analysis further indicated that incorporation of the lightweight spatial attention mechanism was associated with improved classification performance relative to the corresponding baseline configuration.

The statistical analysis accounted for the paired structure of the five-fold cross-validation experiments. Because identical fold assignments were used for all evaluated models, the fold-wise observations were treated as paired rather than independent observations. The Friedman test was used as the omnibus non-parametric test for overall model comparison, followed by pairwise Wilcoxon signed-rank tests with Holm–Bonferroni correction where applicable. This approach provided a statistical comparison appropriate to the matched cross-validation design while avoiding the assumption that the five folds constituted independent observations. Nevertheless, the five folds represent alternative data partitions within the same datasets rather than five independent biological samples, independently collected datasets, or independent field experiments. Therefore, the statistical findings should be interpreted as evidence of comparative model performance within the specified cross-validation framework rather than as evidence of independent biological replication.

The cross-dataset experiments were conducted using the 10 categories shared by the two datasets, with the Kaggle-specific Powdery Mildew category excluded from this analysis. The proposed model achieved 96.2% Accuracy when trained on PlantVillage and evaluated on Kaggle, and 96.8% Accuracy when trained on Kaggle and evaluated on PlantVillage. These findings provide evidence of transferability between the two evaluated dataset distributions. However, cross-dataset evaluation should not be considered equivalent to field validation because publicly available datasets may not represent the full variability encountered under agricultural conditions, including differences in cultivar, leaf age, disease stage, symptom severity, illumination, background, environmental stress, imaging equipment, and naturally occurring infections.

The Grad-CAM analysis provided qualitative information regarding the image regions associated with the model’s classification decisions. In the representative examples, the highlighted regions generally corresponded to visually symptomatic portions of the leaves. These visualizations provide additional insight into the spatial basis of the model’s predictions but do not establish pathogen-specific causality, confirm the biological correctness of a prediction, or independently validate the dataset-provided labels. Accordingly, Grad-CAM should be regarded as an interpretability technique rather than as an independent diagnostic validation method.

The scope of the proposed framework should also be distinguished from laboratory-based pathogen diagnosis. The model learns associations between input images and the disease categories supplied by the source datasets, whereas the present study did not independently confirm causal pathogens using molecular, serological, microbiological, biochemical, or other laboratory-based methods. Consequently, the reported Accuracy, Precision, Recall, F1-score, and AUC describe agreement between model predictions and the dataset-provided image categories rather than laboratory-confirmed pathogen identification. Predictions for viral categories such as Tomato mosaic virus and Tomato yellow leaf curl virus should therefore be interpreted as recognition of associated visual symptom patterns rather than definitive confirmation of viral infection.

The single-label formulation represents another limitation of the current framework. The datasets used in this study do not provide independently verified annotations for mixed infections, and the present study therefore does not establish how the model performs when multiple pathogens or disease conditions occur simultaneously on the same leaf or plant. Under such conditions, a single-label classifier may assign an image to the category whose learned visual characteristics are most strongly represented while failing to identify secondary or less visually prominent symptoms. Consequently, the ability of the proposed framework to recognize mixed or co-occurring disease conditions remains to be established.

The characteristics of the evaluated datasets also limit the extent to which the findings can be generalized to field conditions. PlantVillage provides comparatively standardized image-acquisition conditions, whereas the Kaggle dataset provides greater variation in image appearance. Nevertheless, publicly available image datasets may be affected by selection and sampling effects and may not represent the complete range of symptom expression encountered in naturally infected plants. In addition, consistent plant- or leaf-level identifiers were not available for the datasets used in this study; therefore, the possibility of related or near-duplicate images occurring across dataset partitions cannot be completely excluded. Factors such as cultivar, leaf age, disease stage, symptom severity, environmental conditions, background, illumination, imaging equipment, and disease prevalence may influence model performance. Accordingly, the present results should be interpreted as evidence obtained under the evaluated dataset conditions rather than as comprehensive validation under agricultural field conditions.

The computational measurements indicate that the proposed architecture has potential for resource-constrained image-classification applications. However, the reported approximately 45 FPS inference speed was obtained on the evaluated computing platform and does not represent performance measured on a dedicated agricultural edge device. Similarly, the study did not include systematic field validation using independently collected agricultural images. Consequently, hardware-specific latency, memory utilization, power consumption, thermal behavior, and sustained field performance remain to be established.

Overall, the Attention-Enhanced YOLOv8n-CLS provides a promising approach for image-based tomato leaf symptom classification and preliminary disease screening within the evaluated datasets. The proposed framework achieved strong classification performance while maintaining a relatively compact computational profile, and the cross-dataset experiments provided additional evidence of transferability between the two evaluated datasets. However, the framework should currently be regarded as an image-based symptom classification and preliminary screening approach rather than a definitive pathogen diagnostic system or field-validated agricultural deployment solution. Definitive pathogen confirmation and operational agricultural deployment require additional biological, field, and hardware validation.

### 5.2 Future directions

Future research should first evaluate the proposed framework using independently collected field images obtained under diverse agricultural conditions. Such datasets should include variation in illumination, background, camera characteristics, cultivar, leaf age, disease stage, symptom severity, and environmental stress. Where feasible, image samples should be accompanied by laboratory-confirmed disease or pathogen status to determine the extent to which image-based predictions correspond to biologically confirmed infections.

The current single-label formulation could also be extended to multi-label classification to represent simultaneous or overlapping disease conditions. Evaluation using laboratory-confirmed single infections and mixed infections would provide a more realistic assessment of model behavior when multiple pathogens or disease conditions occur on the same plant or leaf. Disease localization and severity estimation through object detection or semantic segmentation could additionally provide spatial information that is not available from image-level classification alone.

Practical deployment should be investigated on representative agricultural edge platforms, including low-power embedded AI devices. Such experiments should measure inference latency, memory utilization, power consumption, thermal characteristics, and sustained processing performance under realistic operating conditions. Model-compression techniques such as pruning, quantization, and knowledge distillation could also be explored to further reduce computational and memory requirements while retaining useful classification performance.

The framework could be extended to additional tomato disease categories and other economically important crops. Multimodal approaches combining RGB imagery with hyperspectral, thermal, or UAV-based observations may provide complementary information when visible symptoms are ambiguous. Domain-adaptation and continual-learning techniques could further be investigated to address changes in geographical location, environmental conditions, imaging devices, cultivar distributions, and disease prevalence.

Future studies should also investigate the robustness of the proposed framework under uncontrolled field conditions, including variations in image quality, partial leaf occlusion, complex backgrounds, multiple leaves within a single image, variable symptom severity, and overlapping disease symptoms. Such evaluations would help determine whether the performance observed on curated public datasets can be maintained when the model encounters the greater variability present in practical agricultural environments.

Further research should investigate independent repeated training runs using multiple random seeds to quantify training-to-training variability separately from the variation observed across cross-validation folds. Such experiments would provide additional evidence regarding reproducibility and would distinguish variability arising from alternative data partitions from variability associated with stochastic model initialization and optimization.

These research directions would allow the proposed framework to progress from evaluation on publicly available image datasets toward more comprehensive validation using biologically verified samples and operational agricultural environments. Such validation is necessary to establish its practical value as an image-based crop-health screening system while maintaining a clear distinction between automated symptom classification and laboratory-confirmed pathogen diagnosis.

## Data Availability

The original contributions presented in the study are included in the article/supplementary material. Further inquiries can be directed to the corresponding authors.
